# Identification of 11 candidate structured noncoding RNA motifs in humans by comparative genomics

**DOI:** 10.1186/s12864-021-07474-9

**Published:** 2021-03-09

**Authors:** Lijuan Hou, Jin Xie, Yaoyao Wu, Jiaojiao Wang, Anqi Duan, Yaqi Ao, Xuejiao Liu, Xinmei Yu, Hui Yan, Jonathan Perreault, Sanshu Li

**Affiliations:** 1 Medical School, Molecular Medicine Engineering and Research Center of Ministry of Education, Key Laboratory of Precision Medicine and Molecular Diagnosis of Fujian Universities, Institute of Genomics, School of Biomedical Sciences, Huaqiao University, Xiamen, 361021 P. R. China; 2 INRS - Institut Armand-Frappier, 531 boul des Prairies, Laval, Québec H7V1B7 Canada

**Keywords:** Comparative genomics, Structured ncRNAs, Human genomes, Animal genomes, Pipeline

## Abstract

**Background:**

Only 1.5% of the human genome encodes proteins, while large part of the remaining encodes noncoding RNAs (ncRNA). Many ncRNAs form structures and perform many important functions. Accurately identifying structured ncRNAs in the human genome and discovering their biological functions remain a major challenge.

**Results:**

Here, we have established a pipeline (CM-line) with the following features for analyzing the large genomes of humans and other animals. First, we selected species with larger genetic distances to facilitate the discovery of covariations and compatible mutations. Second, we used CMfinder, which can generate useful alignments even with low sequence conservation. Third, we removed repetitive sequences and known structured ncRNAs to reduce the workload of CMfinder. Fourth, we used Infernal to find more representatives and refine the structure. We reported 11 classes of structured ncRNA candidates with significant covariations in humans. Functional analysis showed that these ncRNAs may have variable functions. Some may regulate circadian clock genes through poly (A) signals (PAS); some may regulate the elongation factor (EEF1A) and the T-cell receptor signaling pathway by cooperating with RNA binding proteins.

**Conclusions:**

By searching for important features of RNA structure from large genomes, the CM-line has revealed the existence of a variety of novel structured ncRNAs. Functional analysis suggests that some newly discovered ncRNA motifs may have biological functions. The pipeline we have established for the discovery of structured ncRNAs and the identification of their functions can also be applied to analyze other large genomes.

**Supplementary Information:**

The online version contains supplementary material available at 10.1186/s12864-021-07474-9.

## Background

The Human Genome Project shows that only 1.5% of the human genome encodes proteins. The subsequent ENCODE project showed that approximately 75% of the human genome can be transcribed into RNAs, of which 74% are non-protein-encoded RNAs (ncRNAs) [1–3].

Some of these ncRNAs form complex structures that perform important biological functions. tRNA [4] forms a clover structure, and carries amino acids to participate in protein synthesis; ribosomal RNA (rRNA) [5] and RNase P [6] are also structured ncRNAs, which catalyze peptide bond formation and process tRNA precursors respectively; SRP (Signal recognition particle) RNA [7], also known as 7SL is also a structured ncRNA, which directs the traffic of proteins within the cell; riboswitches [8] also regulate gene expression by forming complex structures that bind to metabolites or other small molecules.

Different methods and algorithms have been developed to search for structured ncRNAs. One approach is to look at large gaps between protein-coding regions and highly conserved sequences among closely related bacterial species [9]. Another approach is to look for structured ncRNAs from guanine and cytosine rich (GC-rich) regions in an organism with a high adenine and thymine (AT) genomic sequence [10]. Similarly, orphan promoters or terminators that are not directly related to the coding regions have been used to identify structured ncRNAs [9, 11].

Recently, several algorithms including QRNA [12], RNAz [13], CMfinder [14], and Evofold [15] have been successfully developed or applied to search for structured ncRNAs. In 2004, RNAz was used to discover conserved noncoding elements from humans, mice, rats, *Fugu* and zebrafish [13]. In 2006, Evofold was used to find 48,000 structured ncRNAs in eight vertebrates (estimated false-positive rate reached 62%) [15]. In 2008, CMfinder was applied to search the ENCODE [2] regions of vertebrate multiple alignments, and 6587 candidates were found (estimated false positive rate reached 50%) [16]. In 2010 and 2011, Evofam was applied (a pipeline driven by Evofold) to find 220 groups of structured ncRNAs in 29 mammals and the function of several groups were predicted [17]. In 2013, RNAz and SiSSiz were used for the prediction of structured ncRNAs in 35 mammalian genomes and many candidates were found [18]. In 2017, CMfinder was used to predict the RNA structure of 17 mammalian genomes and 510,000 human structured ncRNAs were predicted [19].

As early as 2004, the group of Breaker [20–27] began using CMfinder to discover structured ncRNAs called riboswitches. To date, they have discovered hundreds of structured ncRNAs and verified more than 35 classes of riboswitches [8, 28]. In 2017, a pipeline containing CMfinder [14], Infernal [29] and other algorithms (such as RNAcode [30]) was used to discover new structured ncRNAs from fungal genomes [31]. 15 well-structured RNAs were found, including variant HDV self-cleaving ribozymes, atypical snoRNAs, and motifs associated with various protein-encoding genes (ribosomal proteins, HexA and others). It was demonstrated that among these motifs, the *SDC* RNA motif could reduce gene expression. In addition, similar pipelines were applied to discover three novel classes of self-cleaving ribozymes [26]. These examples show that CMfinder is a powerful algorithm for finding stable structured ncRNAs.

In the current study, we have modified the old pipeline which was driven by CMfinder and had been used for fungal ncRNA discovery [31] to find structured ncRNAs from large genomes such as the human genome. We compared the human genome to the genomes of 26 eukaryotic species, including some protist species and metazoan species, to increase the evolutionary distance to facilitate the discovery of covariations. We also used WindowMasker [32] to remove repeat sequences to reduce the data volume. The structure quality of each candidate has been assessed based on the identification of significant covariations by R-scape [33], and the identification of compatible mutations and the complexity of the structure predicted by CMfinder and R2R [34]. The best structured ncRNA candidates were selected for further functional analyses.

The functional analyses include the assay of the genomic location of the motif and its associated upstream and downstream genes, the RegRNA assay [35], the RBPmap assay [36], the TCGA analysis [37, 38], the STRING analysis [39], and others. The goal is to find out whether they are riboswitches, ribozymes, transcription and translation regulators, and so on. For example, the discoveries of the long-distance regulation of alternative splicing by fungal TPP riboswitches [40], the eukaryotic fluoride channel proteins [41, 42], ligands for the *yjdF* riboswitch [43], and three novel classes of natural ribozymes (Hatchet, pistol and twister sister) [26, 44, 45] were all based on functional analyses.

Through our pipeline, many structured ncRNA candidates in humans were discovered, and 11 of the selected candidates are reported here. These ncRNAs have many different functions. Some may regulate circadian clock genes by cooperating with poly (A) signals (PAS), some may regulate elongation factors by cooperating with RNA binding proteins, some may regulate T-cell receptor signaling pathways. Importantly, our pipeline can also be applied to analyze other large genomes to discover structured ncRNAs and the identification of their functions.

## Results

### Identification of candidate structured ncRNAs

**Fig. 1:**
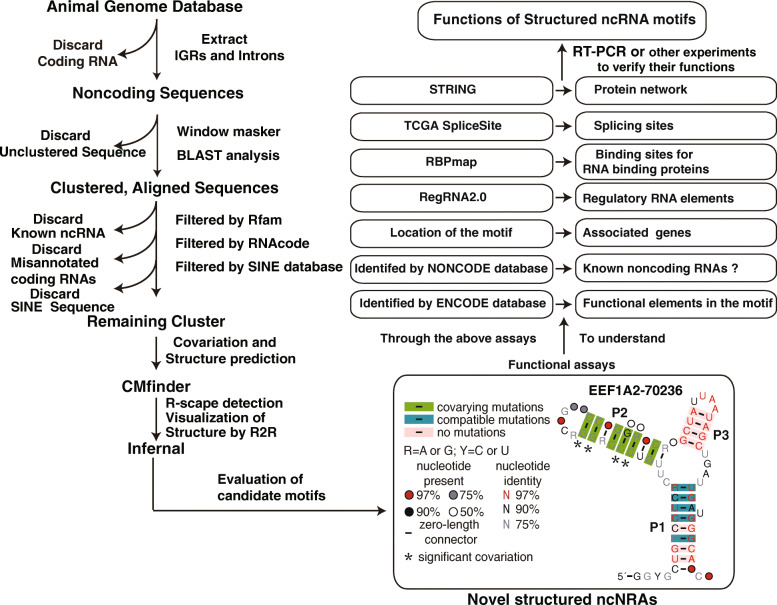
Workflow for the discovery of novel human structured ncRNAs and their functional analyses. See the description for each step in the Materials and Methods. Candidate ncRNA *EEF1A2–70,236* is shown as an example. Functional analyses include several categories and their different purposes. P1 through P6 designate pairing elements (or stems), and other annotations are listed beside the consensus structure

Promising ncRNA candidates were identified by applying our computational pipeline to compare the human genomes with selected 23 metazoan and three protist genomes (Table S1 in the additional file 1). The selection includes a wide range of species to increase the genetic distance and thereby facilitate the discovery of covariation. Briefly, the process (Fig. 1) involved extracting noncoding intergenic regions (IGRs) and intronic sequences based on genomic annotations, using BLAST [46] to align similar sequences to form clusters, and filtering to remove known ncRNAs, mis-annotated coding RNAs and transposable elements such as Short Interspersed Elements (SINEs) sequences. The remaining clusters were subjected to analysis with CMfinder [14] to find variations and compatible mutations in the aligned sequences and predict the secondary structure of RNA candidates. Finally, Infernal [29] was used to search for additional representatives from a database including all metazoan (vertebrates and invertebrates) and protozoan genomes (Table S3 in the additional file 1) and refine the consensus model.

### Selection of RNA candidates with structures

After the prediction of RNA structures by CMfinder, we analyzed the RNA candidates based on structure-related information such as covariations, compatible mutations and continuous base-pairings in the stem regions, the number of loops or stems to indicate the structural complexity (Fig. S2 a in the Additional file 2). In addition, the number of representatives and gene association with each candidate was investigated when considering its biological function. Covariation refers to those pairings in which when one nucleotide on one side is mutated, the nucleotide on the other side is mutated correspondingly to maintain the pairing of the two nucleotides [14]. Compatible mutations refer to those pairings in which one side nucleotide mutation is sufficient to maintain nucleotides pairing (for example, changing A-U pairing to G-U pairing) [14](Fig. S2 a in the Additional file 2). The goal is to select some ncRNAs that have conserved structures.

Through the pipeline containing CMfinder (which we call CM-line), 15,151 candidates were discovered (during the process, Rfam RNA homologues and SINE RNA homologues were removed). Then, R-scape [33] which used a statistical approach to specifically evaluate the significance of covariations in a given alignment was applied to detect significant covariations in these motifs. R-scape predicted that 11,763 motifs (78% of total) (Fig. S2 b in the Additional file 2 and Stockholm files in the Additional file 19, 20, 21, 22 and 23) possessed at least one statistically significant covariation with an e-value less than 0.05 (with -s --fold) (Statistically significant covariations are marked with stars beside the base-pairings). From the 11,763 candidate motifs predicted by CM-line, we performed the following steps to select some of the most promising motifs. First, we manually screen RNAs with covariations and compatible mutations (the more the better); Second, we selected motifs with a certain structural complexity (several stem-loops). Many functional RNAs such as riboswitches, ribozymes, tRNAs have complex structures. RNA candidates with complex structures and covariations will be retained. After the selection, we are left with thousands of candidate RNAs. Third, we use Infernal to search for more representatives with similar structure and sequence conservation. Please note that Infernal requires a lot of computer resources and runs for a long time. Therefore, we decided to perform Infernal at this stage to save time, rather than at an earlier stage. The main purpose is to find more representatives from the large genome database. We choose motifs that are representative and widely distributed among different species. The number of candidates dropped further (down to hundreds). Fourth, we look at gene locations to find their gene association and functional assays. In this stage, in order to ensure that the candidate is not part of the known ncRNA (or overlapped too much), we searched in NONCODE and ENCODE to find the functional elements in the motif and whether they belong to NONCODE. If so, we would drop them. RegRNA2.0 also helps to identify functional elements in these candidates and whether they are known ncRNAs. Finally, we reported 11 RNA candidates that we believe they are most likely to serve as structured ncRNA candidates.

### Structural and functional assays of RNA candidates

Below are described the most promising 11 candidate motifs that likely serve as functional ncRNAs. The alignments including sequences and species for each sequence for these motifs in Stockholm format are presented in the Additional files 3, 4, 5, 6, 7, 8, 9, 10, 11, 12 and 13 and the Additional file 17 (pictures for each alignment). Their sequences in fasta format are in the Additional file 18. files The motif name is usually as simple as motif_70,236. The number 70236 was produced by the pipeline CM-line during the discovery process. However, if the associated genes for the motif are known, the name for the motif is composed of a gene name and a number such as *EEF1A2–70,236*.

### Structured ncRNA candidates

#### The EEF1A2–70,236 motif

**Fig. 2:**
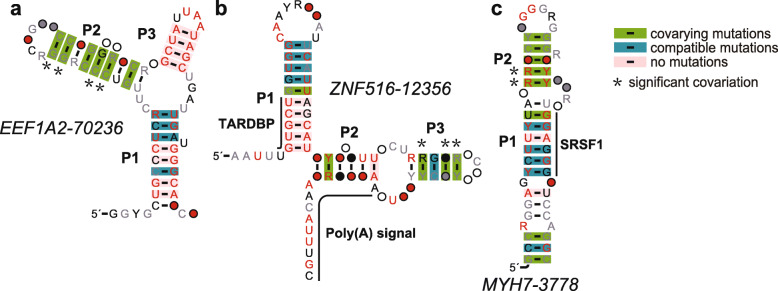
Candidates for structured RNA motifs (group I). Consensus sequence and secondary structure of three ncRNAs are shown in the figure. **a**. *EEF1A2–70,236*. **b**. *ZNF516–12356*. **c**. *MYH7–3778*. Green and blue boxes present that those base-pairings are covariations and compatible mutations respectively (reported by R2R [34] or by manual detection). Significant covariations (detected by R-scape [33]) (E-value < 0.05) were labeled with stars beside each base-pair. The methods of covariation detection were described in the Methods

The *EEF1A2–70,236* motif (Fig. 2 a) is represented by 105 examples from 77 species (humans and other metazoans) (Stockholm files for each motif are listed in the Additional files 3, 4, 5, 6, 7, 8, 9, 10, 11, 12 and 13; the basic information of each motif is listed in Table S3 (in the additional file 1), including species, related genes, and genomic location. The diagram of genomic locus for human motifs is in Fig. S3 (in the additional file 14)). The motif is typically forming a three-stem junction (Fig. 2 a) with many covariations in the P2 stem, four of which are significant covariations detected by R-scape [33].

This motif in many other species appears to be in the intron of the gene *EEF1A2* (eukaryotic translation elongation factor 1 alpha 2). The human example of this motif is located in the 2th intron of the gene *EEF1A2* on the chromosome 20 (NCBI BLAST (version 2.2.25) [46]), which is expressed in many tissues and is likely to be critical in the development of ovarian cancer [47].

In order to analyze RNA regulatory elements that are embedded in the RNA motif, we used a web-based RegRNA [35] system to identify the functional RNA motifs and sites in an input RNA sequence by integrating many analysis algorithms such as Infernal and MiRanda [48]. RegRNA analysis predicted a conserved intron splicing enhancer (ISE) 5′AGCUGAU residing between the P1 and P3 stems.

Here, we used RBPmap [36] to show the conserved RNA binding sites on the RNA motif. RBPmap used an algorithm for mapping the motifs, based on a Weighted-Rank approach, which considers the clustering propensity of the binding sites. We used the web-based RBPmap to analyze many sequences of the RNA motif and found that the RNA binding protein SRSF2 can bind to the conserved sequence (5′UGCCCUC) in the P1 stem of the motif. SRSF2 is a member of the serine/arginine (SR)-rich family of pre-mRNA splicing factors [49]. In the UCSC browser [50, 51], the RNA motif is about 300 nucleotides (nts) away from the nearest splicing site. One hypothesis about the function of this motif could be that the RNA motif cooperates with SRSF2 to regulate gene expression by regulating the alternative splicing of the introns.

#### The *ZNF516–12356* motif

The *ZNF516–12356* motif consensus model is based on 120 examples from 102 species. Note that some of the examples have the same sequences although they are from different species. The secondary structure includes two hairpins with the support of seven covariations, three of which are significant covariations detected by R-scape [33], indicating that this motif might form a structured RNA (Fig. 2 b).

The human example of this motif is located in the 3rd intron of the gene *ZNF516* on the chromosome 18, encoding a zinc finger protein, which is a transcriptional regulator that binds to the gene promotor to control brown adipose tissue (BAT) differentiation, thereby regulating thermogenesis [52]. The cancer genome atlas (TCGA) [37, 38] analysis showed that the *ZNF516* gene is highly expressed in certain cancer types and is a potential suppressor of EGFR, which is related to breast carcinogenesis [53]. RegRNA2.0 analysis [35] showed that the 3′ end of the motif contained a polyadenylation signal (PAS) such as 5′ ATTAAAAGAAAGATTTGC. RBPmap [36] predicted that protein TARDBP (TAR DNA Binding Protein) can potentially bind to the conserved region (5′ UGUGCU) of the motif. TARDBP is a nuclear DNA/RNA-binding protein involved in RNA metabolism, and is a pathological hallmark of amyotrophic lateral sclerosis (ALS) [54]. One hypothesis about the function of this motif could be that this binding protein may cooperate with the RNA motif to regulate the availability of the polyadenylation signal domain to regulate mRNA processing.

#### The *MYH7–3778* motif

A total of 72 sequences of the *Motif-3778* motif have been identified from 34 species. The consensus sequence and secondary structure model based on these sequences reveal that this motif may form a long hairpin structure with several bulges (Fig. 2 c). P1 region contains several compatible mutations while P2 stem region contains five covariations and two of them are significant covariations, indicating that this motif might form a structured RNA.

The human example of this RNA motif is in the intron of the gene encoding myosin heavy chain 7 (MYH7) on the chromosome 14. According to UCSC browser, this intron is alternatively spliced. It is complementary to part of the NONCODE [55] RNA transcript NONHSAT233552.1. This heavy chain constitutes the fundamental contractile subunit of skeletal and cardiac muscle [56]. Mutations in this gene might lead to distal myopathy [56].

RBPmap [36] predicted that the RNA binding protein SRSF1 can bind to the conserved region of the motif at 5′GGAGGG in the P1 stem. SRSF1 encodes an arginine/serine-rich splicing factor, and binds to purine-rich RNA sequences to constitute a powerful splicing enhancer [57]. Therefore, one hypothesis about the function of this RNA motif may regulate the availability of the binding site for SRSF1.

#### The ACVR2B-21,548 motif

**Fig. 3:**
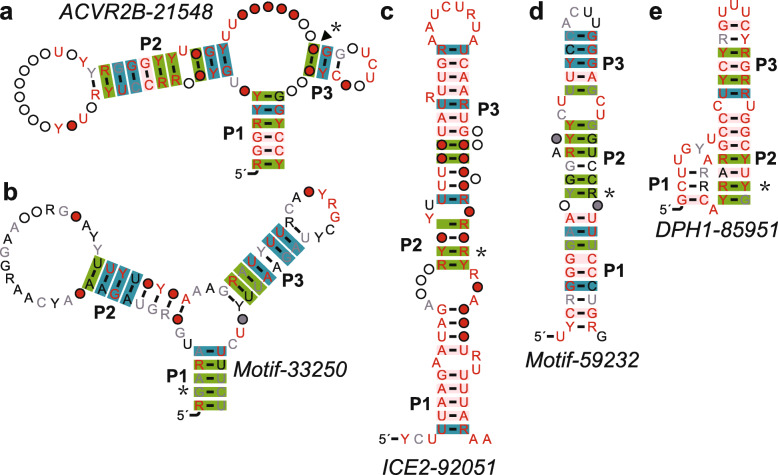
Candidates for structured RNA motifs (group II). Consensus sequence and secondary structure of five ncRNAs are shown in the figure. **a**. *ACVR2B-21,548*. **b**. *Motif-33,250*. **c**. *ICE2–92051*. **d**. *Motif-59,232*. **e**. *DPH1–85951*. Annotations are as described in the Fig. 2

The *ACVR2B-21,548* motif includes 33 examples from 18 species. The predicted secondary structure includes three hairpins with a three-way junction, which is supported by a few covariations and some compatible mutations (Fig. 3 a). One of the covariations is statistically significant at an E-value of 0.05.

Two human examples of this motif are both located in the 5th intron region of the *ACVR2B* gene on the chromosome 3 (by NCBI BLAST), which encodes a transmembrane serine/threonine kinase activin type-2 receptor, transducing the activin signal from the cell surface to the cytoplasm to regulate many biological processes [58]. According to USCS, this is not an alternatively spliced intron, but the exon right next to it can be alternatively spliced. In addition, a small part (10 nts) of the human examples appears to overlap a SINE element shown on UCSC browser.

Both RegRNA and RBPmap predicted that some of the sequences of the motif might have transcriptional regulatory [59] motifs and RNA binding protein binding sites. However, since these regions are not highly conserved, we did not further investigate these elements.

#### The ANTXR2–33250 motif

The *ANTXR2–33250,* represented by 75 sequences from 53 species (humans and other metazoans). The consensus sequence and secondary structure model based on these sequences reveal that this motif may form a structure with a three-stem junction, supported by several covariations and compatible mutations (Fig. 3 b). The human example of the motif is located in the intron of the gene (on the chromosome 4) encoding ANTXR cell adhesion molecule 2 (ANTXR2), which is a receptor for anthrax toxin and may be involved in extracellular matrix adhesion and tissue remodeling [59]. According to UCSC browser, the instance on the human chromosome 4 can be alternatively spliced, but the alternative “intron” encompasses a few exons, including the RNA Motif-33,250 roughly in the middle.

#### The *ICE2–92051* motif

A total of 20 sequences of the *ICE2–92051* motif have been identified from 11 species. The consensus sequence and secondary structure model based on these sequences suggest that this motif may form a long hairpin with some bulges and internal loops (Fig. 3 c). The human example of this motif is in the gene encoding interactor of little elongation complex ELL subunit 2 (ICE2). ICE2 plays a role in small nuclear RNA (snRNA) transcription by enhancing both RNA Polymerase II occupancy and transcriptional elongation [60]. According to UCSC browser, the intron of the human example is alternatively spliced (with many different outcomes in terms of final mature RNAs).

RegRNA predicted that 13 sequences of the motif contain polyadenylation signal sequences (PAS). If the predicted PAS is true and it is used by the RNA motif, it is likely that RNA spliced isoforms use this PAS as polyadenylation processing signals and therefore the RNA motif may interact with this PAS to regulate gene expression.

#### The Motif-59,232 motif

Represented by 55 distinct examples from 43 species, the motif typically forms a long hairpin structure with one internal loop (Fig. 3 d), supported by several covariations in the middle of the structure. One of them is a significant covariation. Many examples are located in a region about 3000 base-pairs away from a gene called DLX4, which encodes a protein that is postulated to play a role in forebrain and craniofacial development in drosophila [61].

Both RegRNA and RBPmap predicted that some of the sequences of the motif might have transcriptional regulatory motifs, intron splicing enhancers (ISE) and RNA binding protein binding sites. However, since these regions are not highly conserved, we did not further investigate these elements.

#### The *DPH1–85951* motif

The *DPH1–85951* motif is represented by 19 examples from 15 species, typically forming a simple hairpin with two bulges (Fig. 3 e). The human example of this motif is located in the 4th intron of the gene encoding an enzyme involved in the biosynthesis of diphthamide, a modified histidine found only in elongation factor-2 (EEF2) [62]. According to USCS, the human instance on chromosome 17 can be alternatively spliced.

RBPmap [36] predicted that the RNA binding protein PTBP1 (Polypyrimidine Tract Binding Protein 1) can bind to the conserved region of the human motif at 5′GUUUUCU around the loop region. PTBP1 plays a role in pre-mRNA splicing by regulating alternative splicing events [63].

#### The ARNTL-1638 motif

**Fig. 4:**
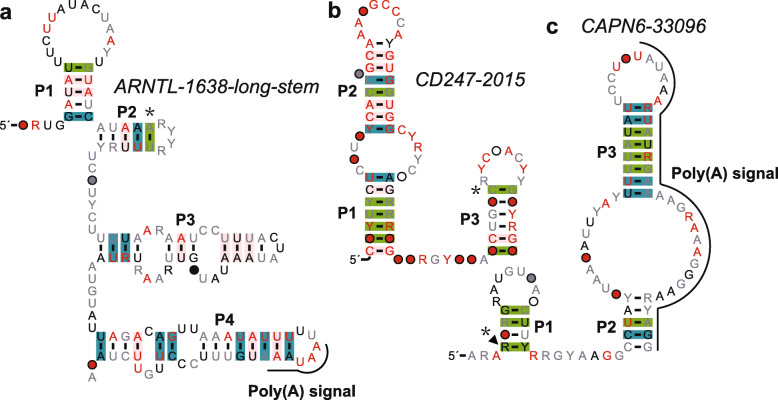
Candidates for structured RNA motifs (group III). Consensus sequence and secondary structure of three ncRNAs are shown in the figure. **a**. *ARNTL-1638*. **b**. *CD247–2015*. **c**. *CAPN6–33096*. Annotations are as described in the Fig. 2

Represented by 215 distinct examples from 170 species, the motif typically forms a four-hairpin structure (Fig. 4 a). All examples are located in the 3′ UTR (untranslated region) of the gene ARNTL, which encodes a core clock protein called BMAL1. BMAL1 is a helix-loop-helix domain that interacts with CLOCK to form heterodimers and regulates the expression of other core clock genes PER1, PER2, PER3, CRY1 and CRY2 [64]. ARNTL gene dysfunction may lead to changes in gluconeogenesis, fatty acid production, sleep patterns and immune responses [65, 66].

According to RegRNA2.0 [35] analysis, the ARNTL motif contains the 5′AAUAAAUGUUUCCCUUGUUYUANA polyadenylation signal sequence (PAS), which forms the P3 hairpin of the RNA motif, indicating that this motif can control the availability of PAS. The fact that ESTs of what appears to be the most abundant 3′ end show that the poly (A) is found immediately after this PAS further supports that this is the most used PAS for this gene.

#### The *CD247–2015* motif

The *CD247–2015* motif includes 42 examples from 31 species. The predicted secondary structure includes two hairpins with one internal loop, which is supported by seven covariations and three compatible mutations, one of which is a significant covariation (Fig. 4 b). The motif is located in the second intron region of the *CD247* gene, which encodes T-cell receptor zeta, a protein that forms the T-cell receptor-CD3 complex [67]. According to UCSC browser, this intron can be alternatively spliced (in some version of mature mRNA, the intron is included in the final mRNA). The receptor zeta plays an important role in coupling antigen recognition to several intracellular signal-transduction pathways [67].

RBPmap analysis predicted that the human sequences of the motif might contain a conserved binding site (5′ UGGUGGC in the P2 stem) for RNA binding protein SRSF1. Since SRSF1 is an arginine/serine-rich splicing factor and is a powerful splicing enhancer [57], the function of this RNA motif could be that it may regulate the availability of the binding site for SRSF1 and affect alternative splicing, as hypothesized for *motif-3778*, which also has a binding site for SRSF1.

#### The CAPN6–33096 motif

The consensus model of the *CAPN6–33096* motif is based on 79 examples from 60 species. Its secondary structure includes two stems and one big internal loop, of which the formation of P1 and P2 stems is supported by seven covariations and five compatible mutations. One of the covariations is significant (Fig. 4 c). The human example of this motif is located in the second intron of the gene CAPN6 (Calpain 6), which lacks the active-site catalytic cysteine residue of a protease, and is involved in microtubule stabilization and cytoskeletal organization during embryogenesis [68].

RegRNA analysis predicted that most of the examples of the *CAPN6–33096* motif contain polyadenylation signal sequences at the end of the RNA motif. If the prediction is true, then this PAS may be used to determine the formation of short (partial) or long mRNA (full length).

#### Expression of selected motifs

To investigate whether these RNA motifs are expressed in the cells, whether these motifs affect the splicing patterns of their pre-mRNAs and whether their gene expression levels are as high as those of house-keeping genes, we use Reverse transcriptase (RT) PCR to detect the gene expression.

**Fig. 5:**
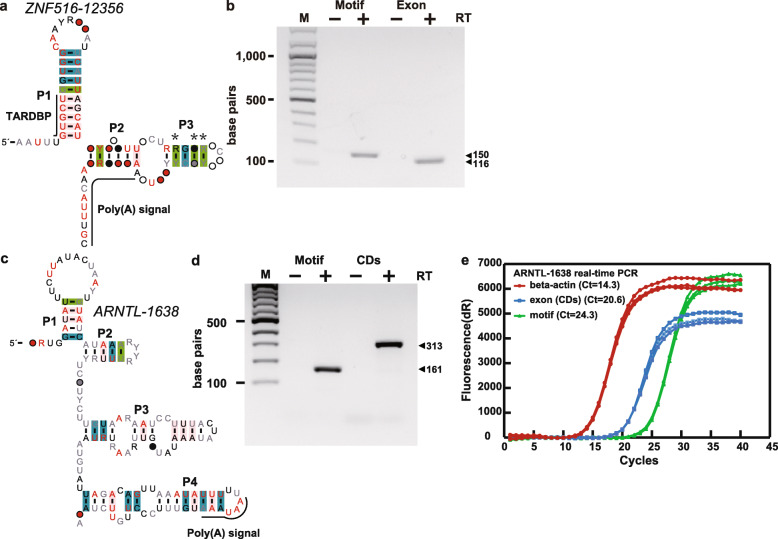
The *ZNF516–12356* and *ARNTL-1638* motifs and their expression. **a** Consensus sequence and secondary structure of the *ZNF516–12356* motif with annotated protein binding sites and poly (A) signal sequences. **b** Agarose gel separation of RT-PCR products of *ZNF516–12356* motif, generated by using primers for the RNA motif itself (Motif) and mRNA coding regions (Exon) (note: The full-length gel is in Fig. S4 [see Additional file 15]). M stands for DNA markers. Lanes containing PCR products are marked with (+) or (−), indicating the presence or absence of reverse transcriptase (RT). The two arrows next to the gel indicate bands corresponding to DNA products of the expected size. **c** Consensus sequence and secondary structure of the *ARNTL-1638* motif. **d** Agarose gel separation of RT-PCR products of *ARNTL-1638* motif (note: The full-length gel is in Fig. S4 [see Additional file 15]). The poly (A) signal is shown in the figure. Gel annotations are as described for b. **e** Real-time PCR results were performed using primers for the RNA motif (in the intron) and the exon containing the RNA motif (β-actin RNA was used as a control for constitutively expressed genes). The experiment was repeated twice and the results were similar

RT-PCR results showed that the primers targeting the *ZNF516–12356* motif and the exons adjacent to the motif could produce the expected PCR products, indicating that the cell can produce this motif and the exon RNAs (Fig. 5a, b and Fig. S4 [Additional file 15. Please note that Fig. S4 showed the full length of gel pictures of Fig. 5.]).

Similarly, RT-PCR showed that the primers targeting the *ARNTL-1638* motif and the exons next to the motif produced the expected PCR products, indicating that the cell can also produce this motif and its associated exon RNAs (Fig. 5c, d and Fig. S4 [see Additional file 15]). More in-vivo experiments are required to test whether this motif is involved in the circadian clock regulation.

In addition, real-time PCR showed that the expression of the RNA motif was much lower than the house-keeping gene (*β-actin*) in the case of the *ARNTL-1638* motif (Fig. 5e) and the *NPTN-6924* motif (Fig. S5 in the Additional file 16, the full-length of the original gel is in the Fig. S6 (in the Additional file 25)). The real-time PCR for the *ZNF516–12356* motif was not available (not cycle threshold (C_t_) was detected) due to unknown reasons.

Regarding the effect of RNA motifs on pre-mRNA splicing patterns, we did not observe alternative splicing products during the RT-PCR for the motif *ZNF516–12356* and the *NPTN-6924* motif, which are located in the introns. However, during my previous discovery of the fungal RNA motifs, RT-PCR of the intronic RNA motif (the *rpl7* motif) produced an alternative splicing product [31].

In order to better understand the mechanism of RNA function, further experiments such as Northern blotting, RNA structural analysis (in-line probing [69], SHAPE [70], etc), and in-vivo reporter construction will be required.

## Discussion

### Establishing a pipeline to explore the human genome for structured ncRNAs

About 20 years ago, the first metabolite sensing structured RNA which was coined as riboswitch was discovered by Breaker’s lab [23]. Later several riboswitches were discovered by solving the puzzles in some published papers which reported mysterious RNAs involved in metabolite-dependent regulation of gene expression without the need of proteins [24, 71–74]. With the help of bioinformatics, notably with CMfinder, prokaryote genomes were screened for structured RNA motifs. Remarkably, hundreds of new structured ncRNAs were discovered, from which at least 35 classes of riboswitches and five new classes of ribozymes were verified [20–22, 26, 75, 76]. These discoveries indicate that structured ncRNAs play an important role in fundamental biological processes. Around 2009, a pipeline similar to the bacteria bioinformatics procedure that includes the CMfinder algorithm was established to discover structured ncRNAs from fungal genomes [31], and 15 candidate structured noncoding RNA motifs and their functions were identified. In the current study, we further modified the pipeline (which we now call “CM-line”) to be suitable for the discovery of structured ncRNAs from higher organisms such as humans and other metazoans. We have established the pipeline by comparing the human genome with 26 selected eukaryotes to include sufficient genetic distance to reveal covariations and compatible mutations which could indicate the presence of a structured RNA. Then, we used infernal to find more representatives that have similar structures in protists and metazoans.

### Discovery of structured ncRNA candidates and identifying their functions

Over the past few years, other groups also have applied CMfinder [14] and other algorithms such as Evofold [15] to discover structured ncRNAs and conserved elements in human and certain vertebrates. We noticed that most of their effort was to find as many candidates as possible. However, our main goal is to establish a pipeline to discover conserved structured RNA candidates with additional layers of bioinformatics analysis to help determine their functions.

To identify the function of an RNA motif, one of the important steps is to identify the RNA regulatory elements that are embedded inside or near the RNA motif. These regulatory elements in eukaryotes include RNA processing sites, poly (A) signals (PAS), splicing sites, binding sites for RBPs, intron or exon enhancers or repressors, miRNA target sites, etc. These elements could be utilized by RNA motifs to fulfill their functions, therefore identification of these elements will help infer the functions of RNA motifs.

Another important step is to find genes that are associated with these RNA motifs. Riboswitches are often involved in genes involved in metabolite pathways directly related to their cognate ligand. The ribosomal protein-associated RNA motifs found in fungi are usually located in noncoding regions of genes encoding ribosomal proteins [31]. Ribozymes are commonly not associated with a single type of gene, with the exception of the *glmS* ribozyme [76], which is both a riboswitch and a ribozyme and is associated mostly with cell wall synthesis genes.

Through the study of the functions of riboswitches, we speculate that many of the motifs we identified in humans may bind to proteins to regulate gene expression in a manner similar to riboswitch regulation. However, in these cases, one RBP could bind to the RNA motif, and the binding could alter the conformation of the expression platform, such as an alternative splicing site or a poly (A) signal sequence to regulate the gene expression.

### Covariations reported by R2R and R-scape

The covariations reported in many earlier studies [21, 26, 31, 75] were based on the structure (described in a Stockholm file) predicted by CMfinder [14] and reported by R2R. Although R2R doesn’t use a statistical method to test the significance of the covariations, it helps draw the secondary structures of the RNAs and show the covariations, compatible mutations, as well as base-pairs without mutations [34]. CMfinder and R2R have helped to discover many structured ncRNAs such as riboswitches, ribozymes and many other candidates [20, 21, 26, 27, 75, 77]. In 2017, R-scape [33] used a statistical approach to specifically evaluate the significance of covariations in an alignment by using a random model to look for phylogenetic signals. There is a detailed discussion about the difference of the approaches to report covariations by R2R and R-scape in Weinberg’s paper [78].

However, other subtle mutations can also be used to indicate the formation of RNA structures, especially for those closely related species. Covariations in the structured RNAs are produced during evolution [79]. They can be produced from the G-C pair to the A-U pair, or from the A-U pair to the G-C pair. Both pathways may be intermediated by the G-U pair [79]. Therefore, it is easier to form compatible mutations than covariations. If the evolutionary distance between species was too short, covariations will not be observed, but compatible mutations will be observed. For structured ncRNA searches in eukaryotes, if we only look for ncRNAs with significant covariations, we may lose the opportunity to find many interesting structured ncRNAs, because the evolutionary distance between eukaryotes may be too short. This will result in the inability to observe covariations, and there are not many sequenced eukaryotic species. We did observe some motifs potentially forming complex structures with pseudoknots although there were no significant covariations predicted (data not shown).

Hence, we used both R2R and R-scape to report covariations because we believe each has its advantage especially when we compared these two methods to predict covariations for purine and TPP riboswitches. However, eleven ncRNA candidates reported here all contain at least one significant covariations.

### Discovery of important functions for new structured ncRNA motifs

We found that the *ARNTL-1638*, *ZNF516–12356*, *ICE2–92051* and *CAPN6–33096* motifs contain a poly (A) signal (PAS) embedded in their structure. These motifs might regulate the availability of the PAS to regulate the stability of mRNAs. The *CD247–2015* motif may regulate the formation of T-cell receptor-CD3 complex to recognize intracellular signals. However, at this stage, more bioinformatics data and the use of more extensive biological experimental procedures are still needed to further understand their functions.

## Conclusions

Many ncRNAs have been identified in the three domains of life. Many ncRNAs form structures and perform a diversity of important biological functions. However, identifying structured ncRNAs in the human genome and discovering their biological functions are still challenging.

In this work, we have established the CM-line with a CMfinder algorithm, making it suitable for analyzing large genomes of humans and other animals. Through CM-line, we have discovered many structured ncRNA candidates in humans, and report 11 of the selected candidates here. These ncRNAs have various functions. Some may regulate circadian clock genes by cooperating with poly (A) signals (PAS), some may regulate elongation factors by cooperating with RNA binding proteins, some may regulate T-cell receptor signaling pathways. The pipeline we have established for the discovery of structured ncRNAs and identification of their functions can also be applied to analyze other large genomes.

## Methods

### Identification of candidate RNA motifs

The human genome, 23 metazoan and three protist genomes (Table S1 in the additional file 1) were downloaded from NCBI [80]. Since a large part of human and eukaryotic genomes are repetitive elements, to avoid the discovery of many repetitive elements, the NCBI WindowMasker [32] was applied to mark the repetitive sequences in genomes. The pipeline (Fig. 1) established in this study was similar to that used previously to identify numerous structured ncRNAs in fungi [31]. Briefly, the intergenic regions (IGRs) and introns of each annotated gene were extracted from all genomes by applying a Perl script. IGRs and introns were then aligned by NCBI BLAST (version 2.2.25) [46] using parameters –W7 –e 1e–6. Similar sequences forming clusters were identified by a Perl script based on the BLAST results. Each cluster was initially filtered using BLAST against the Rfam database (version 14.1) [81] (Later, for those final candidates, Infernal was used to search the Rfam database) and the SINE database [82] to remove any known RNAs with parameters –e 1e–9. RNAcode [30] was used to further filter candidate clusters to remove those candidates with a *P*-value cutoff of 0.001, indicating that they are unlikely to encode proteins. The remaining clusters were fed into CMfinder [14] to predict RNA structures. Based on the predicted structure including covariations (evaluated by R-scape [33]) and compatible mutations, as well as the number of representatives and the complexity of the structure, some candidate RNA motifs were selected for further analyses. Infernal [29] was used to search for more homologs of these RNA motifs among genomes of protozoan and metazoan species (Additional file 1 Table S2) according to both sequence and structure similarity and to refine the structure of these RNA motifs. The list of species for each motif is included at the top part of the Stockholm file after Infernal search (Additional files 3, 4, 5, 6, 7, 8, 9, 10, 11, 12 and 13). Identical sequences from different species found by Infernal were kept in the Stockholm files. To make sure the novelty of the motifs, each selected motif was checked by Rfam webpage server [81] to get rid of those having homologous RNAs in Rfam. We then performed a functional analysis for these selected motifs. We analyzed the genomic location of each motif, its associated upstream and downstream genes, RNA regulatory elements or conserved elements in the motif, conserved RBP binding sites, and others as listed in the flow chart in Fig. 1 (Flow chart steps containing the configuration information of each software program are in the Additional file 24). The goal is to evaluate which type of genetic element these structured ncRNA motifs may be, such as ribozymes, alternative splicing or translational regulators.

### Covariation evaluation

Several algorithms have been developed to assess the covariations in sequence alignments. However, currently available algorithms cannot consistently handle problems such as incorrect alignments which could predict invalid covariation [78]. We have previously evaluated covariation manually, including with the help of CMfinder and R2R [31]. R2R [34] reports covariation when there are at least two Watson-Crick or G-U base pairs and more than 90% of the sequences contain the canonical Watson-Crick base pairs at those positions. However, R2R’s annotations are not statistically validated, and were therefore not used to draw conclusions about candidates [78]. Recently, R-scape [33] has used a statistical approach to evaluate the significance of covariations in an alignment by using a random model to look for phylogenetic signals that can be used for covariation analysis at a given E-value (such as 0.05). We analyzed all motifs using R-scape v1.5.1 with the -s option.

When using both R-scape [33] and R2R [34] for reporting covariations for the purine riboswitch (Fig. S1) [see Additional file 2], R-scape reported five significant covariations in P1 stem, while R2R reported four covariations. Three of these covariations were the same. In addition, both R-scape and R2R report similar covariations in P2 and P3 for the purine riboswitch. For the TPP riboswitch, covariations in P2 were the same, while R-scape reports more covariations in P1, P3 and P5 stems. However, R-scape did not report any covariations in the P4 stem but R2R did. These two cases indicated that the predictions of covariations by these two algorithms are similar and can cooperate with each other in some degree. So, for the diagrams, we also use both R-scape and R2R to depict potential covariations. To allow manual or other analyses of our predicted RNA candidates, we provide our sequence alignments in Stockholm format (see Additional files 3, 4, 5, 6, 7, 8, 9, 10, 11, 12 and 13).

### Assessing the novelty of motifs

To determine whether the predicted structured RNA candidates were reported previously, we used Infernal to compare them with known RNA motifs in the Rfam database [81]. Novel RNA candidates are those that are not found in Rfam, or highly homologous to any known RNAs in other known databases such as SINEBase [82]. If a motif belongs to NONCODE [55] (mostly lincRNAs) or CRS-regions (sequence-conserved elements) [16], it will be annotated. Notably, a structured RNA motif appearing frequently in our search was later found to be an Alu element in the SINEBase but it was not annotated in the genome databases we downloaded.

### Cell cultures

Cell culture follows standard protocol [83]. Briefly, cell lines HEK-293 (**human embryonic kidneys**) (purchased from Conservation Genetics CAS Kunming Cell Bank, Kunming, Yunnan, China) were passaged twice before collecting cells for RNA extraction. HEK293 were cultured in DMEM medium and supplemented with 10% fetal bovine serum, 100 mg/mL penicillin, and 100 units/mL streptomycin (Gibco, ThermoFisher Scientific). Cells were maintained in a humidified 37 °C incubator with 5% CO_2_.

### RNA extraction

RNA extraction was performed by using the TIANGEN RNAprep Kit (TIANGEN Biotech Co., LTD) and following the manufacturer’s protocol.

### Reverse transcriptase polymerase chain reaction (RT-PCR)

RT-PCR was performed as previously described [31]. For motifs in an intron, we used one pair of primers to target the motif and used another pair of primers to target exons upstream and downstream of the motif. Table S4 (in the Additional file 1) lists the primer sequences and the expected sizes of the RT-PCR products. During RT-PCR, a negative control, in which the RT reaction lacked reverse transcriptase, was included to ensure there is no DNA contamination. RT-PCR used RNA extracted from the HEK293 cell line.

### Real-time PCR

Real-time PCR was performed on the Agilent Mx3000P real-time PCR system using the TransStart® Top Green qPCR SuperMix kit according to the manufacturer’s instructions (Transgen Biotech, Beijing, China).

## Supplementary Information

**Additional file 1 Table S1.** Species, their classification and links to genome download sites (from NCBI). **Table S2.** Other related databases and their download sites. **Table S3.** Motif basic information. **Table S4.** Primers and product sizes.

**Additional file 2 Fig. S1.** Comparison of covariations reported by R2R and R-scape. **Fig. S2.** Analysis of structured ncRNA candidates.

**Additional file 3.** Motif ACVR2B_21,548 Stockholm file.

**Additional file 4.** Motif ARNTL-1638 Stockholm file.

**Additional file 5.** Motif CAPN6_33,096 Stockholm file.

**Additional file 6.** Motif CD247_2015 Stockholm file.

**Additional file 7.** Motif DPH1_85,951 Stockholm file.

**Additional file 8.** Motif EEF1A2_70,236 Stockholm file.

**Additional file 9.** Motif ICE2_92,051 Stockholm file.

**Additional file 10.** Motif_33,250 Stockholm file.

**Additional file 11.** Motif_59,232 Stockholm file.

**Additional file 12.** Motif MYH7_3778 Stockholm file.

**Additional file 13.** Motif ZNF516–12356 Stockholm file.

**Additional file 14 Fig. S3.** Genomic locus diagram.

**Additional file 15 Fig. S4.** The full-length original agarose gels for Fig. 5.

**Additional file 16 Fig. S5.** Real-time PCR for the *NPTN-6924* motif.

**Additional file 17.** Pictures of sequence alignments for 11 RNA motifs.

**Additional file 18:.** txt file for 11 motifs.

**Additional file 19:.** txt file for all 11,763 motifs part1.

**Additional file 20:.** txt file for all 11,763 motifs part2.

**Additional file 21:.** txt file for all 11,763 motifs part3.

**Additional file 22:.** txt file for all 11,763 motifs part4.

**Additional file 23:.** txt file for all 11,763 motifs part5.

**Additional file 24.** Bioinformatics steps and configurations.

**Additional file 25.** Fig. S6. The full-length original agarose gel for Fig. S5b.

## Data Availability

The datasets (such as genomes) were downloaded from NCBI or other public sites. Links are included in the Table S1 and S2 [see Additional file 1]. Most data generated or analyzed during this study are included in this published article. Stockholm files and other related files for the reported 11 motifs are in the additional files [see Additional files 3, 4, 5, 6, 7, 8, 9, 10, 11, 12 and 13]. The remaining Stockholm files are available from the corresponding author on request.

## Abbreviations

PAS: Poly (A) signals
EEF1A: Elongation factor
IGRs: Intergenic regions
SINEs: Short Interspersed Elements
BAT: Brown adipose tissue
TCGA: The cancer genome atlas
TARDBP: TAR DNA Binding Protein
ALS: Amyotrophic lateral sclerosis
MYH7: Myosin heavy chain 7
ANTXR2: ANTXR cell adhesion molecule 2
ICE2: Elongation complex ELL subunit 2
ISE: Intron splicing enhancers
EEF2: Elongation factor-2
PTBP1: Polypyrimidine Tract Binding Protein 1
CAPN6: Calpain 6

## References

[1] Venter JC, Adams MD, Myers EW, Li PW, Mural RJ, Sutton GG, Smith HO, Yandell M, Evans CA, Holt RA (2001). The sequence of the human genome. Science.

[2] Consortium EP, Birney E, Stamatoyannopoulos JA, Dutta A, Guigo R, Gingeras TR, Margulies EH, Weng Z, Snyder M, Dermitzakis ET (2007). Identification and analysis of functional elements in 1% of the human genome by the ENCODE pilot project. Nature.

[3] Lander ES, Linton LM, Birren B, Nusbaum C, Zody MC, Baldwin J, Devon K, Dewar K, Doyle M, FitzHugh W (2001). Initial sequencing and analysis of the human genome. Nature.

[4] Schattner P, Brooks AN, Lowe TM (2005). The tRNAscan-SE, snoscan and snoGPS web servers for the detection of tRNAs and snoRNAs. Nucleic Acids Res.

[5] Nissen P, Hansen J, Ban N, Moore PB, Steitz TA (2000). The structural basis of ribosome activity in peptide bond synthesis. Science.

[6] Guerrier-Takada C, McClain WH, Altman S (1984). Cleavage of tRNA precursors by the RNA subunit of E. coli ribonuclease P (M1 RNA) is influenced by 3′-proximal CCA in the substrates. Cell.

[7] Keenan RJ, Freymann DM, Stroud RM, Walter P (2001). The signal recognition particle. Annu Rev Biochem.

[8] Breaker RR. Riboswitches and the RNA world. Cold Spring Harb Perspect Biol. 2012;4:a003566. 10.1101/cshperspect.a003566 PMC3281570 21106649

[9] Argaman L, Hershberg R, Vogel J, Bejerano G, Wagner EG, Margalit H, Altuvia S (2001). Novel small RNA-encoding genes in the intergenic regions of Escherichia coli. Curr Biol.

[10] Klein RJ, Misulovin Z, Eddy SR (2002). Noncoding RNA genes identified in AT-rich hyperthermophiles. Proceed Nat Acad Sci U S A.

[11] Olivas WM, Muhlrad D, Parker R (1997). Analysis of the yeast genome: identification of new non-coding and small ORF-containing RNAs. Nucleic Acids Res.

[12] Rivas E, Eddy SR (2001). Noncoding RNA gene detection using comparative sequence analysis. BMC bioinformatics.

[13] Washietl S, Hofacker IL, Stadler PF (2005). Fast and reliable prediction of noncoding RNAs. Proceed Nat Acad Sci U S A.

[14] Yao Z, Weinberg Z, Ruzzo WL (2006). CMfinder--a covariance model based RNA motif finding algorithm. Bioinformatics.

[15] Pedersen JS, Bejerano G, Siepel A, Rosenbloom K, Lindblad-Toh K, Lander ES, Kent J, Miller W, Haussler D (2006). Identification and classification of conserved RNA secondary structures in the human genome. PLoS Comput Biol.

[16] Torarinsson E, Yao Z, Wiklund ED, Bramsen JB, Hansen C, Kjems J, Tommerup N, Ruzzo WL, Gorodkin J (2008). Comparative genomics beyond sequence-based alignments: RNA structures in the ENCODE regions. Genome Res.

[17] Parker BJ, Moltke I, Roth A, Washietl S, Wen J, Kellis M, Breaker R, Pedersen JS (2011). New families of human regulatory RNA structures identified by comparative analysis of vertebrate genomes. Genome Res.

[18] Smith MA, Gesell T, Stadler PF, Mattick JS (2013). Widespread purifying selection on RNA structure in mammals. Nucleic Acids Res.

[19] Seemann SE, Mirza AH, Hansen C, Bang-Berthelsen CH, Garde C, Christensen-Dalsgaard M, Torarinsson E, Yao Z, Workman CT, Pociot F (2017). The identification and functional annotation of RNA structures conserved in vertebrates. Genome Res.

[20] Weinberg Z, Wang JX, Bogue J, Yang J, Corbino K, Moy RH, Breaker RR (2010). Comparative genomics reveals 104 candidate structured RNAs from bacteria, archaea, and their metagenomes. Genome Biol.

[21] Weinberg Z, Lünse CE, Corbino KA, Ames TD, Nelson JW, Roth A, Perkins KR, Sherlock ME, Breaker RR (2017). Detection of 224 candidate structured RNAs by comparative analysis of specific subsets of intergenic regions. Nucleic Acids Res.

[22] Weinberg Z, Barrick JE, Yao Z, Roth A, Kim JN, Gore J, Wang JX, Lee ER, Block KF, Sudarsan N (2007). Identification of 22 candidate structured RNAs in bacteria using the CMfinder comparative genomics pipeline. Nucleic Acids Res.

[23] Nahvi A, Sudarsan N, Ebert MS, Zou X, Brown KL, Breaker RR (2002). Genetic control by a metabolite binding mRNA. Chem Biol.

[24] Winkler W, Nahvi A, Breaker RR (2002). Thiamine derivatives bind messenger RNAs directly to regulate bacterial gene expression. Nature.

[25] Barrick JE, Corbino KA, Winkler WC, Nahvi A, Mandal M, Collins J, Lee M, Roth A, Sudarsan N, Jona I (2004). New RNA motifs suggest an expanded scope for riboswitches in bacterial genetic control. Proceed Nat Acad Sci USA.

[26] Weinberg Z, Kim PB, Chen TH, Li S, Harris KA, Lunse CE, Breaker RR (2015). New classes of self-cleaving ribozymes revealed by comparative genomics analysis. Nat Chem Biol.

[27] Breaker RR (2011). Prospects for riboswitch discovery and analysis. Mol Cell.

[28] McCown PJ, Corbino KA, Stav S, Sherlock ME, Breaker RR (2017). Riboswitch diversity and distribution. Rna.

[29] Nawrocki EP, Eddy SR (2013). Infernal 1.1: 100-fold faster RNA homology searches. Bioinformatics.

[30] Washietl S, Findeiss S, Muller SA, Kalkhof S, von Bergen M, Hofacker IL, Stadler PF, Goldman N (2011). RNAcode: robust discrimination of coding and noncoding regions in comparative sequence data. Rna.

[31] Li S, Breaker RR (2017). Identification of 15 candidate structured noncoding RNA motifs in fungi by comparative genomics. BMC Genomics.

[32] Morgulis A, Gertz EM, Schäffer AA, Agarwala R (2006). WindowMasker: window-based masker for sequenced genomes. Bioinformatics.

[33] Rivas E, Clements J, Eddy SR (2017). A statistical test for conserved RNA structure shows lack of evidence for structure in lncRNAs. Nat Methods.

[34] Weinberg Z, Breaker RR (2011). R2R--software to speed the depiction of aesthetic consensus RNA secondary structures. BMC bioinformatics.

[35] Chang TH, Huang HY, Hsu JB, Weng SL, Horng JT, Huang HD (2013). An enhanced computational platform for investigating the roles of regulatory RNA and for identifying functional RNA motifs. BMC bioinformatics.

[36] Paz I, Kosti I, Ares M, Cline M, Mandel-Gutfreund Y (2014). RBPmap: a web server for mapping binding sites of RNA-binding proteins. Nucleic Acids Res.

[37] Wang Z, Jensen MA, Zenklusen JC (2016). A practical guide to the Cancer genome atlas (TCGA). Methods Mol Biol.

[38] Hutter C, Zenklusen JC (2018). The Cancer genome atlas: creating lasting value beyond its data. Cell.

[39] Szklarczyk D, Gable AL, Lyon D, Junge A, Wyder S, Huerta-Cepas J, Simonovic M, Doncheva NT, Morris JH, Bork P (2019). STRING v11: protein-protein association networks with increased coverage, supporting functional discovery in genome-wide experimental datasets. Nucleic Acids Res.

[40] Li S, Breaker RR (2013). Eukaryotic TPP riboswitch regulation of alternative splicing involving long-distance base pairing. Nucleic Acids Res.

[41] Li S, Smith KD, Davis JH, Gordon PB, Breaker RR, Strobel SA (2013). Eukaryotic resistance to fluoride toxicity mediated by a widespread family of fluoride export proteins. Proceed Nat Acad Sci USA.

[42] Li S, Breaker RR (2012). Fluoride enhances the activity of fungicides that destabilize cell membranes. Bioorg Med Chem Lett.

[43] Li S, Hwang XY, Stav S, Breaker RR (2016). The yjdF riboswitch candidate regulates gene expression by binding diverse azaaromatic compounds. Rna.

[44] Harris KA, Lunse CE, Li S, Brewer KI, Breaker RR (2015). Biochemical analysis of pistol self-cleaving ribozymes. Rna.

[45] Li S, Lunse CE, Harris KA, Breaker RR (2015). Biochemical analysis of hatchet self-cleaving ribozymes. Rna.

[46] Altschul SF, Madden TL, Schäffer AA, Zhang J, Zhang Z, Miller W, Lipman DJ (1997). Gapped BLAST and PSI-BLAST: a new generation of protein database search programs. Nucleic Acids Res.

[47] Anand N, Murthy S, Amann G, Wernick M, Porter LA, Cukier IH, Collins C, Gray JW, Diebold J, Demetrick DJ (2002). Protein elongation factor EEF1A2 is a putative oncogene in ovarian cancer. Nat Genet.

[48] John B, Enright AJ, Aravin A, Tuschl T, Sander C, Marks DS (2004). Human MicroRNA targets. PLoS Biol.

[49] Luo C, Cheng Y, Liu Y, Chen L, Liu L, Wei N, Xie Z, Wu W, Feng Y (2017). SRSF2 regulates alternative splicing to drive hepatocellular carcinoma development. Cancer Res.

[50] Kent WJ, Sugnet CW, Furey TS, Roskin KM, Pringle TH, Zahler AM, Haussler D (2002). The human genome browser at UCSC. Genome Res.

[51] Haeussler M, Zweig AS, Tyner C, Speir ML, Rosenbloom KR, Raney BJ, Lee CM, Lee BT, Hinrichs AS, Gonzalez JN (2019). The UCSC genome browser database: 2019 update. Nucleic Acids Res.

[52] Dempersmier J, Sambeat A, Gulyaeva O, Paul SM, Hudak CS, Raposo HF, Kwan HY, Kang C, Wong RH, Sul HS (2015). Cold-inducible Zfp516 activates UCP1 transcription to promote browning of white fat and development of brown fat. Mol Cell.

[53] Li L, Liu X, He L, Yang J, Pei F, Li W, Liu S, Chen Z, Xie G, Xu B (2017). ZNF516 suppresses EGFR by targeting the CtBP/LSD1/CoREST complex to chromatin. Nat Commun.

[54] Nishino K, Watanabe S, Shijie J, Murata Y, Oiwa K, Komine O, Endo F, Tsuiji H, Abe M, Sakimura K (2019). Mice deficient in the C-terminal domain of TAR DNA-binding protein 43 develop age-dependent motor dysfunction associated with impaired Notch1-Akt signaling pathway. Acta Neuropathologica Communications.

[55] Zhao Y, Li H, Fang S, Kang Y, Wu W, Hao Y, Li Z, Bu D, Sun N, Zhang MQ (2016). NONCODE 2016: an informative and valuable data source of long non-coding RNAs. Nucleic Acids Res.

[56] Meredith C, Herrmann R, Parry C, Liyanage K, Dye DE, Durling HJ, Duff RM, Beckman K, de Visser M, van der Graaff MM (2004). Mutations in the slow skeletal muscle fiber myosin heavy chain gene (MYH7) cause laing early-onset distal myopathy (MPD1). Am J Hum Genet.

[57] Jobbins AM, Reichenbach LF, Lucas CM, Hudson AJ, Burley GA, Eperon IC (2018). The mechanisms of a mammalian splicing enhancer. Nucleic Acids Res.

[58] Olsen OE, Wader KF, Hella H, Mylin AK, Turesson I, Nesthus I, Waage A, Sundan A, Holien T (2015). Activin a inhibits BMP-signaling by binding ACVR2A and ACVR2B. Cell communication and signaling : CCS.

[59] Greither T, Wedler A, Rot S, Keßler J, Kehlen A, Holzhausen HJ, Bache M, Würl P, Taubert H, Kappler M. CMG2 expression is an independent prognostic factor for soft tissue sarcoma patients. Int J Mol Sci. 2017;18:2648. 10.3390/ijms18122648 PMC5751250 29215551

[60] Hu D, Smith ER, Garruss AS, Mohaghegh N, Varberg JM, Lin C, Jackson J, Gao X, Saraf A, Florens L (2013). The little elongation complex functions at initiation and elongation phases of snRNA gene transcription. Mol Cell.

[61] Wu D, Mandal S, Choi A, Anderson A, Prochazkova M, Perry H, Gil-Da-Silva-Lopes VL, Lao R, Wan E, Tang PL (2015). DLX4 is associated with orofacial clefting and abnormal jaw development. Hum Mol Genet.

[62] Dong M, Dando EE, Kotliar I, Su X, Dzikovski B, Freed JH, Lin H (2019). The asymmetric function of Dph1-Dph2 heterodimer in diphthamide biosynthesis. J Biol Inorganic Chem.

[63] Georgilis A, Klotz S, Hanley CJ, Herranz N, Weirich B, Morancho B, Leote AC, D'Artista L, Gallage S, Seehawer M (2018). PTBP1-mediated alternative splicing regulates the inflammatory Secretome and the pro-tumorigenic effects of senescent cells. Cancer cell.

[64] Kriebs A, Jordan SD, Soto E, Henriksson E, Sandate CR, Vaughan ME, Chan AB, Duglan D, Papp SJ, Huber AL (2017). Circadian repressors CRY1 and CRY2 broadly interact with nuclear receptors and modulate transcriptional activity. Proceed Nat Acad Sci USA.

[65] Woon PY, Kaisaki PJ, Braganca J, Bihoreau MT, Levy JC, Farrall M, Gauguier D (2007). Aryl hydrocarbon receptor nuclear translocator-like (BMAL1) is associated with susceptibility to hypertension and type 2 diabetes. Proceed Nat Acad Sci USA.

[66] Sato TK, Yamada RG, Ukai H, Baggs JE, Miraglia LJ, Kobayashi TJ, Welsh DK, Kay SA, Ueda HR, Hogenesch JB (2006). Feedback repression is required for mammalian circadian clock function. Nat Genet.

[67] Ye W, Zhou Y, Xu B, Zhu D, Rui X, Xu M, Shi L, Zhang D, Jiang J (2019). CD247 expression is associated with differentiation and classification in ovarian cancer. Medicine.

[68] Tonami K, Kurihara Y, Aburatani H, Uchijima Y, Asano T, Kurihara H (2007). Calpain 6 is involved in microtubule stabilization and cytoskeletal organization. Mol Cell Biol.

[69] Regulski EE, Breaker RR (2008). In-line probing analysis of riboswitches. Methods Mol Biol.

[70] Wilkinson KA, Merino EJ, Weeks KM (2006). Selective 2′-hydroxyl acylation analyzed by primer extension (SHAPE): quantitative RNA structure analysis at single nucleotide resolution. Nat Protoc.

[71] Winkler WC, Cohen-Chalamish S, Breaker RR (2002). An mRNA structure that controls gene expression by binding FMN. Proceed Nat Acad Sci USA.

[72] Mandal M, Boese B, Barrick JE, Winkler WC, Breaker RR (2003). Riboswitches control fundamental biochemical pathways in Bacillus subtilis and other bacteria. Cell.

[73] Sudarsan N, Wickiser JK, Nakamura S, Ebert MS, Breaker RR (2003). An mRNA structure in bacteria that controls gene expression by binding lysine. Genes Dev.

[74] Winkler WC, Nahvi A, Sudarsan N, Barrick JE, Breaker RR (2003). An mRNA structure that controls gene expression by binding S-adenosylmethionine. Nat Struct Biol.

[75] Roth A, Weinberg Z, Chen AG, Kim PB, Ames TD, Breaker RR (2014). A widespread self-cleaving ribozyme class is revealed by bioinformatics. Nat Chem Biol.

[76] Winkler WC, Nahvi A, Roth A, Collins JA, Breaker RR (2004). Control of gene expression by a natural metabolite-responsive ribozyme. Nature.

[77] Sherlock ME, Sudarsan N, Breaker RR (2018). Riboswitches for the alarmone ppGpp expand the collection of RNA-based signaling systems. Proceed Nat Acad Sci USA.

[78] Eckert I, Weinberg Z (2020). Discovery of 20 novel ribosomal leader candidates in bacteria and archaea. BMC Microbiol.

[79] Dutheil J, Pupko T, Jean-Marie A, Galtier N (2005). A model-based approach for detecting coevolving positions in a molecule. Mol Biol Evol.

[80] O'Leary NA, Wright MW, Brister JR, Ciufo S, Haddad D, McVeigh R, Rajput B, Robbertse B, Smith-White B, Ako-Adjei D (2016). Reference sequence (RefSeq) database at NCBI: current status, taxonomic expansion, and functional annotation. Nucleic Acids Res.

[81] Nawrocki EP, Burge SW, Bateman A, Daub J, Eberhardt RY, Eddy SR, Floden EW, Gardner PP, Jones TA, Tate J (2015). Rfam 12.0: updates to the RNA families database. Nucleic Acids Res.

[82] Vassetzky NS, Kramerov DA (2013). SINEBase: a database and tool for SINE analysis. Nucleic Acids Res.

[83] Guo P, Zhang J, Chrzanowski M, Huang J, Chew H, Firrman JA, Sang N, Diao Y, Xiao W (2019). Rapid AAV-neutralizing antibody determination with a cell-binding assay. Mol Ther Methods Clin Dev.

