# Supplementary material for: Identification of 11 candidate structured noncoding RNA motifs in humans by comparative genomics
Source: BMC Genomics. 2021 Mar 9;22:164. doi: 10.1186/s12864-021-07474-9 (PMC7941889; doi:10.1186/s12864-021-07474-9)
Supplement: Supplementary file 1 — Additional file 1 Table S1. Species, their classification and links to genome download sites (from NCBI). Table S2. Other related databases and their download sites. Table S3. Motif basic information. Table S4. Primers and product sizes. [file 12864_2021_7474_MOESM1_ESM.pdf]

Table S1 Species, their classification and links to genome download sites (from NCBI)

No.	Species	Classification	Sites for genome download
1	homo_sapiens (Human)	Phylum: Chordata<br>Class: Mammalia<br>Order: Primates<br>Family: Hominidae<br>Genus: Homo<br>Species: H. sapiens	ftp://ftp.ncbi.nlm.nih.gov/genomes/all/GCF/000/001/405/GCF_000001405.38_GRCh38.p12/GCF_000001405.38_GRCh38.p12_genomic.gbff.gz
2	Anolis_carolinensis (Green Anole)	Phylum: Chordata<br>Class: Reptilia<br>Order: Squamata<br>Family: Dactyloidae<br>Genus: Anolis<br>Species: A. Carolinensis	ftp://ftp.ncbi.nlm.nih.gov/genomes/all/GCF/000/090/745/GCF_000090745.1_AnoCar2.0/GCF_000090745.1_AnoCar2.0_genomic.gbff.gz
3	Gallus_gallus (Chicken**)**	Phylum: Chordata<br>Class: Aves<br>Order: Galliformes<br>Family: Phasianidae<br>Genus: Gallus<br>Species: G. Gallus	ftp://ftp.ncbi.nlm.nih.gov/genomes/all/GCF/000/002/315/GCF_000002315.5_GRCg6a/GCF_000002315.5_GRCg6a_genomic.gbff.gz
4	Danio_rerio (Zebrafish)	Phylum: Chordata<br>Class: Actinopterygii<br>Order: Cypriniformes<br>Family: Cyprinidae<br>Genus: Danio<br>Species: D. Rerio	ftp://ftp.ncbi.nlm.nih.gov/genomes/all/GCF/000/002/035/GCF_000002035.6_GRCz11/GCF_000002035.6_GRCz11_genomic.gbff.gz
5	Mus_musculus (Mouse)	Phylum: Chordata<br>Class: Mammalia<br>Order: Rodentia<br>Family: Muridae<br>Genus: Mus<br>Species: M. Musculus	ftp://ftp.ncbi.nlm.nih.gov/genomes/all/GCF/000/001/635/GCF_000001635.26_GRCm38.p6/GCF_000001635.26_GRCm38.p6_genomic.gbff.gz
6	Patiria miniate (Sea star)	Phylum: Echinodermata<br>Class: Asteroidea<br>Order: Valvatida<br>Family: Asterinidae<br>Genus: Patiria<br>Species: P. Miniata	ftp://ftp.ncbi.nlm.nih.gov/genomes/all/GCA/000/285/935/GCA_000285935.1_Pmin_1.0/GCA_000285935.1_Pmin_1.0_genomic.gbff.gz
7	Hymenolepis	Phylum: Platyhelminthes	ftp://ftp.ncbi.nlm.nih.gov/genomes/all/GCA/000/469/805/GCA_0

	microstoma<br>(Tapeworm)	Class: Cestoda<br>Order: Cyclophyllidea<br>Family: Hymenolepididae<br>Genus: Hymenolepis<br>Species: H. Microstoma	00469805.2_HMIC002/GCA_000469805.2_HMIC002_genomic.gbff.gz
8	Mnemiopsis leidyi<br>(Warty comb jelly)	Phylum: Ctenophora<br>Class: Tentaculata<br>Order: Lobata<br>Family: Bolinopsidae<br>Genus: Mnemiopsis Agassiz<br>Species: M. Leidyi	ftp://ftp.ncbi.nlm.nih.gov/genomes/all/GCA/000/226/015/GCA_000226015.1_MneLei_Aug2011/GCA_000226015.1_MneLei_Aug2011_genomic.gbff.gz
9	Ixodes scapularis<br>(Deer tick)	Phylum: Arthropoda<br>Class: Arachnida<br>Order: Ixodida<br>Family: Ixodidae<br>Genus: Ixodes<br>Species: I. Scapularis	ftp://ftp.ncbi.nlm.nih.gov/genomes/all/GCF/000/208/615/GCF_000208615.1_JCVI_ISG_i3_1.0/GCF_000208615.1_JCVI_ISG_i3_1.0_genomic.gbff.gz
10	Helobdella robusta<br>(Leech)	Phylum: Annelida<br>Class: Clitellata<br>Order: Rhynchobdellida<br>Family: Glossiphoniidae<br>Genus: Helobdella<br>Species: H. Robusta	ftp://ftp.ncbi.nlm.nih.gov/genomes/all/GCF/000/326/865/GCF_000326865.1_Helobdella_robusta_v1.0/GCF_000326865.1_Helobdella_robusta_v1.0_genomic.gbff.gz
11	Lottia gigantea<br>(Limpet)	Phylum: Mollusca<br>Class: Gastropoda<br>Order:Patellogastropoda<br>Family: Lottiidae<br>Genus: Lottia<br>Species: L. Gigantea	ftp://ftp.ncbi.nlm.nih.gov/genomes/all/GCF/000/327/385/GCF_000327385.1_Helro1/GCF_000327385.1_Helro1_genomic.gbff.gz
12	Daphnia pulex<br>(Water flea)	Phylum: Arthropoda<br>Class: Branchiopoda<br>Order: Cladocera<br>Family: Daphniidae<br>Genus: Daphnia<br>Species: D. Pulex	ftp://ftp.ncbi.nlm.nih.gov/genomes/all/GCA/000/187/875/GCA_000187875.1_V1.0/GCA_000187875.1_V1.0_genomic.gbff.gz
13	Anopheles gambiae<br>(Mosquito)	Phylum: Arthropoda<br>Class: Insecta<br>Order: Diptera	ftp://ftp.ncbi.nlm.nih.gov/genomes/all/GCF/000/005/575/GCF_000005575.2_AgamP3/GCF_0000

		Family: Culicidae<br>Genus: Anopheles<br>Species complex: A. gambiae	05575.2_AgamP3_genomic.gbff.gz
14	Caenorhabditis elegans<br>(Nematode worm)	Phylum: Nematoda<br>Class: Chromadorea<br>Order: Rhabditida<br>Family: Rhabditidae<br>Genus: Caenorhabditis<br>Species: C. Elegans	ftp://ftp.ncbi.nlm.nih.gov/genomes/all/GCF/000/002/985/GCF_000002985.6_WBcel235/GCF_000002985.6_WBcel235_genomic.gbff.gz
15	Schmidtea mediterranea<br>(Fresh-water flatworm)	Phylum: Platyhelminthes<br>Class: Rhabditophora<br>Order: Tricladida<br>Family: Dugesiidae<br>Genus: Schmidtea<br>Species: S. Mediterranea	ftp://ftp.ncbi.nlm.nih.gov/genomes/all/GCA/002/600/895/GCA_002600895.1_ASM260089v1/GCA_002600895.1_ASM260089v1_genomic.gbff.gz
16	Nematostella vectensis<br>(Sea anemone)	Phylum: Cnidaria<br>Class: Anthozoa<br>Order: Actiniaria<br>Family: Edwardsiidae<br>Genus: Nematostella<br>Species: N. Vectensis	ftp://ftp.ncbi.nlm.nih.gov/genomes/all/GCF/000/209/225/GCF_000209225.1_ASM20922v1/GCF_000209225.1_ASM20922v1_genomic.gbff.gz
17	Leptomonas pyrrhocoris<br>(Parasitic flagellate)	Phylum: Euglenozoa<br>Class: Kinetoplastea<br>Order: Trypanosomatida<br>Family: Trypanosomatidae<br>Genus: Leishmaniinae<br>Species: L. Leptomonas	ftp://ftp.ncbi.nlm.nih.gov/genomes/all/GCF/001/293/395/GCF_001293395.1_ASM129339v1/GCF_001293395.1_ASM129339v1_genomic.gbff.gz
18	Dictyostelium discoideum<br>(Slime mold)	Phylum: Amoebozoa<br>Class: Dictyostelia<br>Order: Dictyosteliida<br>Family: Dictyosteliidae<br>Genus: Dictyostelium<br>Species: D. discoideum	ftp://ftp.ncbi.nlm.nih.gov/genomes/all/GCF/000/004/695/GCF_000004695.1_dicty_2.7/GCF_000004695.1_dicty_2.7_genomic.gbff.gz
19	Tetrahymena thermophila<br>(Ciliates)	Phylum: Ciliophora<br>Class: Oligohymenophorea	ftp://ftp.ncbi.nlm.nih.gov/genomes/all/GCF/000/189/635/GCF_000189635.1_JCVI-TTA1-

		Order: Hymenostomatida<br>Family: Tetrahymenidae<br>Genus: Tetrahymena<br>Species: T. thermophila	2.2/GCF_000189635.1_JCVI-TTA1-2.2_genomic.gbff.gz
20	Xenopus tropicalis<br>(Tropical clawed frog)	Phylum: Chordata<br>Class: Amphibia<br>Order: Anura<br>Family: Pipidae<br>Genus: Xenopus<br>Species: X. Tropicalis	ftp://ftp.ncbi.nlm.nih.gov/genomes/all/GCF/000/004/195/GCF_000004195.3_Xenopus_tropicalis_v9.1/GCF_000004195.3_Xenopus_tropicalis_v9.1_genomic.gbff.gz
21	Amphimedon queenslandica<br>(Sponge)	Phylum: Porifera<br>Class: Demospongiae<br>Order: Haplosclerida<br>Family: Niphatidae<br>Genus: Amphimedon<br>Species: A. Queenslandica	ftp://ftp.ncbi.nlm.nih.gov/genomes/all/GCF/000/090/795/GCF_000090795.1_v1.0/GCF_000090795.1_v1.0_genomic.gbff.gz
22	Myotis brandtii<br>(Brandt's bat)	Phylum: Chordata<br>Class: Mammalia<br>Order: Chiroptera<br>Family: Vespertilionidae<br>Genus: Myotis<br>Species: M. Brandtii	ftp://ftp.ncbi.nlm.nih.gov/genomes/all/GCF/000/412/655/GCF_000412655.1_ASM41265v1/GCF_000412655.1_ASM41265v1_genomic.gbff.gz
23	Ophiophagus Hannah<br>(King cobra)	Phylum: Chordata<br>Class: Reptilia<br>Order: Squamata<br>Family: Elapidae<br>Genus: Ophiophagus<br>Species: O. Hannah	ftp://ftp.ncbi.nlm.nih.gov/genomes/all/GCA/000/516/915/GCA_000516915.1_OphHan1.0/GCA_000516915.1_OphHan1.0_genomic.gbff.gz
24	Sousa chinensis<br>(Indo-pacific humpbacked dolphin)	Phylum: Chordata<br>Class: Mammalia<br>Order: Artiodactyla<br>Family: Delphinidae<br>Genus: Sousa<br>Species: S. Chinensis	ftp://ftp.ncbi.nlm.nih.gov/genomes/all/GCF/000/442/215/GCF_000442215.1_Lipotes_vexillifer_v1/GCF_000442215.1_Lipotes_vexillifer_v1_genomic.gbff.gz
25	Ornithorhynchus anatinus<br>(Platypus)	Phylum: Chordata<br>Class: Mammalia<br>Order: Monotremata<br>Family: Ornithorhynchidae	ftp://ftp.ncbi.nlm.nih.gov/genomes/all/GCF/000/002/275/GCF_000002275.2_Ornithorhynchus_anatinus_5.0.1/GCF_000002275.2_Ornithorhynchus_anatinus_5.0.1

		Genus: Ornithorhynchus<br>Species: O. Anatinus	_genomic.gbff.gz
26	Alligator sinensis<br>(Chinese alligator)	Phylum: Chordata<br>Class: Reptilia<br>Order: Crocodilia<br>Family: Alligatoridae<br>Genus: Alligator<br>Species: A. Sinensis	ftp://ftp.ncbi.nlm.nih.gov/genomes/all/GCF/000/455/745/GCF_000455745.1_ASM45574v1/GCF_000455745.1_ASM45574v1_genomic.gbff.gz
27	Crocodylus porosus<br>(Saltwater crocodile)	Phylum: Chordata<br>Class: Reptilia<br>Order: Crocodilia<br>Family: Crocodylidae<br>Genus: Crocodylus<br>Species: C. Porosus	ftp://ftp.ncbi.nlm.nih.gov/genomes/all/GCF/001/723/895/GCF_001723895.1_CroPor_comp1/GCF_001723895.1_CroPor_comp1_genomic.gbff.gz

Table S2 Other related databases and their download sites

Genome database	Source	Links
Protozoan	NCBI	https://ftp.ncbi.nlm.nih.gov/refseq/release/protozoa/
Invertebrate	NCBI	https://ftp.ncbi.nlm.nih.gov/refseq/release/invertebrate/
Vertebrate_mammalian	NCBI	https://ftp.ncbi.nlm.nih.gov/refseq/release/vertebrate_mammalian/
Vertebrate_other	NCBI	https://ftp.ncbi.nlm.nih.gov/refseq/release/vertebrate_other/

Table S3 Motif basic information

Motif name	Representatives	species	Gene name	Genome location	Relation to the associated Gene	Possible function of the motif
EEF1A2-70236	105	77	*EEF1A2*	Chr20, NG_034083.1/7052-7123	intron	May cooperate with RBP (RNA binding proteins) to regulate gene

						expression
ZNF516-12356	120	102	*ZNF516*	Chr18, NG_032949.1/129,039-128,957	intron	May affect polyadenylation signal (PAS)
MYH7-3778	72	34	*MYH7*	Chr14, NG_007884.1/20350-20397	intron	May interact with RBP
ACVR2B-21548	33	18	*ACVR2B*	Chr3, NG_011791.1/29,424-29,498	intron	Not clear
ICE2-92051	20	11	*ICE2*	Chr15, NG_054881.1/6567-6643	intron	May interact with PAS
Motif-59232	55	43	None	Chr17, NG_030592.1/123,62-12,416	Intergenic region	Not clear
ANTXR2-33250	75	53	ANTXR2	Chr4, NG_015987.1/8484-8559	intron	Not clear
DPH1-85951	19	15	*DPH1*	Chr17， NG_051946.1/11073-11116	intron	May affect alternative splicing events
CD247-2015	42	31	*CD247*	Chr1, NG_007384.1/89,672-89,748	intron	May affect the RBP binding
ARNTL-1638	239	202	*ARNTL*	Chr11, NC_000011.10/13,387,129-13,387,264	3'UTR	Not clear
CAPN6-33096	79	60	Calpain 6 (CAPN6)	ChrX, NG_015962.1/27,810-27,900	intron	May affect PAS availability

Table S4 Primers and product sizes

Motif name	primer-F	primer-R	Product length
ZNF516-12356-Motif	GCCTACGCTGTGCTAAAAGG	GTCCATGTGCCATGTGGTTT	150bp
ZNF516-12356-Exon	TCTGCGGAGGTGGTATGAAC	GAGTGCGGAAAGAGCTTCCA	116bp
ARNTL-1638-Motif	GTGCACAGAAGCATCATTGGTAG	GATCATCCTTGAACACCCACTGTAG	313bp
ARNTL-1638-Exon	GTGAGAACCCCCACATAGGTATAGAC	TTACAGCGGCCATGGCAAG	161bp
NPTN-6924-Motif	GTGAATGAAGTAAACTTGAAGAAAAGTGTTAAC	CTTTTGGCCTGAGGGTTTGG	204bp
NPTN-6924-Exon	GTTTTTATCTTTGTGATTGTTGGTAGAGTTG	GAATTCTGGCTGAAATTATCATCCTTGTG	128bp
Beta-actin	GTACGTTGCTATCCAGGCTGTG	GGATCTTCATGAGGTAGTCAGTCAG	182 bp
