# Supplementary material for: Identification of 11 candidate structured noncoding RNA motifs in humans by comparative genomics
Source: BMC Genomics. 2021 Mar 9;22:164. doi: 10.1186/s12864-021-07474-9 (PMC7941889; doi:10.1186/s12864-021-07474-9)
Supplement: Supplementary file 2 — Additional file 2 Fig. S1. Comparison of covariations reported by R2R and R-scape. Fig. S2. Analysis of structured ncRNA candidates. [file 12864_2021_7474_MOESM2_ESM.pdf]

BY R-scape

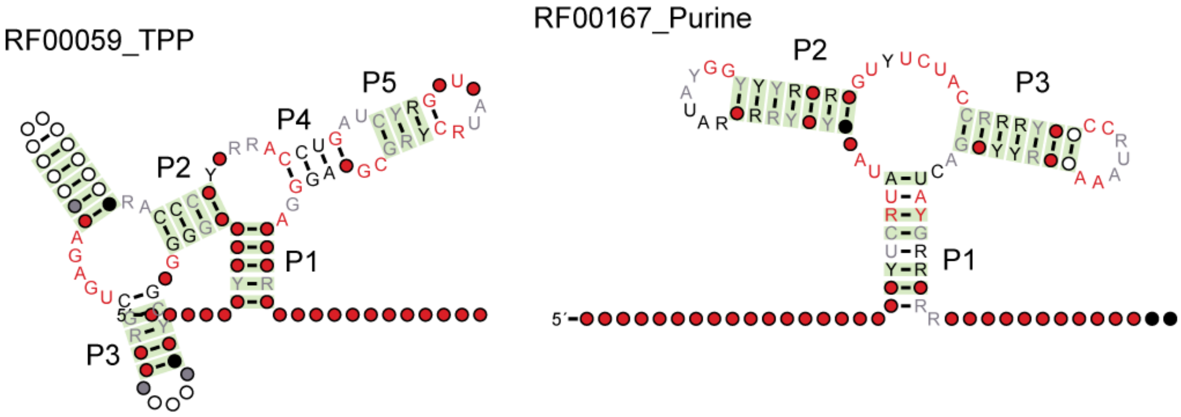

BY R2R

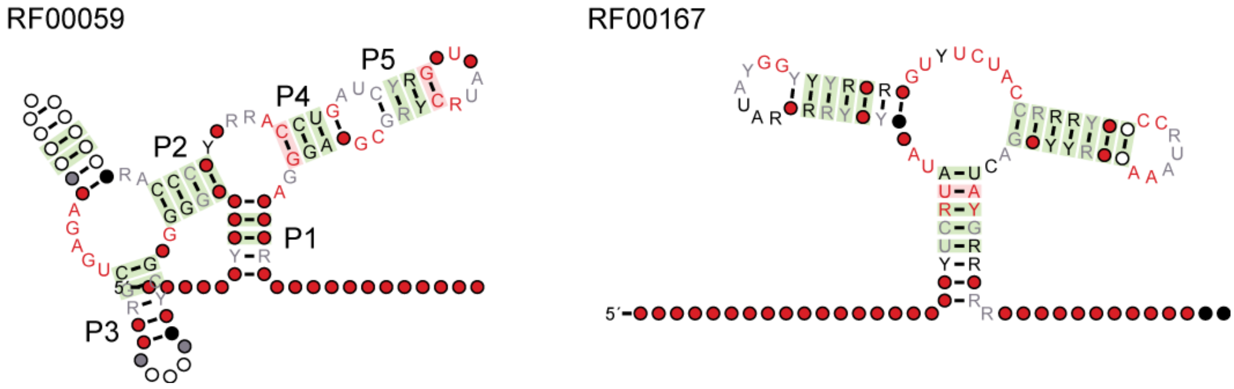

Fig. S1 Comparison of covariations reported by R2R (1) and R-scape (2). These figures were the consensus secondary structure produced by R2R and R-scape respectively based on the stockholm files downloaded from Rfam (3) for TPP riboswitch and purine riboswitch (RF00059_TPP (4) and RF00167_Purine (5)). P1 to P5 are the stems of the RNA structures and other annotations are as those listed in the Fig.1.

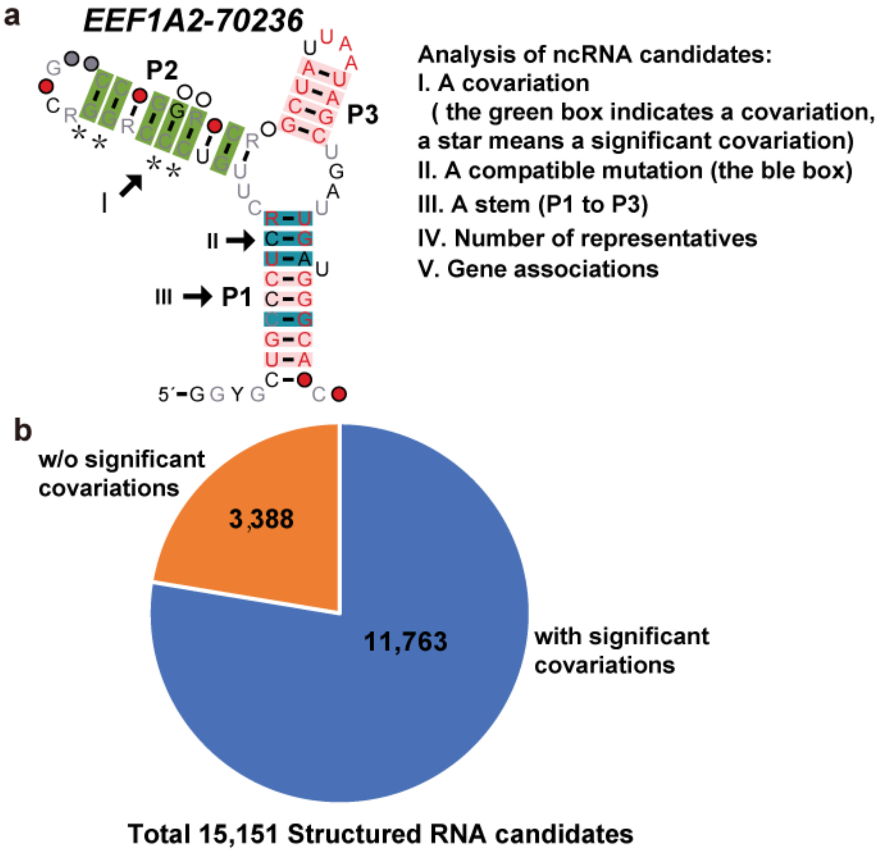

Fig. S2 Analysis of structured ncRNA candidates. **a** Potential candidates are evaluated by several criteria listed beside the figure. P1, P2, and P3 are stems of the motif, and other structure annotations are as described for Fig. 1. **b** Selection of candidate structured RNA motifs. The pie chart shows a total of 15,151 motifs detected by the CM-line. These motifs have been filtered by Rfam (3). When detected by R-scape, 11,763 motifs of the total contain significant covariations (Stockholm files are in the Additional file 19).

Reference

1. Weinberg, Z. and Breaker, R.R. (2011) R2R--software to speed the depiction of aesthetic consensus RNA secondary structures. *BMC bioinformatics*, **12**, 3.
2. Rivas, E., Clements, J. and Eddy, S.R. (2017) A statistical test for conserved RNA structure shows lack of evidence for structure in lncRNAs. *Nature methods*, **14**, 45-48.
3. Nawrocki, E.P., Burge, S.W., Bateman, A., Daub, J., Eberhardt, R.Y., Eddy, S.R., Floden, E.W., Gardner, P.P., Jones, T.A., Tate, J. *et al.* (2015) Rfam 12.0: updates to

the RNA families database. *Nucleic acids research*, **43**, D130-137.

4. Winkler, W., Nahvi, A. and Breaker, R.R. (2002) Thiamine derivatives bind messenger RNAs directly to regulate bacterial gene expression. *Nature*, **419**, 952-956.

5. Mandal, M., Boese, B., Barrick, J.E., Winkler, W.C. and Breaker, R.R. (2003) Riboswitches control fundamental biochemical pathways in Bacillus subtilis and other bacteria. *Cell*, **113**, 577-586.
