# Supplementary material for: Identification of 11 candidate structured noncoding RNA motifs in humans by comparative genomics
Source: BMC Genomics. 2021 Mar 9;22:164. doi: 10.1186/s12864-021-07474-9 (PMC7941889; doi:10.1186/s12864-021-07474-9)
Supplement: Supplementary file 14 — Additional file 14 Fig. S3. Genomic locus diagram. [file 12864_2021_7474_MOESM14_ESM.pdf]

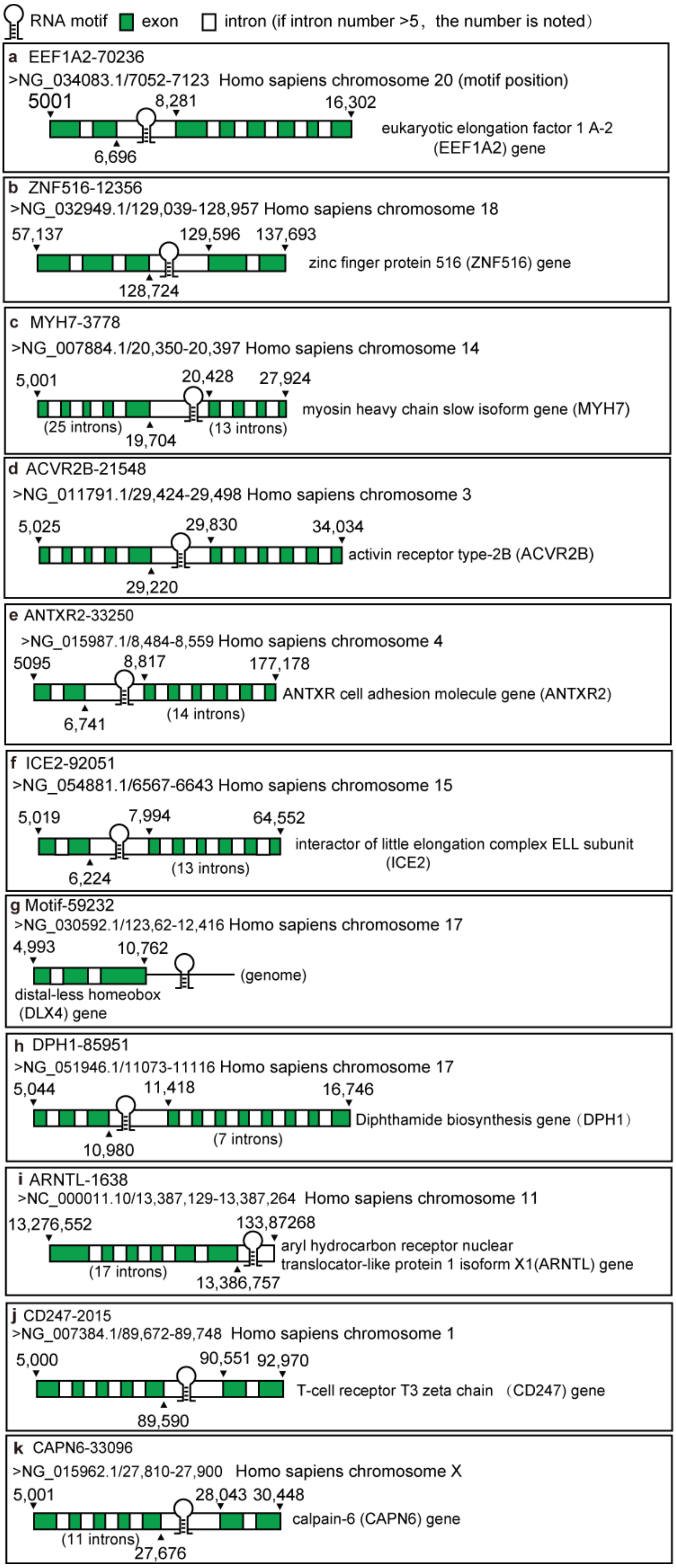
RNA motif
exon
intron (if intron number >5, the number is noted)
a EEF1A2-70236
>NG_034083.1/7052-7123 Homo sapiens chromosome 20 (motif position)
5001
8,281
16,302
6,696
eukaryotic elongation factor 1 A-2 (EEF1A2) gene
b ZNF516-12356
>NG_032949.1/129,039-128,957 Homo sapiens chromosome 18
57,137
129,596
137,693
128,724
zinc finger protein 516 (ZNF516) gene
c MYH7-3778
>NG_007884.1/20,350-20,397 Homo sapiens chromosome 14
5,001
20,428
27,924
(25 introns)
19,704
(13 introns)
myosin heavy chain slow isoform gene (MYH7)
d ACVR2B-21548
>NG_011791.1/29,424-29,498 Homo sapiens chromosome 3
5,025
29,830
34,034
29,220
activin receptor type-2B (ACVR2B)
e ANTXR2-33250
>NG_015987.1/8,484-8,559 Homo sapiens chromosome 4
5095
8,817
177,178
6,741
(14 introns)
ANTXR cell adhesion molecule gene (ANTXR2)
f ICE2-92051
>NG_054881.1/6567-6643 Homo sapiens chromosome 15
5,019
7,994
64,552
6,224
(13 introns)
interactor of little elongation complex ELL subunit (ICE2)
g Motif-59232
>NG_030592.1/123,62-12,416 Homo sapiens chromosome 17
4,993
10,762
(genome)
distal-less homeobox (DLX4) gene
h DPH1-85951
>NG_051946.1/11073-11116 Homo sapiens chromosome 17
5,044
11,418
16,746
10,980
(7 introns)
Diphthamide biosynthesis gene (DPH1)
i ARNTL-1638
>NC_000011.10/13,387,129-13,387,264 Homo sapiens chromosome 11
13,276,552
133,87268
(17 introns)
13,386,757
aryl hydrocarbon receptor nuclear translocator-like protein 1 isoform X1(ARNTL) gene
j CD247-2015
>NG_007384.1/89,672-89,748 Homo sapiens chromosome 1
5,000
90,551
92,970
89,590
T-cell receptor T3 zeta chain (CD247) gene
k CAPN6-33096
>NG_015962.1/27,810-27,900 Homo sapiens chromosome X
5,001
28,043
30,448
(11 introns)
27,676
calpain-6 (CAPN6) gene

Fig. S3 The location of 11 conserved motifs found in the human genome. a to k are diagrams showing motif locations on the genome. Usually, only one gene most closely related to the motif was shown in the diagram. The diagram is not scaled. The green boxes are exons and the white boxes are introns. If the number of introns is greater than five, the complete number of introns will not be drawn, but the number will be marked next to the bar. The contig number, motif position and chromosome number were provided in the figure. The numbers on the bar show the beginning and the end of the gene. Only the splicing sites adjacent to the motif are labeled. The genes that are associated with these motifs are listed alongside. Since we can only organize the information manually so far, diagrams for other species are not shown here. Please find location information about motifs in other species in Pubmed (https://www.ncbi.nlm.nih.gov/nuccore/). by using the contig number in the Infernal result (in additional_file_2) (such as NG_015962.1 / 27,810-27,900).
