# Supplementary material for: Identification of 11 candidate structured noncoding RNA motifs in humans by comparative genomics
Source: BMC Genomics. 2021 Mar 9;22:164. doi: 10.1186/s12864-021-07474-9 (PMC7941889; doi:10.1186/s12864-021-07474-9)
Supplement: Supplementary file 15 — Additional file 15 Fig. S4. The full-length original agarose gels for Fig. 5. [file 12864_2021_7474_MOESM15_ESM.pdf]

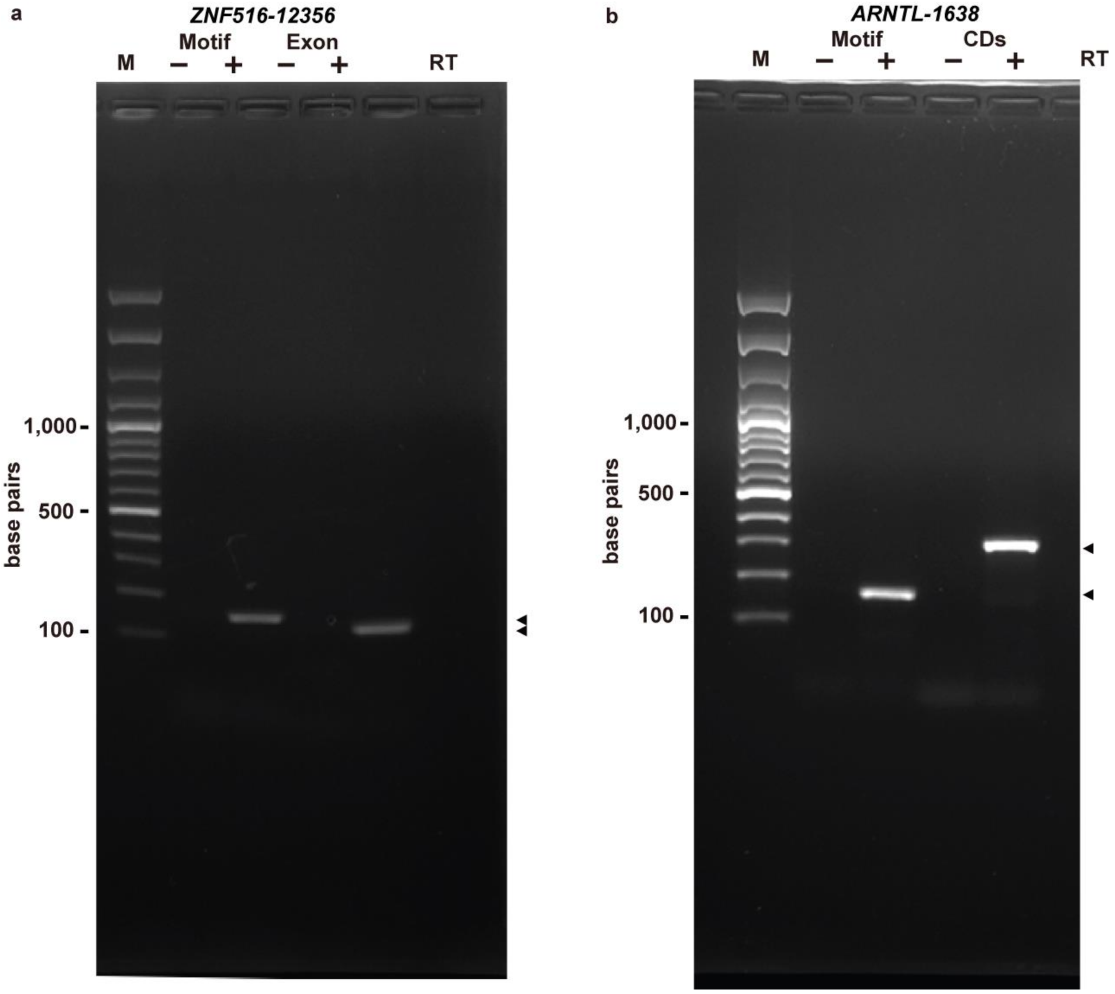

Fig. S4 The full-length original agarose gels for Fig. 5. a Agarose gel separation of RT-PCR products of *ZNF516-12356* motif, generated by using primers for the RNA motif itself (Motif) and mRNA coding regions (Exon). M stands for DNA markers. Lanes containing PCR products are marked with (+) or (-), indicating the presence or absence of reverse transcriptase (RT). The two arrows next to the gel indicate bands corresponding to DNA products of the expected size. **b** Agarose gel separation of RT-PCR products of *ARNTL-1638* motif. Gel annotations are as described for a. The gel pictures in this figure are corresponding to the gels in Fig. 5 b and d.
