# Supplementary material for: Identification of 11 candidate structured noncoding RNA motifs in humans by comparative genomics
Source: BMC Genomics. 2021 Mar 9;22:164. doi: 10.1186/s12864-021-07474-9 (PMC7941889; doi:10.1186/s12864-021-07474-9)
Supplement: Supplementary file 16 — Additional file 16 Fig. S5. Real-time PCR for the NPTN-6924 motif. [file 12864_2021_7474_MOESM16_ESM.pdf]

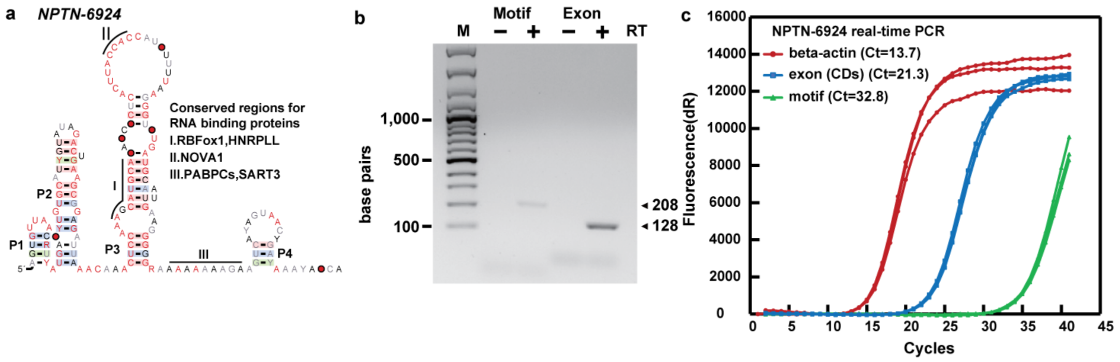


Fig. S5 The gene expression of the *NPTN-6924* RNA candidate. It is one of the motifs discovered in the CM-line process (but it is not one of the 11 motifs described in the main text). The motif is located in the 7th intron region of the *NPTN* gene, which encodes a type I transmembrane protein belonging to the immunoglobulins (Ig) superfamily (1). a. Consensus sequence and secondary structure of the NPTN-6924 RNA candidate. b. Agarose gel separation of RT-PCR products of *NPTN-6924* motif, generated by using primers for the RNA motif itself (Motif) and mRNA coding regions (Exon). The full-length of the original gel is in the Fig. S6 (in the Additional file 25). c. Real-time PCR results were performed using primers for the RNA motif (in the intron) and the exon containing the RNA motif (β-actin RNA was used as a control for constitutively expressed genes). The experiment was repeated twice and the results were similar.

Reference

1. Saito A, Fujikura-Ouchi Y, Kuramasu A, Shimoda K, Akiyama K, Matsuoka H, Ito C. 2007. Association study of putative promoter polymorphisms in the neuroplastin gene and schizophrenia. *Neurosci Lett* **411**: 168-173.
