# Supplementary material for: Identification of 11 candidate structured noncoding RNA motifs in humans by comparative genomics
Source: BMC Genomics. 2021 Mar 9;22:164. doi: 10.1186/s12864-021-07474-9 (PMC7941889; doi:10.1186/s12864-021-07474-9)
Supplement: Supplementary file 17 — Additional file 17. Pictures of sequence alignments for 11 RNA motifs. [file 12864_2021_7474_MOESM17_ESM.pdf]

This file contains the consensus secondary structures and sequence alignments for 11 ncRNA candidates listed in the main text. Their original stockholm files for each motif are in the additional_file_2. The following sequence alignments were produced by using Emacs to convert the color Stockholm format to the postscript format, and then using Adobe Acrobat to convert the postscript to PDF.

a *EEF1A2-70236*

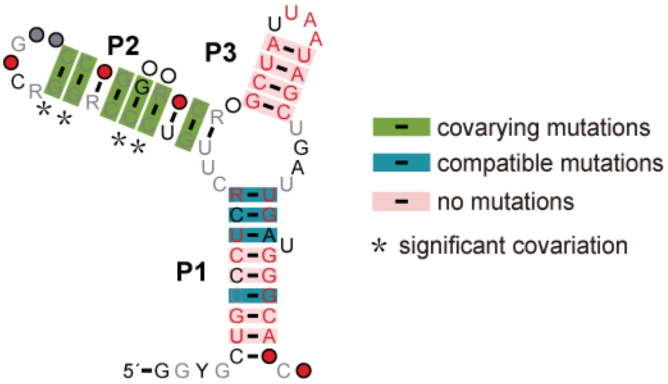

Sequence alignment for the motif *EEF1A2-70236*

```
# STOCKHOLM 1.0
NW_005371769.1/656376-656447          GGCACUGCCCUCGCUUGUUCC....AGG..GCUGGGCCAGG......A.cUCA--g....ggUGCUAU...UAAUAGCUGAUUGAUGGGCACUG
NW_005871603.1/5704-5633              GGCACUGCCCUCGCUUGUUCC....AGG..GCUGGGCCAGG......A.cUCA--g....ggUGCUAU...UAAUAGCUGAUUGAUGGGCACUG
NW_019160913.1/579056-579128          GGCGCUGUCCUCGCUUGUCCC....AGG..GCUGGGCCAGGac.ucaG.aGCA--........-GCUAU...UAAUAGCUGAUUGAUGGGCACCC
NW_019873576.1/28864600-28864528      GGCGCUGUCCUCGCUUGUCCC....AGG..GCUGGGCCAGGac.ucaG.aGCA--........-GCUAU...UAAUAGCUGAUUGAUGGGCACCC
NW_020173687.1/575354-575426          GGCGCUGUCCUCGCUUGUCCC....AGG..GCUGGGCCAGGac.ucaG.aGCA--........-GCUAU...UAAUAGCUGAUUGAUGGGCACCC
NW_006789032.1/608036-608108          GGCGCUGUCCUCGCUUGUCCC....AGG..GCUGGGCCAGGac.ucaG.aGCA--........-GCUAU...UAAUAGCUGAUUGAUGGGCACCC
NW_007370808.1/2286040-2285970        GGCACUGCCCUCGCUUGUUCC....AGG..GCUGGGCCAGG......A.cGCAGG.......UGCUAU...UAAUAGCUGGUUGGUGGGCACCC
NC_000020.11/63497264-63497193        GGUACUGCCCUCACUUGUCCUa..gGGC..CCU---GCCAG......G..GCACA.......CGCUACuauUAAUAGCUGAUUGAUGGGCAGCC
NT_011362.11/32445756-32445685        GGUACUGCCCUCACUUGUCCUa..gGGC..CCU---GCCAG......G..GCACA.......CGCUACuauUAAUAGCUGAUUGAUGGGCAGCC
NG_034083.1/7052-7123                 GGUACUGCCCUCACUUGUCCUa..gGGC..CCU---GCCAG......G..GCACA.......CGCUACuauUAAUAGCUGAUUGAUGGGCAGCC
NW_006294993.1/612878-612949          GGCACUGCCCUCACUUGUUCC....AGG..GCUGGGCCAAG......A.cUCA--g....ggUGCUAU...UAAUAGCUGAUUGAUGGGCACUG
NW_004955528.1/719234-719306          GACGAUGCCCUCACUUGUCCC....AGG..GCUGGGCCAGG......G.cUCA--ug...gcUGCUAU...UAAUAGCUGAUUGAUGGGCAGCA
NW_004454186.1/711450-711522          GGCGCUGCCCUCGCUUGUCCC....AGG..GCUGGGCCAGGacucaaG..GCG--........-GCUAU...UAAUAGCGGAUUGAUGGGCACCG
NW_007907089.1/625286-625358          GGUGCUGCCCUCACUUGUCCC....AGG..GCUGGGCCAGGacucagG..GUG--........-GCUAU...UAAUAGCUGAUUGAUGGGCACCC
NT_176300.1/842740-842812             GACGCUGCCCUCACUUGUCCC....AGG..GCUGGGCCAGG......G.cUCA--ug...gcUGCUAU...UAAUAGCUGAUUGAUGGACAGCU
NW_018500483.1/2004693-2004622        GGUGCUGCCCUCACUUGUCCC....GGG..GCUGUGCCAGGa....cG.cACG--.......cUGCUAU...UAAUAGCUGAUUGAUGGGCAGCC
NC_036899.1/63215353-63215282         GGUACUGCCCUCACUUGUCCUa..gGGC..CCU---GCCAG......G..GCGCA.......CGCUACuauUAAUAGCUGAUUGAUGGGCAGCC
NW_014014390.1/307631-307560          GGUACUGCCCUCACUUGUCCUa..gGGC..CCU---GCCAG......G..GCGCA.......CGCUACuauUAAUAGCUGAUUGAUGGGCAGCC
NC_027888.1/61421237-61421166         GGUACUGCCCUCACUUGUCCUa..gGGC..CCU---GCCAG......G..GCGCA.......CGCUACuauUAAUAGCUGAUUGAUGGGCAGCC
NW_017493714.1/32919414-32919343      GGUACUGCCCUCACUUGUCCUa..gGGC..CCU---GCCAG......G..GCGCA.......CGCUACuauUAAUAGCUGAUUGAUGGGCAGCC
NC_036923.1/60084330-60084259         GGUACUGCCCUCACUUGUCCUa..gGGC..CCU---GCCAG......G..GCGCA.......CGCUACuauUAAUAGCUGAUUGAUGGGCAGCC
NW_019937254.1/757530-757601          GGUACUGCCCUCACUUGUCCUa..gGGC..CCU---GCCAG......G..GCGCA.......CGCUACuauUAAUAGCUGAUUGAUGGGCAGCC
NC_018444.2/62451940-62451869         GGUACUGCCCUCACUUGUCCUa..gGGC..CCU---GCCAG......G..GCGCA.......CGCUACuauUAAUAGCUGAUUGAUGGGCAGCC
NW_019932871.1/820080-820151          GGUACUGCCCUCACUUGUCCUa..gGGC..CCU---GCCAG......G..GCGCA.......CGCUACuauUAAUAGCUGAUUGAUGGGCAGCC
NW_018084702.1/47496466-47496394      GGCACUGUCCUCGCUUGUCCC....AGG..GCUGGGCCAGGacucagG..GUG--........-GCUAU...UAAUAGCUGAUUGAUGGGCACCC
NC_010459.5/62524266-62524194         GGCACUGUCCUCGCUUGUCCC....AGG..GCUGGGCCAGGacucagG..GUG--........-GCUAU...UAAUAGCUGAUUGAUGGGCACCC
NW_004524770.1/855003-855075          GACGCUGCCCUCACUUGUCCC....AGG..GAUGGGCCAGG......G.cUCG--ug...gcUGCUAU...UAAUAGCUGAUUGAUGGGCAGCU
NW_003726101.1/47103965-47103893      GGUGAUGCCCUCACUUGUCCC....AGG..GCUGGGCCAGGacucagG..GCG--........-GCUAU...UAAUAGCUGAUUGAUGGGCACCC
NW_020356543.1/595025-595097          GGUGAUGCCCUCACUUGUCCC....AGG..GCUGGGCCAGGacucagG..GCG--........-GCUAU...UAAUAGCUGAUUGAUGGGCACCC
NC_006606.3/47104065-47103993         GGUGAUGCCCUCACUUGUCCC....AGG..GCUGGGCCAGGacucagG..GCG--........-GCUAU...UAAUAGCUGAUUGAUGGGCACCC
NW_020269161.1/47401214-47401142      GGUGAUGCCCUCACUUGUCCC....AGG..GCUGGGCCAGGacucagG..GCG--........-GCUAU...UAAUAGCUGAUUGAUGGGCACCC
NC_023643.1/714089-714160             GGUGCUGCCCUCACUCGUCCC....AGGgcCCUG--CCAGGa....cG.cACGC-.......UGCUAU...UAAUAGCUGAUUGAUGGGCAGCC
NW_006735235.1/714089-714160          GGUGCUGCCCUCACUCGUCCC....AGGgcCCUG--CCAGGa....cG.cACGC-.......UGCUAU...UAAUAGCUGAUUGAUGGGCAGCC
NW_016107870.1/448397-448326          GGUGCUGCCCUCACUUGUCCC....AGGgcCCUG--CCAGG......A.cACACA......aUGCUAU...UAAUAGCUGAUUGAUGGGCAGCC
NW_004438579.1/6205483-6205411        GGCGCUGUCCUCGCUUGUCCC....GGG..GCUGGGCCAGGac.ucaG.aGCG--........-GCUAU...UAAUAGCUGAUUGAUGGGCACCC
NW_017739293.1/507825-507897          GGCACUGCCCUCACUUGUCCC....AGG..GCUGAGCCAGGacucagG..GCA--........-GCUAU...UAAUAGCUGCUUGAUGGGCACCC
NW_004624741.1/29161891-29161819      GACGCUGCCCUCACUUGUCCC....AGG..GCUGGGCCAGG......G.cUCA--ug...gcUGCUAU...UAAUAGAUGAUUGAUGGGCAGCU
NW_011043433.1/2991635-2991563        GACGCUGCCCUCACUUGUCCC....AGG..GCUGGGCCAGG......G.cUCA--ug...gcUGCUAU...UAAUAGAUGAUUGAUGGGCAGCU
NW_020251514.1/39215480-39215551      GGUGCUGCCCUCACUUGUCCC....AGGgcCCUG--CCAGGa....cG.cACG--.......cUGCUAU...UAAUAGCUGAUUGAUGGGCAGCC
NC_022281.1/95692636-95692565         GGUGCUGCCCUCACUUGUCCC....AGGgcCCUG--CCAGGa....cG.cACG--.......cUGCUAU...UAAUAGCUGAUUGAUGGGCAGCC
NW_012104365.1/5549515-5549444        GGUGCUGCCCUCACUUGUCCC....AGGgcCCUG--CCAGGa....cG.cACG--.......cUGCUAU...UAAUAGCUGAUUGAUGGGCAGCC
NW_018757282.1/131311-131382          GGUGCUGCCCUCACUUGUCCC....AGGgcCCUG--CCAGGa....cG.cACG--.......cUGCUAU...UAAUAGCUGAUUGAUGGGCAGCC
NW_003943655.1/15597451-15597380      GGUGCUGCCCUCACUUGUCCC....AGGgcCCUG--CCAGGa....cG.cACG--.......cUGCUAU...UAAUAGCUGAUUGAUGGGCAGCC
NW_014805213.1/360520-360591          GGUGCUGCCCUCACUUGUCCC....AGGgcCCUG--CCAGGa....cG.cACG--.......cUGCUAU...UAAUAGCUGAUUGAUGGGCAGCC
NW_012014244.1/837823-837894          GGUGCUGCCCUCACUUGUCCC....AGGgcCCUG--CCAGGa....cG.cACG--.......cUGCUAU...UAAUAGCUGAUUGAUGGGCAGCC
NC_018161.2/776714-776785             GGUGCUGCCCUCACUUGUCCC....AGGgcCCUG--CCAGGa....cG.cACG--.......cUGCUAU...UAAUAGCUGAUUGAUGGGCAGCC
NW_005092979.1/59933173-59933102      GGUGCUGCCCUCACUUGUCCC....AGGgcCCUG--CCAGGa....cG.cACG--.......cUGCUAU...UAAUAGCUGAUUGAUGGGCAGCC
NC_027902.1/800561-800632             GGUGCUGCCCUCACUUGUCCC....AGGgcCCUG--CCAGGa....cG.cACG--.......cUGCUAU...UAAUAGCUGAUUGAUGGGCAGCC
NC_037678.1/39215480-39215551         GGUGCUGCCCUCACUUGUCCC....AGGgcCCUG--CCAGGa....cG.cACG--.......cUGCUAU...UAAUAGCUGAUUGAUGGGCAGCC
NW_017731382.1/563347-563419          GGCACUGCCCUCACUUGUCCC....AGG..GCUAGGCCAGGacucagG..GCA--........-GCUAU...UAAUAGCUGCUUGAUGGGCACCC
NW_012114444.1/1047573-1047644        GGUGCUGCCCUCACUUGUCCC....AGGgcCCUG--CCAGGa....cG.cACG--.......cUGCUAU...UAAUAGCUGAUUGAUGGGCAGCU
NW_019318073.1/2144512-2144441        GGUGCUGCCCUCACUUGUCCC....AGGgcCCUG--CCAGGa....cG.cACG--.......cUGCUAU...UAAUAGCUGAUUGAUGGGCAGCU
NW_010803524.1/6986620-6986549        GGUGCUGCCCUCACUUGUCCC....AGGgcCCUG--CCAGGa....cG.cACG--.......cUGCUAU...UAAUAGCUGAUUGAUGGGCAGCU
NW_016807321.1/1661415-1661344        GGUGCUGCCCUCACUUGUCCC....AGGgcCCUG--CCAGGa....cG.cACG--.......cUGCUAU...UAAUAGCUGAUUGAUGGGCAGCU
NW_012001373.1/792368-792439          GGUGCUGCCCUCACUUGUCCC....AGGgcCCUG--CCAGGa....cG.cACG--.......cUGCUAU...UAAUAGCUGAUUGAUGGGCAGCU
NW_020313504.1/647910-647982          GGUGCUGCCCUUACUUGUCCC....AGG..GCUGGGCCAGGacucagG..GUG--........-GCUAU...UAAUAGCUGAUUGAUGGGCAGCC
NW_004450733.1/653485-653557          GGUGCUGCCCUUACUUGUCCC....AGG..GCUGGGCCAGGacucagG..GUG--........-GCUAU...UAAUAGCUGAUUGAUGGGCAGCC
NC_009165.3/50233218-50233146         GGCGCUGCCCUCACUUGUCCC....AGG..GCUGGGCCAGGacucagG..GCG--........-GCUAU...UAAUAGCGGACUGAUGGGCACGG
NW_007678148.1/71857-71785            GGCGCUGCCCUCACUUGUCCC....AGG..GCUGGGCCAGGacucagG..GCG--........-GCUAU...UAAUAGCGGACUGAUGGGCACGG
NW_019641733.1/50233218-50233146      GGCGCUGCCCUCACUUGUCCC....AGG..GCUGGGCCAGGacucagG..GCG--........-GCUAU...UAAUAGCGGACUGAUGGGCACGG
NW_015503968.1/598724-598796          GGCACUGCCCUCACUUGUUCC....AGG..GCUGGGCGAGG......A.cUCA--gg...guGGCUAU...UAAUAGCUGAUUGAUGGGCACUG
NW_004087898.1/784862-784933          GGUACUGCCCUCACUUGUCCC....AGGgcCCUG--CCAGGa....cG.cACG--.......cUGCUAU...UAAUAGCUGAUUGAUGGGCAGUC
NC_019828.1/785862-785933             GGUACUGCCCUCACUUGUCCC....AGGgcCCUG--CCAGGa....cG.cACG--.......cUGCUAU...UAAUAGCUGAUUGAUGGGCAGUC
NC_034571.1/170693365-170693295       GGUGCUGUCCUCG--CUUGUC....UGGcaACAG--CCAGGg...cuC..AAGGA.......UGCUAU...UAAUAGCUGAUUGAUGGGCAGCA
NT_039212.7/3440915-3440845           GGUGCUGUCCUCG--CUUGUC....UGGcaACAG--CCAGGg...cuC..AAGGA.......UGCUAU...UAAUAGCUGAUUGAUGGGCAGCA


NW_018388414.1/170693365-170693295    GGUGCUGUCCUCG--CUUGUC....UGGcaACAG--CCAGGg...cuC..AAGGA.......UGCUAU...UAAUAGCUGAUUGAUGGGCAGCA
NC_000068.7/181155002-181154932       GGUGCUGUCCUCG--CUUGUC....UGGcaACAG--CCAGGg...cuC..AAGGA.......UGCUAU...UAAUAGCUGAUUGAUGGGCAGCA
NC_013900.1/57482058-57481987         GGUGCUGCCCUCACUUGUCCC....AGGgcCCUG--CCAGGa....cG.cACA--.......cUGCUAU...UAAUAGCUGAGUGAUGGGCAGCC
NW_003183460.1/866857-866786          GGUGCUGCCCUCACUUGUCCC....AGGgcCCUG--CCAGGa....cG.cACA--.......cUGCUAU...UAAUAGCUGAGUGAUGGGCAGCC
NW_006728129.1/602461-602533          GGCGCUGUCCUUGCUUGUCCC....AGG..GCUGGGCCAGG......A.cUCA--ga...guGGCUAU...UAAUAGCUGAUUGAUGGGCACCC
NW_003218269.1/161665-161593          GGUGCUGCCCUCACUUGUCCC....AGG..GCUGGGCCAGG......A.cUCA--gg...guGACUAU...UAAUAGCUGAUUGAUGGGCACCC
NW_018657759.1/702426-702356          GGUGCUGCCCUCA--CUUGUC....UUGcaACAG-CCAGGGc....uC..AAGGA.......UGCUAU...UAAUAGCUGAUUGAUGGGCAGCA
NW_018657759.1/2120695-2120765        GGUGCUGCCCUCA--CUUGUC....UUGcaACAG-CCAGGGc....uC..AAGGA.......UGCUAU...UAAUAGCUGAUUGAUGGGCAGCA
NW_006501809.1/785500-785570          GGUGCUGCCCUCA--CUUGCC....UUGcaACAG-CCAGGGc....uC..AAGGA.......UGCUAU...UAAUAGCCGAUUGAUGGGCAGCA
NW_018339209.1/524393-524465          GGCGCUGUCCUCGCUUGUCCC....AGG..GUAGGGCCAGG......G.cUCA--ga...guGGCUAU...UAAUAGCCAAUUGAUGGGCACCC
NW_008346900.1/2750426-2750356        GGUGCUGCCCUCACUUGUCUUccagCAG..CCAGGGCUCAG......G..GCU--........-GCUAU...UAAUAGCUGAUUGAUGGGCAGCC
NC_034592.1/163435545-163435475       UGUGCUGCCCUCA--CUUGUC....UGGcaAUA--GCCGGGg...cuC..AAGGA.......UGCUAU...UAAUAGCUGAUUGAUGGGCAGCA
NW_018391577.1/163435545-163435475    UGUGCUGCCCUCA--CUUGUC....UGGcaAUA--GCCGGGg...cuC..AAGGA.......UGCUAU...UAAUAGCUGAUUGAUGGGCAGCA
NW_006870187.1/811414-811344          GGUGUUGCCCUCA--CUUGUC....UUGcaACAG-CCAGGGc....uC..AAGGA.......UGCUAU...UAAUAGCUGAUUGAUGGGCAGCA
NW_003613943.1/1126537-1126467        GGUGUUGCCCUCA--CUUGUC....UUGcaACAG-CCAGGGc....uC..AAGGA.......UGCUAU...UAAUAGCUGAUUGAUGGGCAGCA
NW_006383949.1/137500-137572          GGUGUUGCCCUUACUUGUCCC....AGG..GCUGGGCCAGGacucagG..GUG--........-GCUAU...UAAUAGCUGACUGAUGGGCAGCC
NW_006804161.1/1063610-1063531        GGUGCUGCCCUCACUUGUCCC....AGG..GCUGGGCCUGGaccagaG..ACAC-ag..gguAGCUAU...UAAUAGCAGUAUGAUGGGCACCC
NW_020228696.1/29461960-29461888      GGCGCUGUCCUCGCUUGUCCC....AGG..GCAGGGCCAGG......A.cUCA--ga...guGGCUAU...UAAUAGCCAAUUGAUGGGCACUC
NC_030820.1/53477801-53477873         GGCGCUGUCCUCGCUUGUCCC....AGG..GCAGGGCCAGG......A.cUCA--ga...guGGCUAU...UAAUAGCCAAUUGAUGGGCACUC
NW_017189499.1/53477801-53477873      GGCGCUGUCCUCGCUUGUCCC....AGG..GCAGGGCCAGG......A.cUCA--ga...guGGCUAU...UAAUAGCCAAUUGAUGGGCACUC
NC_037558.1/29461960-29461888         GGCGCUGUCCUCGCUUGUCCC....AGG..GCAGGGCCAGG......A.cUCA--ga...guGGCUAU...UAAUAGCCAAUUGAUGGGCACUC
NW_014639022.1/53400317-53400389      GGUGCUGUCCUCGCUUGUCCC....AGG..GCAGGGCCAGG......A.cUCA--ga...guGGCUAU...UAAUAGCCAAUUGAUGGGCACUC
NC_019470.2/53400317-53400389         GGUGCUGUCCUCGCUUGUCCC....AGG..GCAGGGCCAGG......A.cUCA--ga...guGGCUAU...UAAUAGCCAAUUGAUGGGCACUC
NW_011942240.1/6597695-6597623        GGUGCUGUCCUCGCUUGUCCC....AGG..GCAGGGCCAGG......A.cUCA--ga...guGGCUAU...UAAUAGCCAAUUGAUGGGCACUC
NW_003573541.1/706688-706756          GGGGCUGCCCUCACUUGACAG....GGC..ACAAGGGCUCUc....uG..GCU--........-GCUAU...UAAUAGCUGAUUGAUGGGCAGCG
NW_014638014.1/691916-691988          GGCGCUGCCCUCACUUGUCCC....AGG..GCUGGGCCAGGacucagG..GUG--........-GCUAU...UAAUAGCGGACUGAUGGGCAUGG
NW_016530862.1/149166-149094          GUCACUGCCCUCCCUUGUCCC....GGG..GCUGGGCCGGGacuuagG..GCG--........-GCUAU...UAAUAGCUGAGUGAUGGGCACCC
NW_004569216.1/8333882-8333810        GAUGCUGCCCUCACUUGUCCU....GGG..GCCGGGCCAGGccucagG..GUG--........-GCUAU...UAAUAGCUGACUGAUGGGCACUU
NW_017871285.1/14354-14426            GGUGCUGUGCUCACUUGUCCC....AGA..GCUGAGCCAGG......G.cUCA--gg...gcUGCUAU...UAAUAGCUGAUUGAUGGGCAGCC
NW_004801651.1/12300877-12300807      GGUGCUGCCCUCA--CUUGCC....CUGcaACA-GCCAGGGc....uC..AAGGA.......UGCUAU...UAAUAGCUGAUUGACAGGCAGCA
NW_015351210.1/780432-780503          GGUGCUGCCCUCGGUUGUCCC....AGG..AUUGGG-CAGGacucagG..GCU--........-GCUAU...UAAUAGCUGAUUGACGGGCAGUG
NW_004936514.1/10805206-10805135      GGUGCUGCCCUCGGUUGUCCC....AGG..AUUGGG-CAGGacucagG..GCU--........-GCUAU...UAAUAGCUGAUUGACGGGCAGUG
NW_019154090.1/611346-611418          GAUGCUGCCCUCGCUUGUCCU....GGG..GCUGG-CCCAG......GccUCA--gg...guGGCUAU...UAAUAGCUGACUGGUGGGCACUC
NW_018734260.1/630291-630363          GGUGGUGCCCUUACUUGUCCC....AGG..GCUGGGCCAGGacucagG..GUG--........-GCUAU...UAAUAGCUGACUGAUGGGCAGCC
NW_005398174.1/82762-82690            GGCGCUGUCCUCGCUUGUCCC....AGG..GCAGGGUCAGG......A.cUCA--ga...guGGCUAU...UAAUAGCCAAUUGAUGGGCACUC
NC_032662.1/54836724-54836796         GGCGCUGUCCUCGCUUGUCCC....AGG..GCAGGGUCAGG......A.cUCA--ga...guGGCUAU...UAAUAGCCAAUUGAUGGGCACUC
NW_020190097.1/54153900-54153972      GGCGCUGUCCUCGCUUGUCCC....AGG..GCAGGGUCAGG......A.cUCA--ga...guGGCUAU...UAAUAGCCAAUUGAUGGGCACUC
NW_017844721.1/54836724-54836796      GGCGCUGUCCUCGCUUGUCCC....AGG..GCAGGGUCAGG......A.cUCA--ga...guGGCUAU...UAAUAGCCAAUUGAUGGGCACUC
NW_011494635.1/15761002-15761074      GGCGCUGUCCUCGCUUGUCCC....AGG..GCAGGGUCAGG......A.cUCA--ga...guGGCUAU...UAAUAGCCAAUUGAUGGGCACUC
NC_037340.1/54153900-54153972         GGCGCUGUCCUCGCUUGUCCC....AGG..GCAGGGUCAGG......A.cUCA--ga...guGGCUAU...UAAUAGCCAAUUGAUGGGCACUC
#=GC SS_cons                          ::::((((((((,,<<<<<<....<<<..______>>>>>......>..>>>,,........,<<<<_...___>>>>,,,,)))-))))))::
#=GC RF                               GGcGcugCCCUCgcUuGggcc....cGg..aCUGGgcCggg......c..cCacA.......uGCUau...UAAuAGCUGAUuGAUGGGcagcg
//
```

b *ZNF516-12356*

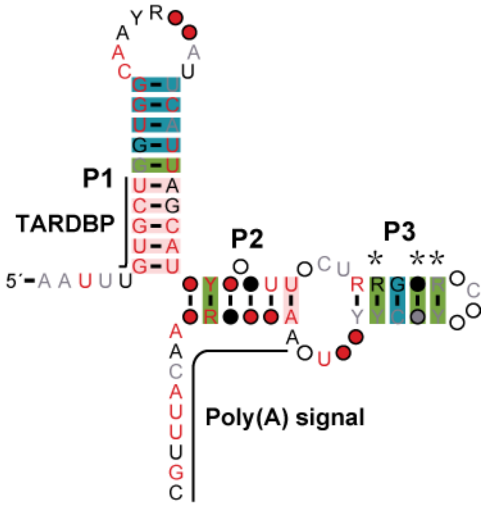

Sequence alignment for the motif *ZNF516-12356*

```
# STOCKHOLM 1.0
NW_009912094.1/42592-42672         AAUUUGUGCUGGUGGCAACAGAAUUCAUUAGCA..UAUUCAUUUCU.GAG.UA-CCC-----.........UAC.UC.U.CUUAAUUa..aAAU..AAa.aCAUUUGC
NW_009752531.1/20242-20163         AAUUUGUGCUGGUGGCAACAGAAUUCAUUAGCA..UAUUCAUUUCU.GAG.UA-CCC-----.........UAC.UC.U.CU-AAUUa..aAAU..AAa.aCAUUUGC
NW_014650526.1/3948838-3948759     AAUUUGUGCUGGUGGCAACAGAAUUCAUUAGCA..UAUUCAUUUCU.GAG.UA-CCC-----.........UAC.UC.U.CU-AAUUa..aAAU..AAa.aCAUUUGC
NW_013185697.1/4777134-4777055     AAUUUGUGCUGGUGGCAACAGAAUUCAUUAGCA..UAUUCAUUUCU.GAG.UA-CCC-----.........UAC.UC.U.CU-AAUUa..aAAU..AAa.aCAUUUGC
NW_009952485.1/1560575-1560654     AAUUUGUGCUGGUGGCAACAGAAUUCAUUAGCA..UAUUCAUUUCU.GAG.UA-CCC-----.........UAC.UC.U.CU-AAUUa..aAAU..AAa.aCAUUUGC
NC_031769.1/92709627-92709548      AAUUUGUGCUGGUGGCAACAGAAUUCAUUAGCA..UAUUCAUUUCU.GAG.UA-CCC-----.........UAC.UC.U.CU-AAUUa..aAAU..AAa.aCAUUUGC
NW_018362566.1/4792215-4792136     AAUUUGUGCUGGUGGCAACAGAAUUCAUUAGCA..UAUUCAUUUCU.GAG.UA-CCC-----.........UAC.UC.U.CU-AAUUa..aAAU..AAa.aCAUUUGC
NC_021673.1/95982929-95982850      AAUUUGUGCUGGUGGCAACAGAAUUCAUUAGCA..UAUUCAUUUCU.GAG.UA-CCC-----.........UAC.UC.U.CU-AAUUa..aAAU..AAa.aCAUUUGC
NW_009702074.1/21872-21793         AAUUUGUGCUGGUGGCAACAGAAUUCAUUAGCA..UAUUCAUUUCU.GAG.UA-CCC-----.........UAC.UC.U.CU-AAUUa..aAAU..AAa.aCAUUUGC
NW_007931122.1/31794421-31794342   AAUUUGUGCUGGUGGCAACAGAAUUCAUUAGCA..UAUUCAUUUCU.GAG.UA-CCC-----.........UAC.UC.U.CU-AAUUa..aAAU..AAa.aCAUUUGC
NW_010973023.1/4804652-4804731     AAUUUGUGCUGGUGGCAACAGAAUUCAUUAGCA..UAUUCAUUUCU.GAG.UA-CCC-----.........UAC.UC.U.CU-AAUUa..aAAU..AAa.aCAUUUGC
NW_004973349.1/1035957-1035878     AAUUUGUGCUGGUGGCAACAGAAUUCAUUAGCA..UAUUCAUUUCU.GAG.UA-CCC-----.........UAC.UC.U.CU-AAUUa..aAAU..AAa.aCAUUUGC
NW_008246059.1/2750797-2750718     AAUUUGUGCUGGUGGCAACAGAAUUCAUUAGCA..UAUUCAUUUCU.GAG.UA-CCC-----.........UAC.UC.U.CU-AAUUa..aAAU..AAa.aCAUUUGC
NW_009629003.1/47334-47413         AAUUUGUGCUGGUGGCAACAGAAUUCAUUAGCA..UAUUCAUUUCU.GAG.UA-CCC-----.........UAC.UC.U.CU-AAUUa..aAAU..AAa.aCAUUUGC
NW_005081547.1/4015629-4015708     AAUUUGUGCUGGUGGCAACAGAAUUCAUUAGCA..UAUUCAUUUCU.GAG.UA-CCC-----.........UAC.UC.U.CU-AAUUa..aAAU..AAa.aCAUUUGC
NW_020340258.1/1187134-1187213     AAUUUGUGCUGGUGGCAACAGAAUUCAUUAGCA..UAUUCAUUUCU.GAG.UA-CCC-----.........UAC.UC.U.CU-AAUUa..aAAU..AAa.aCAUUUGC
NW_004775541.1/4172887-4172966     AAUUUGUGCUGGUGGCAACAGAAUUCAUUAGCA..UAUUCAUUUCU.GAG.UA-CCC-----.........UAC.UC.U.CU-AAUUa..aAAU..AAa.aCAUUUGC
NC_034410.1/89389573-89389494      AAUUUGUGCUGGUGGCAACAGAAUUCAUUAGCA..UAUUCAUUUCU.GAG.UA-CCC-----.........UAC.UC.U.CU-AAUUa..aAAU..AAa.aCAUUUGC
NW_011950922.1/4825661-4825740     AAUUUGUGCUGGUGGCAACAGAAUUCAUUAGCA..UAUUCAUUUCU.GAG.UA-CCC-----.........UAC.UC.U.CU-AAUUa..aAAU..AAa.aCAUUUGC
NW_009987308.1/20424-20345         AAUUUGUGCUGGUGGCAACAGAAUUCAUUAGCA..UAUUCAUUUCU.GAG.UA-CCC-----.........UAC.UC.U.CU-AAUUa..aAAU..AAa.aCAUUUGC
NW_009664380.1/646717-646638       AAUUUGUGCUGGUGGCAACAGAAUUCAUUAGCA..UAUUCAUUUCU.GAG.UA-CCC-----.........UAC.UC.U.CU-AAUUa..aAAU..AAa.aCAUUUGC
NC_006089.5/90865315-90865236      AAUUUGUGCUGGUGGCAACAGAAUUCAUUAGCA..UAUUCAUUUCU.GAG.UA-CCC-----.........UAC.UC.U.CU-AAUUa..aAAU..AAa.aCAUUUGC
NW_005054311.1/9187899-9187820     AAUUUGUGCUGGUGGCAACAGAAUUCAUUAGCA..UAUUCAUUUCU.GAG.UA-CCC-----.........UAC.UC.U.CU-AAUUa..aAAU..AAa.aCAUUUGC
NW_019776314.1/1959618-1959697     AAUUUGUGCUGGUGGCAACAGAAUUCAUUAGCA..UAUUCAUUUCU.GAG.UA-CCC-----.........UAC.UC.U.CU-AAUUa..aAAU..AAa.aCAUUUGC
NW_016690220.1/171911-171990       AAUUUGUGCUGGUGGCAACAGAAUUCAUUAGCA..UAUUCAUUUCU.GAG.UA-CCC-----.........UAC.UC.U.CU-AAUUa..aAAU..AAa.aCAUUUGC
NW_010773887.1/7253-7332           AAUUUGUGCUGGUGGCAACAGAAUUCAUUAGCA..UAUUCAUUUCU.GAG.UA-CCC-----.........UAC.UC.U.CU-AAUUa..aAAU..AAa.aCAUUUGC
NW_015379230.1/92709627-92709548   AAUUUGUGCUGGUGGCAACAGAAUUCAUUAGCA..UAUUCAUUUCU.GAG.UA-CCC-----.........UAC.UC.U.CU-AAUUa..aAAU..AAa.aCAUUUGC
NW_004678171.1/431336-431257       AAUUUGUGCUGGUGGCAACAGAAUUCAUUAGCA..UAUUCAUUUCU.GAG.UA-CCC-----.........UAC.UC.U.CU-AAUUa..aAAU..AAa.aCAUUUGC
NW_018114010.1/1394617-1394696     AAUUUGUGCUGGUGGCAACAGAAUUCAUUAGCA..UAUUCAUUUCU.GAG.UA-CCC-----.........UAC.UC.U.CU-AAUUa..aAAU..AAa.aCAUUUGC
NW_010205399.1/31388-31467         AAUUUGUGCUGGUGGCAACAGAAUUCAUUAGCA..UAUUCAUUUCU.GAG.UA-CCC-----.........UAC.UC.U.CU-AAUUa..aAAU..AAa.aCAUUUGC
NW_010296684.1/20272-20193         AAUUUGUGCUGGUGGCAACAGAAUUCAUUAGCA..UAUUCAUUUCU.GAG.UA-CCC-----.........UAC.UC.U.CU-AAUUa..aAAU..AAa.aCAUUUGC
NW_020109688.1/38214077-38213998   AAUUUGUGCUGGUGGCAACAGAAUUCAUUAGCA..UAUUCAUUUCU.GAG.UA-CCC-----.........UAC.UC.U.CU-AAUUa..aAAU..AAa.aCAUUUGC
NW_009648539.1/1365616-1365695     AAUUUGUGCUGGUGGCAACAGAAUUCAUUAGCA..UAUUCAUUUCU.GAG.UA-CCC-----.........UAC.UC.U.CU-AAUUa..aAAU..AAa.aCAUUUGC
NW_015090810.1/10627955-10627876   AAUUUGUGCUGGUGGCAACAGAAUUCAUUAGCA..UAUUCAUUUCU.GAG.UA-CCC-----.........UAC.UC.U.CU-AAUUa..aAAU..AAa.gCAUUUGC
NW_015438579.1/5374432-5374511     AAUUUGUGCUGGUGGCAACGGAAUUCAUUAGCA..UAUUCAUUUCU.GAG.UA-CCC-----.........UAC.UC.U.CU-AAUUa..aAAU..AAa.aCAUUUGC
NC_029517.1/81821974-81821895      AAUUUGUGCUGGUGGCAACGGAAUUCAUUAGCA..UAUUCAUUUCU.GAG.UA-CCC-----.........UAC.UC.U.CU-AAUUa..aAAU..AAa.aCAUUUGC
NW_004994034.1/3223130-3223051     AAUUUGUGCUGGUGGCAAAAGAAUUCAUUAGCA..UAUUCAUUUCU.GAG.UA-CCC-----.........UAC.UC.U.CU-AAUUa..aAAU..AAa.aCAUUUGC
NW_004930275.1/874437-874516       AAUUUGUGCUGGUGGCAAAAGAAUUCAUUAGCA..UAUUCAUUUCU.GAG.UA-CCC-----.........UAC.UC.U.CU-AAUUa..aAAU..AAa.aCAUUUGC
NW_007619034.1/1285108-1285029     AAUUUGUGCUAGUGGCAACAGAAUUCAUUAGCA..UAUUCAUUUCU.GAG.UA-CCC-----.........UAC.UC.U.CU-AAUUa..aAAU..AAa.aCAUUUGC
NW_007359895.1/13910755-13910834   AAUUUGUGCUGGUGGCAACAGAAUUCAUUAGCA..UAUUCAUUUCU.GAG.UAUC-C-----.........UAC.UC.U.CU-AAUUa..aAAU..AAa.gCAUUUGC
NW_005854744.1/70288-70209         AAUUUGUGCUGGUGGCAACAGAAUUCAUUAGCA..UAUUCAUUUCU.GAG.UAUC-C-----.........UAC.UC.U.CU-AAUUa..aAAU..AAa.gCAUUUGC
NW_010460148.1/7254-7333           AAUUUGUGCUGGUGGCAACAGAAUUCAUUAGCA..UAUUCAUUUCU.GAG.UA-CCC-----.........UGC.UC.U.CU-AAUUa..aAAU..AAa.aCAUUUGC
NW_020455466.1/3483867-3483788     AAUUUGUGCUGGUGGCAACAGAAUUCAUUAGCA..UAUUCAUUUCU.GAG.UAGC-C-----.........UAC.UC.U.CU-AAUUa..aAAU..AAa.aCAUUUGC
NW_010581274.1/497389-497468       AAUUUGUGCUGGUGGCAACAGAAUUCAUUAGCA..UAUUCAUUUCU.GAG.UAGC-C-----.........UAC.UC.U.CU-AAUUa..aAAU..AAa.aCAUUUGC
NW_009902446.1/1562854-1562775     AAUUUGUGCUGGUGGCAAUAGAAUUCAUUAGCA..UAUUCAUUUCU.GAG.UA-CCC-----.........UAC.UC.U.CU-AAUUa..aAAU..AAa.aCCUUUGC
NW_004848281.1/18585041-18584962   AAUUUGUGCUGGUGGCAACAGAAUUCAUUAGCA..UAUUCAUUUCU.GAG.UA-CUC-----.........UAC.UC.U.CU-AAUUa..aAAU..AAa.aCAUUUGC
NW_002198279.1/4167135-4167056     AAUUUGUGCUGGUGGCAACAGAAUUCAUUAGCA..UAUUCAUUUCU.GAG.UA-CUC-----.........UAC.UC.U.CU-AAUUa..aAAU..AAa.aCAUUUGC
NC_011465.1/96273286-96273207      AAUUUGUGCUGGUGGCAACAGAAUUCAUUAGCA..UAUUCAUUUCU.GAG.UA-CUC-----.........UAC.UC.U.CU-AAUUa..aAAU..AAa.aCAUUUGC
NW_008649901.1/11286-11207         AAUUUGUGCUGGUGGCAACAGAAUUCAUUAGCA..UAUUCAUUUCU.GAG.UA-CUC-----.........UAC.UC.U.CU-AAUUa..aAAU..AAa.aCAUUUGC
NW_010132193.1/20500-20421         AAUUUGUGCUGGUGGCAACAGAAUUCAUUAGCA..UAUUCAUUUCU.GAG.UA-CUC-----.........UAC.UC.U.CU-AAUUa..aAAU..AAa.aCAUUUGC
NW_020664409.1/15944853-15944774   AAUUUGUGCUGGUGGCAAAAGAAUUCAUUAGCA..UAUUCAUUUCU.GAG.UAUC-C-----.........UAC.UC.U.CU-AAUUa..aAAU..AAa.gCAUUUGC
NW_009260128.1/1136576-1136497     AAUUUGUGCUGGUGGCAACAGAAUUCAUUAGCA..UAUUCAUUUCU.GAG.UA-CCC-----.........UAC.UC.U.CU-AAUUa..aAAU..AAa.aGAUUUGC
NW_008795036.1/3103294-3103215     AAUUUGUGCUGGUGGCAACAGAAUUCAUUAGCA..UAUUCAUUUCU.GAG.UA-CCC-----.........UAC.UC.U.CU-AAUUa..aAAU..AAa.aGAUUUGC
NW_009270794.1/387197-387118       AAUGUGUGCUGGUGGCAACAGAAUUCAUUAGCA..UAUUCAUUUCU.GAG.UAGC-C-----.........UAC.UC.U.CU-AAUUa..aAAU..AAa.aCAUUUGC
NW_020799095.1/90380916-90380839   AAUUUGUGCUGGUGGCAACAGAAUUCAUUAGCA..UAUUCAUUUCU.GAG.UA-CCC-----.........UAC.UC.U.AAUUAA-....AAU..AAa.aCAUUUGC
NW_005087555.1/57380378-57380457   AAUUUGUGCUGGUGGCAACAGAAUUCAUUAGCA..UAUUCAUUUCU.GAG.UA-CCC-----.........UAC.UC.U.CU-AAUUa..aAAU..AAa.aCAUUUAC
NW_020452704.1/671572-671493       AAUUUGUGCUGGUGGCAACAGAAUUCAUUAGCA..UAUUCAUUUCU.GAG.UAGC-U-----.........UAC.UC.U.CU-AAUUa..aAAU..AAa.aCAUUUGC
NW_012001949.1/5625005-5625087     AAUGUGUGCUGGUGGCAAUGCCAUUCAUUAGCAuaUUCA-UUU-CUgAGG.AGUC--AGUUA.........CUCuCU.A.AUUAAAA....UGG..AA...CAUUUGC
NG_032949.1/129039-128957          AAUGUGUGCUGGUGGCAAUGCCAUUCAUUAGCAuaUUCA-UUU-CUgAGG.AGUC--AGUUA.........CUCuCU.A.AUUAAAA....UGG..AA...CAUUUGC
NT_010966.15/55459946-55460028     AAUGUGUGCUGGUGGCAAUGCCAUUCAUUAGCAuaUUCA-UUU-CUgAGG.AGUC--AGUUA.........CUCuCU.A.AUUAAAA....UGG..AA...CAUUUGC
NC_000018.10/76371152-76371234     AAUGUGUGCUGGUGGCAAUGCCAUUCAUUAGCAuaUUCA-UUU-CUgAGG.AGUC--AGUUA.........CUCuCU.A.AUUAAAA....UGG..AA...CAUUUGC
NW_006735267.1/3575222-3575140     AAUGUGUGCUGGUGGCAAUGCCAUUCAUUAGCAuaUUCA-UUUUUG.AGG.AGUC--AGUUA.........CUCuCU.A.AUUAAAA....UGG..AA...CAUUUGC
NC_023659.1/3575222-3575140        AAUGUGUGCUGGUGGCAAUGCCAUUCAUUAGCAuaUUCA-UUUUUG.AGG.AGUC--AGUUA.........CUCuCU.A.AUUAAAA....UGG..AA...CAUUUGC
NW_010526492.1/20045-19966         AAUUUGUGCUGAUGGCAACAGAAUUCAUUAGCA..UAUUCAUUUCU.GAG.UA-CCC-----.........UAC.UC.U.CU-AAUUa..aAAU..AAa.aGAUUUGC
NW_010398109.1/20006-19927         AAUUUGUGCUGGUGGCAAAAGAAUUCAUUAGCA..UAUUCAUUCCU.GAG.UA-CCC-----.........UAC.UC.C.CU-AAUUa..aAAU..AAa.aCAUUUGC
```

```
NW_010060426.1/22279-22358             AAUUUGUGCUGGUGGCAACGGAAUUCAUUAGCA..UACUCAUUUCU.GAG.UA-CCC-----.........UAC.UC.U.CU-AAUUa..aAAU..AAa.aCAUUUGC
NW_001581902.1/52484441-52484520       AAUUUGUGCUGGUGGCAAUAGAAUUCAUUAGCA..UAUUUAUUUCU.GAG.UAGC-C-----.........UAC.UC.U.CU-AAUUa..aAAUgg--a.aCAUUUGC
NC_008803.1/305254314-305254393        AAUUUGUGCUGGUGGCAAUAGAAUUCAUUAGCA..UAUUUAUUUCU.GAG.UAGC-C-----.........UAC.UC.U.CU-AAUUa..aAAUgg--a.aCAUUUGC
NW_019317552.1/3448326-3448244         CAUGUGUGCUGGUGGCAAUGCCAUUCAUCAGCAuaUUCA-UUUUUG.AGG.AGUC--AGUUA.........CUCuCU.A.AUUAAAA....UGG..AA...CAUUUGC
NW_008621346.1/20009-19930             AAUUUGUGCUGGUGGCAACAGAAUUCAUUAGCA..UAUUCAUUUCU.GAG.UA-CCC-----.........UAC.AC.U.CU-AAUUa..aAAU..AAa.aCAUUUGC
NW_009168033.1/20983-20904             AAUUUGUGCUGGUGGCAACAGAAUUCAUUAGCA..UAUUCAUUUCU.GAG.UA-CCC-----.........UAC.UC.U.CU-AAUUa..aAAU..ACa.aGAUUUGC
NW_010252268.1/19563-19484             AAUUCGUGCUGGUGGCAACAGAAUUCAUUAGCA..UAUUCAUUUCU.GAG.UA-CCC-----.........UAC.UC.U.CU-AAUUa..aAAU..ACa.aCAUUUGC
NW_007778829.1/2762-2683               AAUUUGUGCUGGUGGCAACAGAAUUCAUUAGCAuaUUCA-UUU-CUgAGG.AG-CCC----A.........CUCuCU.A.AUUAAAA....UGG..AA...CAUUUGC
NW_008824908.1/888664-888743           AAUUUGUGCUGGUGGCAACAGAAUUCAUUAGCA..UAUUCAUUUCU.GAG.UA-CCC-----.........UAC.UC.U.CU-AAUUa.aa-AG..AAa.aGAUUUGC
NW_009245600.1/2083075-2082996         AAUUUGUGCUGGUGGCAAUAGAAUUCAUUAGCA..UAUUCAUUUCU.GAG.UA-CCC-----.........UAG.UC.U.CU-AAUUa..aAAU..AAa.aCAUUUGC
NW_017493710.1/74166436-74166518       AACGUGUGCUGGUGGCAAUGCCAUUCAUUAGCAuaUUCA-UUU-CUgAGG.AGUC--AGUUA.........CUCuCU.A.AUUAAAA....UGG..AA...CAUUUGC
NC_018442.2/74166436-74166518          AACGUGUGCUGGUGGCAAUGCCAUUCAUUAGCAuaUUCA-UUU-CUgAGG.AGUC--AGUUA.........CUCuCU.A.AUUAAAA....UGG..AA...CAUUUGC
NW_020251522.1/69136357-69136439       AAUGAGUGCUGGUGGCAAUGCCAUUCAUUAGCAuaUUCA-UUU-CUgAGG.AGUC--AGUUA.........CUCuCU.A.AUUAAAA....UGG..AA...CAUUUGC
NC_037686.1/69136357-69136439          AAUGAGUGCUGGUGGCAAUGCCAUUCAUUAGCAuaUUCA-UUU-CUgAGG.AGUC--AGUUA.........CUCuCU.A.AUUAAAA....UGG..AA...CAUUUGC
NW_012116121.1/3203451-3203533         AAUGUGUGCUGGUGGCAAUGCCAUUCAUUAGCAuaUUCA-UUUUUG.AGG.AGUC--AGUUA.........CUCuCU.A.AUUAAAA....UGG..AA...GAUUUGC
NW_019932863.1/4165259-4165177         AAUGUGUGCUGGUGGCAAUGCCAUUCAUUAGCAuaUUCA-UUU-CUgAGG.AGUC--AGUUA.........CUCuCU.A.AUUAAAA....CGG..AA...CAUUUGC
NC_036897.1/70609211-70609293          AAUGUGUGCUGGUGGCAAUGCCAUUCAUUAGCAuaUUCA-UUU-CUgAGG.AGUC--AGUUA.........CUCuCU.A.AUUAAAA....CGG..AA...CAUUUGC
NW_014014338.1/29258482-29258564       AAUGUGUGCUGGUGGCAAUGCCAUUCAUUAGCAuaUUCA-UUU-CUgAGG.AGUC--AGUUA.........CUCuCU.A.AUUAAAA....CGG..AA...CAUUUGC
NC_027886.1/73034687-73034769          AAUGUGUGCUGGUGGCAAUGCCAUUCAUUAGCAuaUUCA-UUU-CUgAGG.AGUC--AGUUA.........CUCuCU.A.AUUAAAA....CGG..AA...CAUUUGC
NW_018343978.1/20079996-20079917       AAUUUGUGCUGGUGGCAAUAGAAUUCAUUAGCA..UAUUCAUUUCU.GAG.UAGC-C-----.........UGC.UC.U.CU-AAUUa..aAAUggAA...CAUUUGC
NW_010687662.1/6552-6631               AAUUUGUGCUGGUGGCAAGAGAAUUCAUUAGCA..UAUUCAUUUCU.GAG.UA-CCC-----.........UGC.UC.U.CU-AAUUa..aAAC..AAa.aCAUUUGC
NW_003823347.1/481854-481933           AAUUUGUGCUGGUGGCAAUAGAAUUCAUUAGCA..UAUUCAUUUUU.GAG.UAGC-C-----.........UAC.UC.U.CU-AAUUaaaaUGG..AA...CAUUUGC
NW_017728886.1/166606881-166606802     AAUUUGUGCUGGUGGCAACAGAAUUCAUUAGCA..UAUUCAUUUCU.AAG.UAUC-C-----.........UAC.UC.U.CU-AAUUa..aAGU..AAa.aCAUUUGC
NW_017728992.1/72136496-72136417       AAUUUGUGCUGGUGGCAACAGAAUUCAUUAGCA..UAUUCAUUUCU.AAG.UAUC-C-----.........UAC.UC.U.CU-AAUUa..aAGU..AAa.aCAUUUGC
NW_017710615.1/20658303-20658382       AAUUUGUGCUGGUGGCAACAGAAUUCAUUAGCA..UAUUCAUUUCU.AAG.UAUC-C-----.........UAC.UC.U.CU-AAUUa..aAGU..AAa.aCAUUUGC
NW_010829838.1/2748279-2748197         AAUGUGUGCUGGUGGCAAUGCCAUUUAUUAGCAuaUUCA-UUUUUG.AGG.AGUC--AGUUA.........CUCuCU.A.AUUAAAA....UGG..AA...CAUUUGC
NW_016841755.1/2425863-2425945         AAUGUGUGCUGGUGGCAAUGCCAUUUAUUAGCAuaUUCA-UUUUUG.AGG.AGUC--AGUUA.........CUCuCU.A.AUUAAAA....UGG..AA...CAUUUGC
NW_009086916.1/11363-11284             AAUUUGUGCUGGUGGCAACAGAAUUCAUUAGCA..UAUUCAUUUCU.GAG.UA-CCC-----.........UAC.UC.U.CU-AAUUa..aAAU..AAa.aGACUCGC
NW_018655836.1/5649075-5648996         AAUUUGUGCUGGUGGCAACAGAAUUCAUUAGCA..UAUUCAUUUCU.GAA.UA-CUC-----.........UAC.UC.U.CU-AAUUa..aAAU..AAa.aCAUUUGC
NW_019937248.1/71977435-71977513       AAUGUGUGCUGGUGGCAAUGCCAUUCAUUAGCAuaUUCA-UUU-CUgAGG.AGUC------A.........CUCuCU.A.AUUAAAA....UGG..AA...CAUUUGC
NC_036921.1/71977435-71977513          AAUGUGUGCUGGUGGCAAUGCCAUUCAUUAGCAuaUUCA-UUU-CUgAGG.AGUC------A.........CUCuCU.A.AUUAAAA....UGG..AA...CAUUUGC
NW_012101587.1/3950510-3950428         AAUGUGUGCUGCUGGCAAUGCCAUUCAUUAGCAuaUUCA-UUU-CUgAGG.AGUC--AGUUA.........CUCuCU.A.AUUAAAA....UGG..AA...CAUUUGC
NW_018759898.1/3896-3978               AAUGAGUGCUGGUGGCAAUGCCAUUCAUUAGCAuaUUCA-UUU-CU.GAGaAGUC--AGUUA.........CUC.UC.UaAUUAAAA....UGG..AA...CAUUUGC
NC_018169.2/68845753-68845835          AAUGAGUGCUGGUGGCAAUGCCAUUCAUUAGCAuaUUCA-UUU-CU.GAGaAGUC--AGUUA.........CUC.UC.UaAUUAAAA....UGG..AA...CAUUUGC
NW_004087883.1/3869337-3869255         AAUGUGUGCUGGUAGCAAUGCCAUUCAUUAGCAuaUUCA-UUU-CUgAGG.AGUC--AGUUA.........CUCuCU.A.AUUAAAA....UGG..AA...CAUUUGC
NC_019819.1/3870337-3870255            AAUGUGUGCUGGUAGCAAUGCCAUUCAUUAGCAuaUUCA-UUU-CUgAGG.AGUC--AGUUA.........CUCuCU.A.AUUAAAA....UGG..AA...CAUUUGC
NW_009882014.1/27971-28051             AAUUUGCGCUGGUGGCAACGGAAUUCAUUAGCA..UAUUCAUUUCU.GAG.UA-CCC-----.........UGC.UC.U.CU-AAUUa..aAAU..AAaacAAUUUGC
NW_006203974.1/96109-96188             AAUUUGUGCUGGUGGCAAUACAAUUCAUUAGCAuaUUCA-UUU-CU.GAG.AUGUCU----A.........CUC.UCuA.AUUAAAA....UGG..AA...CAUUUGC
NW_005842935.1/305235-305156           AAUUUGUGCUGGUGGCAACAGAAUUCAUUAGCA..UAUUCAUUUCU.AAG.UAUC-C-----.........UAC.UC.U.CU-AAUUa..aAGU..AAa.aCAUGUGC
NW_014805709.1/12642620-12642702       AAUGUGUGCUGGUGGCAAUGCCGUUUAUUAGCAuaUUCA-UUU-CUgAGG.AGUC--AGUUA.........CUCuCU.A.AUUAAAA....UGG..AA...CAUUUGC
NC_027910.1/66522500-66522582          AAUGUGUGCUGGUGGCAAUGCCGUUUAUUAGCAuaUUCA-UUU-CUgAGG.AGUC--AGUUA.........CUCuCU.A.AUUAAAA....UGG..AA...CAUUUGC
NW_005092996.1/3734942-3734860         AAUGUGUGCUGGUGGCAAUGCCGUUUAUUAGCAuaUUCA-UUU-CUgAGG.AGUC--AGUUA.........CUCuCU.A.AUUAAAA....UGG..AA...CAUUUGC
NC_022289.1/3734942-3734860            AAUGUGUGCUGGUGGCAAUGCCGUUUAUUAGCAuaUUCA-UUU-CUgAGG.AGUC--AGUUA.........CUCuCU.A.AUUAAAA....UGG..AA...CAUUUGC
NW_012011145.1/3931953-3931871         AAUGUGUGCUGGUGGCAAUGCCGUUUAUUAGCAuaUUCA-UUU-CUgAGG.AGUC--AGUUA.........CUCuCU.A.AUUAAAA....UGG..AA...CAUUUGC
NW_004624806.1/4211622-4211545         CAUUUGUGCUAGUGGCAAUAUAAUUCAUUAGCAuaUUCA--UUUCUgAAG.AGUCUA-----.........CUC.UA.A.UU-AAAA....UGG..AA...CAUUUGC
NW_007248835.1/117370-117288           AAUUUGUGCUGGUGGCAAUCCUGUCCGUUAGCA..UACUCAUUUCU.AGG.GAAUCAGUUAC.........UCU.CU.A.AUUAAAA....UGG..AA...CAUUUGC
NW_003184336.1/4582136-4582216         GAUGUGUGCUGGUGGCAAUGCCGUUCAUUAACA..UUCA-UUU-CUgAGG.AGUC--AGUUA.........CUCuCU.A.AUUAAAA....UGA..AA...CAUUCGC
NC_013908.1/113975082-113975162        GAUGUGUGCUGGUGGCAAUGCCGUUCAUUAACA..UUCA-UUU-CUgAGG.AGUC--AGUUA.........CUCuCU.A.AUUAAAA....UGA..AA...CAUUCGC
NW_004955402.1/59760853-59760930       CAUUUGUGCUCGUGGCAAUAGAAUUCAUUAGCAuaUUCA--UUUCUgAAG.AGUCUA-----.........CUC.UA.A.UU-AAAA....UGG..AA...CAUUUGC
NW_011046472.1/3140956-3140879         CAUUUGUGCUAGUGGCAAUAUAAUUCAUUAGCA..UAUUCAUUUCUgAAG.AGUCUA-----.........CUC.UA.A.UUAAAAC....-GG..AA...CAUUUGC
NW_006607785.1/1433884-1433805         AAUUUGUGCUGGUGGCAACAGAAUUCAUUUGCA..UAUUCAUUUCC.GAG.UAUC-C-----.........UAC.UC.U.CU-AAUUa..aAAU..AAa.gCAUUUGG
NW_004524696.1/4255454-4255531         CAUUUGUGCUCAUGGCAAUAGAAUCCAUUAGCAuaUUCA--UUUCUgAAG.AGUCUA-----.........CUC.UA.A.UU-AAAA....UGG..AA...CAUUUGC
NW_004454163.1/42414260-42414339       AGUGUGUGCUGGUGGCAGCAAAAUUCAUUAGCAuaUUCA-UUUUUG.AGG.AGUCUA-----.........CUCuCU.A.AUUAAAA....UGG..AA...CAUUUGU
NW_009315996.1/15425-15346             UAUUUGUGCUGGUGGCAACAGAAUUCAUUUGCA..UAUUCAUUUCU.GAC.UA-CCC-----.........UAU.UC.U.CU-AAUUa..aAAU..AAa.aGAUUUGC
NW_012148574.1/1686587-1686665         AGUUUGUGCUGGUGGCAAUACAACUCAUUAGCAuaUUCA-UUU-CUgAGG.AGUC--GGUUA.........--C.-U.A.AUUAAAA....UGG..AA...CAUUUGC
NW_009209841.1/20704-20625             AGUUUGUGCUGGUGGCAACAGAAUUCAUUAGCA..UAUUCAUUUCU.G--.-----------caucccua---.-C.U.CUCUAAUu.aaAAU..AAa.aGAUUUGC
NW_018502140.1/2815506-2815424         AAUGUGUGCUGGUGGCAAUGUCGUUCGUUAACAuaUUCA-UUU-CUgAGG.AGUC--AGUUA.........CUCuCU.A.AUUAAAA....UGG..AA...CAUUCGC
#=GC SS_cons                           :::::<<<<<<<<<<_________>>>>>>>>>..><<<-<<<---.<<<.<<_________~~~~~~~~~>>>.>>.-.---->>>....>>>..::...:::::::
#=GC RF                                aAUuUGuGCUcgUGgCAaUugcAUuCAugAGCa..UccuuuuuUCU.gaG.gagCCCgcUag~~~~~~~~~ucC.uc.u.cUuaaaa....agg..AA...CAUUUGC
//
```

c *MYH7-3778*

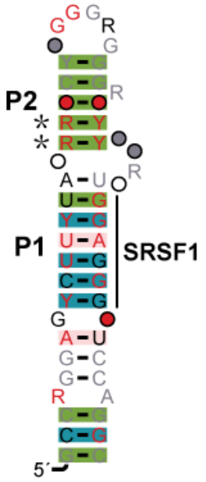

Sequence alignment for the motif *MYH7-3778*

```
# STOCKHOLM 1.0
NW_019873550.1/28988145-28988094       GCCAGGAGCCUUCUA....GAGCCCUGGGG.aGGGGCUCUGAUGGAGGGGUC-AGGC
NW_004438590.1/3742933-3742984         GCCAGGAGCCUUCUA....GAGCCCUGGGG.aGGGGCUGUGAUGGAGGGGUC-AGGC
NW_004438590.1/3773024-3773075         GCCAGGAGCCUUCUA....GAGCCCUGGGG.aGGGGCUGUGAUGGAGGGGUC-AGGC
NW_020173974.1/135005-134954           GCCAGGAGCCUUCUA....GAGCCCUGGGG.aGGGGCUGUGAUGGAGGGGUC-AGGC
NW_019161577.1/2689-2638               GCCAGGAGCCUUCUA....GAGCCCUGGGG.aGGGGCUGUGAUGGAGGGGUC-AGGC
NW_017844172.1/60780-60831             GCCAGGAGCCUUCUA....GAGCCCUGGGG.aGGGGCUGUGAUGGAGGGGUC-AGGC
NW_019873550.1/28931616-28931565       GCCAGGAGUCUUCUA....GAGCCCUGGGG.aGGGGCUCUGAUGGAGGGGUC-AGGC
NW_019873550.1/28982525-28982474       GCCAGGAGUCUUCUA....GAGCCCUGGGG.aGGGGCUCUGAUGGAGGGGUC-AGGC
NG_007884.1/20350-20397                GCCGAGAGCCUUUUA....-GAGCC-GGGG..-GGAUUCCAG-UGGAGGGGUCCAGGC
NW_017493703.1/21954377-21954424       GCCGAGAGCCUUUUA....-GAGCC-GGGG..-GGAUUCCAG-UGGAGGGGUCCAGGC
NC_018438.2/21954377-21954424          GCCGAGAGCCUUUUA....-GAGCC-GGGG..-GGAUUCCAG-UGGAGGGGUCCAGGC
NC_000014.9/23420312-23420265          GCCGAGAGCCUUUUA....-GAGCC-GGGG..-GGAUUCCAG-UGGAGGGGUCCAGGC
NT_026437.13/5196789-5196742           GCCGAGAGCCUUUUA....-GAGCC-GGGG..-GGAUUCCAG-UGGAGGGGUCCAGGC
NW_005871345.1/163743-163794           GCCAGGAGUCUUCUAgagcUGGGGGAGGGG..GCU-CCC---AUGGAGGGCUCCAGGC
NW_006726798.1/11738943-11738892       GCCAGGAGCCUUCUA....GAGCCCUGGGG.aGGGGCUCUGAUGGAGGGGUC-CGGC
NW_005370741.1/169936-169987           GCCAGGAGUUUUCUAgagcUGGGGGAGGGG..GCU-CCC---AUGGAGGGCUCCAGGC
NW_006774793.1/1609870-1609819         GCCGGGAGCCUUCUA....GAGCCCUGGGG.aGGGGCUGUGACGGAGGGGUC-AGGC
NW_006774793.1/1579062-1579011         GCCAGGAGCCUUCUA....GAGCCCUGGGG.gGGGGCUGUGACGGAGGGGUC-AGGC
NW_017844172.1/51947-51998             GCCAGGAGCCUUCUA....GAGCCCUGGGG.aGGGGCUGUGAUAGAGGGGUC-AGGC
NW_007370840.1/879161-879212           GCCAGGAGUCUUCUAgagcCGGGGGAGGGG..GCU-CCC---AUGGAGGGUUCCAGGC
NC_019837.1/40897044-40897092          GCCGAGAGCCUUUUA....-GAGCC-GGGG..GGAAUUCCAG-UGGAGGGGUCCAGGC
NW_004087910.1/40896044-40896092       GCCGAGAGCCUUUUA....-GAGCC-GGGG..GGAAUUCCAG-UGGAGGGGUCCAGGC
NW_005370741.1/201654-201705           GCCAGGAAUCUUCUAgagcUGGGGGAGGAG..GCU-CCC---AUGGAGGGCUCCAGGC
NC_036917.1/4139600-4139552            GCCGAGAGCCUUUUA....-GAACC-GGGG..GGGAUUCCAG-UGGAGGAGUCCAGGC
NW_019937237.1/84823818-84823866       GCCGAGAGCCUUUUA....-GAACC-GGGG..GGGAUUCCAG-UGGAGGAGUCCAGGC
NW_017731735.1/878689-878740           GCCAGGAGUCUUCUA....-GAGCCAGGGGagGGGGCUCCCA-CGGAGGGUUCCAGGC
NW_017731735.1/906733-906784           GCCAGGAGUCUUCUA....-GAGCCAGGGGagGGGGCUCCCA-CGGAGGGUUCCAGGC
NW_006436757.1/703771-703822           GCCAGGAGUCUUCUA....GAGUCUAGGGA..GGGGGCUCUGAUGGAGGGUUCCAGGC
NW_006300366.1/1068853-1068802         GCCAGGAGUCUUCUAgagcUGAGGGAGGGG..GCU-CCC---AUGGAGGGCUCCAGGC
NW_018328240.1/541135-541084           GCCAGGAGCCUUCUA....GAGCCCUGGGA..GGGGGCUCCGAUGGAGGGGCCCAGGC
NW_020173974.1/164722-164671           GCCAGGAGCCUUCUA....GAGCCUUGGGG.aGGGGCUCUGAUGGAGGGGUC-AGGU
NW_020251511.1/24672652-24672606       CCUGGUAGCCUUUUA....-GAGCC-GGGG..-GGAUUCCAG-UGGAGGGGU-CCAGG
NC_022278.1/87110643-87110597          CCUGGUAGCCUUUUA....-GAGCC-GGGG..-GGAUUCCAG-UGGAGGGGU-CCAGG
NW_012104032.1/1308787-1308741         CCUGGUAGCCUUUUA....-GAGCC-GGGG..-GGAUUCCAG-UGGAGGGGU-CCAGG
NW_012002324.1/672420-672466           CCUGGUAGCCUUUUA....-GAGCC-GGGG..-GGAUUCCAG-UGGAGGGGU-CCAGG
NW_010822021.1/979262-979216           CCUGGUAGCCUUUUA....-GAGCC-GGGG..-GGAUUCCAG-UGGAGGGGU-CCAGG
NW_014805033.1/1309019-1308973         CCUGGUAGCCUUUUA....-GAGCC-GGGG..-GGAUUCCAG-UGGAGGGGU-CCAGG
NW_012012267.1/385551-385597           CCUGGUAGCCUUUUA....-GAGCC-GGGG..-GGAUUCCAG-UGGAGGGGU-CCAGG
NC_037675.1/24672652-24672606          CCUGGUAGCCUUUUA....-GAGCC-GGGG..-GGAUUCCAG-UGGAGGGGU-CCAGG
NW_005092973.1/22604598-22604552       CCUGGUAGCCUUUUA....-GAGCC-GGGG..-GGAUUCCAG-UGGAGGGGU-CCAGG
NC_027899.1/84742973-84742927          CCUGGUAGCCUUUUA....-GAGCC-GGGG..-GGAUUCCAG-UGGAGGGGU-CCAGG
NW_016828033.1/1121784-1121738         CCUGGUAGCCUUUUA....-GAGCC-GGGG..-GGAUUCCAG-UGGAGGGGU-CCAGG
NW_006726798.1/11768302-11768251       GCCAGGAACCUUCUA....GAGCCCUGGGG.aGGGGCUCUGAUGAAGGGGUC-CGGC
NW_005871345.1/193143-193194           GUCAAGAGUCUUCUAgagcUGGGGGAGGGG..GCU-CCC---AUGGAGGGCUCCAGGC
NC_036893.1/4327136-4327089            GCCGAGAGCCUUUUA....-GAGCC-GGGG..-GGAUUCCAG-UGGAGGGGUCCAGCC
NC_027882.1/22333429-22333382          GCCGAGAGCCUUUUA....-GAGCC-GGGG..-GGAUUCCAG-UGGAGGGGUCCAGCC
NW_014014215.1/535382-535429           GCCGAGAGCCUUUUA....-GAGCC-GGGG..-GGAUUCCAG-UGGAGGGGUCCAGCC
NW_019932847.1/4327136-4327089         GCCGAGAGCCUUUUA....-GAGCC-GGGG..-GGAUUCCAG-UGGAGGGGUCCAGCC
NC_036917.1/4109836-4109790            CCUGGUAGCCUUUCA....-GAGCC-GGGG..-GGAUUCCAG-UGGAGGGGU-CCAGG
NW_019937237.1/84853582-84853628       CCUGGUAGCCUUUCA....-GAGCC-GGGG..-GGAUUCCAG-UGGAGGGGU-CCAGG
NG_023444.1/22753-22799                CCUGGUAGCUUUUCA....-GAGCC-GGGG..-GGAUUCCAG-UGGAGGGGU-CCAGG
NW_017493703.1/21984185-21984231       CCUGGUAGCUUUUCA....-GAGCC-GGGG..-GGAUUCCAG-UGGAGGGGU-CCAGG
NC_018438.2/21984185-21984231          CCUGGUAGCUUUUCA....-GAGCC-GGGG..-GGAUUCCAG-UGGAGGGGU-CCAGG
NC_000014.9/23390525-23390479          CCUGGUAGCUUUUCA....-GAGCC-GGGG..-GGAUUCCAG-UGGAGGGGU-CCAGG
NT_026437.13/5167002-5166956           CCUGGUAGCUUUUCA....-GAGCC-GGGG..-GGAUUCCAG-UGGAGGGGU-CCAGG
NW_011888994.1/3694812-3694761         GCCAGGAGUCUUCUA....GAGUCUCGGGA..GGGGGCUCUGAUGGAAGGUUCCAGGC
NW_006300366.1/1040293-1040242         GUCAGGAGUCUUCUAgagcUGAGGGAGGGG..GCU-CCC---AUGGAGGGCUCCAGGC
NW_018756305.1/419692-419646           CCUGGUAGCCUUUUA....-GAGCU-GGGG..-GGAUUCCAG-UGGAGGGGU-CCAGG
NC_018158.2/80392989-80392943          CCUGGUAGCCUUUUA....-GAGCU-GGGG..-GGAUUCCAG-UGGAGGGGU-CCAGG
NW_020251511.1/24702385-24702338       GCCGAGAGCCUUUUA....-GAACCAGGU-..-GGAAUCCAG-UGGAGGGGUCCAGGC
NW_006735279.1/22791157-22791110       GCCGAGAGCCUUUUA....-GAACCAGGU-..-GGAAUCCAG-UGGAGGGGUCCAGGC
NC_037675.1/24702385-24702338          GCCGAGAGCCUUUUA....-GAACCAGGU-..-GGAAUCCAG-UGGAGGGGUCCAGGC
NW_012122709.1/4579892-4579939         GCCGAGAGCCUUUUA....-GAACCAGGU-..-GGAAUCCAG-UGGAGGGGUCCAGGC
NW_018328240.1/511979-511928           GCCAAGAGCCUUCUA....GAGUCCUGGGA..GGGGGCUCCGAUGGAGGGGCCCAGGC
NC_022278.1/87140489-87140442          GCCAAGAGCCUUUUA....-GAACCAGGU-..-GGAAUCCAG-UGGAGGGGUCCAGGC
```

```
NW_014805033.1/1338871-1338824          GCCAAGAGCCUUUUA....-GAACCAGGU-..-GGAAUCCAG-UGGAGGGGUCCAGGC
NW_012012267.1/355555-355602            GCCAAGAGCCUUUUA....-GAACCAGGU-..-GGAAUCCAG-UGGAGGGGUCCAGGC
NW_005092973.1/22634444-22634397        GCCAAGAGCCUUUUA....-GAACCAGGU-..-GGAAUCCAG-UGGAGGGGUCCAGGC
NC_027899.1/84772825-84772778           GCCAAGAGCCUUUUA....-GAACCAGGU-..-GGAAUCCAG-UGGAGGGGUCCAGGC
NW_012148618.1/223337-223389            GCCAGGAGCCUUUUG....GAGCCCAGGGA.uGGGGGCUCUGGCGGAGGGGUCCAAGC
NW_003944339.1/3635-3682                CCUGGUAGCCUUUUA....-GAGCCCAGGG..-GGAUUCCAG-UGGAGGGGU-CCAGG
NW_006735279.1/22761797-22761751        CCCGGUAGCCUUUUA....-GAGCC-GGGG..-GGAUUCCAG-UGGAGGGGU-CCAGG
#=GC SS_cons                            <<<-<<<-<<<<<<<....-<<<<<_____.._>>->>>---->>>>>>>->>>->>>
#=GC RF                                 GCCGGGAGCCUUCUA....uGGgccaGGGG..GggAcCCcaGAUGGAGGGGUCCAGGC
//
```

d

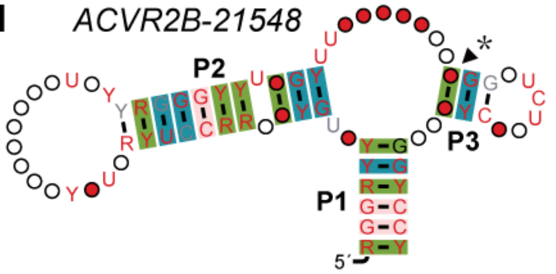

Sequence alignment for the motif *ACVR2B-21548*

```
# STOCKHOLM 1.0
NC_036906.1/103458513-103458439      AGGGCU.U-UGCAAAACCUUGAUUUgccc...............cgguUUCCAGGGUUUUGUUUUUGACAUUGGgUUCU.uUCCA.GCGGCCCU
NW_019937212.1/90197743-90197817     AGGGCU.U-UGCAAAACCUUGAUUUgccc...............cgguUUCCAGGGUUUUGUUUUUGACAUUGGgUUCU.uUCCA.GCGGCCCU
NG_011791.1/29424-29498              AGGGCU.U-UGCAAAACCUUGAUUUgccc...............cgguUACCAGGGUUUUGUUUUUGACAUUGGgUUCU.uUCCA.GCGGCCCU
NW_014013864.1/4089612-4089538       AGGGCU.U-UGCAAAACCUUGAUUUgccc...............cgguUACCAGGGUUUUGUUUUUGACAUUGGgUUCU.uUCCA.GCGGCCCU
NT_022517.19/38468722-38468796       AGGGCU.U-UGCAAAACCUUGAUUUgccc...............cgguUACCAGGGUUUUGUUUUUGACAUUGGgUUCU.uUCCA.GCGGCCCU
NC_000003.12/38478722-38478796       AGGGCU.U-UGCAAAACCUUGAUUUgccc...............cgguUACCAGGGUUUUGUUUUUGACAUUGGgUUCU.uUCCA.GCGGCCCU
NC_027871.1/38657458-38657532        AGGGCU.U-UGCAAAACCUUGAUUUgccc...............cgguUACCAGGGUUUUGUUUUUGACAUUGGgUUCU.uUCCA.GCGGCCCU
NW_006789542.1/87865-87782           AGGGCU.UC-GCAAAACUUUGAUUUgcaaaaccu.....caaucugguUUUCAGGGUUUUGUUUUGCCAUCAGG.UUCU..GCUUaGGGGCCCU
NC_018427.2/39173304-39173378        AGGGCU.U-UGCAAAACCUUGAUUUgccc...............ugguUACCAGGGUUUUGUUUUUGACAUUGGgUUCU.uUCCA.GCAGCCCU
NW_017493682.1/39173304-39173378     AGGGCU.U-UGCAAAACCUUGAUUUgccc...............ugguUACCAGGGUUUUGUUUUUGACAUUGGgUUCU.uUCCA.GCAGCCCU
NW_019932799.1/53038383-53038309     AGGGCU.U-UGCAAAACCUUGAUUUgccc...............cgguUACCAGGGUUUUGUUUUUGACAUUGGgUUCU.uUCCA.GCAGCCCU
NC_036882.1/38664185-38664259        AGGGCU.U-UGCAAAACCUUGAUUUgccc...............cgguUACCAGGGUUUUGUUUUUGACAUUGGgUUCU.uUCCA.GCAGCCCU
NW_020172172.1/1629051-1629139       AGGGCU.UC-GCAAAACUUUGAUUUgcaaaaccucaauccagucugguUUUCAGGGUUUUGUUUUGCCAUCAGG.UUCU..GCUUaGGGGCCCU
NW_004438508.1/4912395-4912307       AGGGCU.UC-GCAAAACUUUGAUUUgcaaaaccucaauccagucugguUUUCAGGGUUUUGUUUUGCCAUCAGG.UUCU..GCUUaGGGGCCCU
NW_019873593.1/2263660-2263572       AGGGCU.U-UGCAAAACUUUGAUUUgcaaaaccucaauccagucugguUUUCAGGGUUUUGUUUUGACAUCAGG.UUCU..GCUUaGAGGCCCU
NW_012003613.1/5940512-5940438       AGGGCU.U-UGCAAAACCUUGAUUUgccc...............cgguUUCCAAGGUUUUGUUUUUGACAUUGAgUUCU.uUCCA.GCGGCCCU
NG_011791.1/29614-29673              GGGAUC.ACUGUU-GGCCUCA-UGC.......................U-CUGGGGCCUAGCUUAAUAC--AGG.-UCUucUCCU.--GGUCCC
NW_014013864.1/4089422-4089363       GGGAUC.ACUGUU-GGCCUCA-UGC.......................U-CUGGGGCCUAGCUUAAUAC--AGG.-UCUucUCCU.--GGUCCC
NW_019932799.1/53038193-53038134     GGGAUC.ACUGUU-GGCCUCA-UGC.......................U-CUGGGGCCUAGCUUAAUAC--AGG.-UCUucUCCU.--GGUCCC
NC_036882.1/38664375-38664434        GGGAUC.ACUGUU-GGCCUCA-UGC.......................U-CUGGGGCCUAGCUUAAUAC--AGG.-UCUucUCCU.--GGUCCC
NT_022517.19/38468912-38468971       GGGAUC.ACUGUU-GGCCUCA-UGC.......................U-CUGGGGCCUAGCUUAAUAC--AGG.-UCUucUCCU.--GGUCCC
NC_000003.12/38478912-38478971       GGGAUC.ACUGUU-GGCCUCA-UGC.......................U-CUGGGGCCUAGCUUAAUAC--AGG.-UCUucUCCU.--GGUCCC
NC_027871.1/38657648-38657707        GGGAUC.ACUGUU-GGCCUCA-UGC.......................U-CUGGGGCCUAGCUUAAUAC--AGG.-UCUucUCCU.--GGUCCC
NW_012106419.1/1059589-1059515       AGGGCU.U-UGCAAAACCUUGAUUUgccc...............cgguUUCCAAGGUUUUGUUUUUGACCUUGAgUUCU.uUCCA.GCGGCCCU
NW_014804636.1/804090-804016         AGGGCU.U-UGCAAAACCUUGAUUUgccc...............cgguUUCCAAGGUUUUGUUUUUGACAUUGAgUUCU.uUCCA.GUGGCCCU
NW_005092962.1/26663462-26663536     AGGGCU.U-UGCAAAACCUUGAUUUgccc...............cgguUUCCAAGGUUUUGUUUUUGACAUUGAgUUCU.uUCCA.GUGGCCCU
NW_012018354.1/8607012-8607086       AGGGCU.U-UGCAAAACCUUGAUUUgccc...............cgguUUCCAAGGUUUUGUUUUUGACAUUGAgUUCU.uUCCA.GUGGCCCU
NC_027894.1/104250036-104249962      AGGGCU.U-UGCAAAACCUUGAUUUgccc...............cgguUUCCAAGGUUUUGUUUUUGACAUUGAgUUCU.uUCCA.GUGGCCCU
NC_022273.1/93382205-93382279        AGGGCU.U-UGCAAAACCUUGAUUUgccc...............cgguUUCCAAGGUUUUGUUUUUGACAUUGAgUUCU.uUCCA.GUGGCCCU
NW_018334970.1/82745-82833           AGGGCU.U-UGCAAAGCCUUGAUUUgcagaaccucaguccacccugguUUUGAGGGUUUUGCUUUGACAGCAGG.UUCU..GCUUaGAGGCCCU
NW_016814090.1/986753-986679         AGGGCU.U-UGCAAAACCUUGAUUUgccc...............cgguUUCCAAGGUUUUGUUUUUGACACUGAgGUCU.uUCCA.GCGGCCCU
NW_019317900.1/203781-203855         AGGGCU.U-UGCAAAACCUUGAUUUgccc...............cgguUUCCAAGGUUUUGUUUUUGACACUGAgGUCU.uUCCA.GCGGCCCU
NW_010813132.1/38368-38442           AGGGCU.U-UGCAAAACCUUGAUUUgccc...............cgguUUCCAAGGUUUUGUUUUUGACACUGAgGUCU.uUCCA.GCGGCCCU
#=GC SS_cons                         ((((((.,,,,<<<-<<<<<<<_____......................___>>>>>>>->>>,,,,,,,,,<<<._____.._>>>.,,))))))
#=GC RF                              AGGGCc.aCUGCcAGGCCUuGAUgu.......................UuCCaGGGCCUgGCUUaguaCucaGG.UUCU..gCCu.gcgGCCCU
//
```

e *ANTXR2-33250*

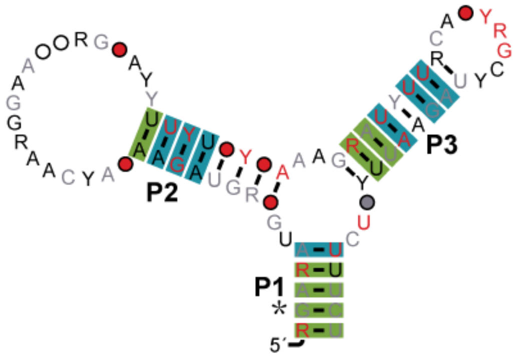

Sequence alignment for the motif *ANTXR2-33250*

```
# STOCKHOLM 1.0
NW_014013898.1/16980943-16981018        GGAAAUGUUGUAGAAAA..........CCAAGGGAAGAGGAAUUUUCUACGA...AAGAAUUUUACAUCAGC.UUAGAAUUUAUC..........UUUCC
NW_019932803.1/40061149-40061224        GGAAAUGUUGUAGAAAA..........CCAAGGGAAGAGGAAUUUUCUACGA...AAGAAUUUUACAUCAGC.UUAGAAUUUAUC..........UUUCC
NC_036883.1/49163124-49163199           GGAAAUGUUGUAGAAAA..........CCAAGGGAAGAGGAAUUUUCUACGA...AAGAAUUUUACAUCAGC.UUAGAAUUUAUC..........UUUCC
NC_000004.12/80069840-80069765          GGAAAUGUUGUAGAAAA..........CCAAGGGAAGAGGAAUUUUCUACGA...AAGAAUUUUACAUCAGC.UUAGAAUUUAUC..........UUUCC
NT_016354.20/21148459-21148384          GGAAAUGUUGUAGAAAA..........CCAAGGGAAGAGGAAUUUUCUACGA...AAGAAUUUUACAUCAGC.UUAGAAUUUAUC..........UUUCC
NG_015987.1/8484-8559                   GGAAAUGUUGUAGAAAA..........CCAAGGGAAGAGGAAUUUUCUACGA...AAGAAUUUUACAUCAGC.UUAGAAUUUAUC..........UUUCC
NC_027872.1/49975946-49976021           GGAAAUGUUGUAGAAAA..........CCAAGGGAAGAGGAAUUUUCUACGA...AAGAAUUUUACAUCAGC.UUAGAAUUUAUC..........UUUCC
NC_018428.2/91027944-91027869           GGAAAUGUUGUAGAAAA..........CCAAGGGAAGAGGAAUUUUCUACGA...AAGAAUUUUACAUCAGC.UUAGAAUUUAUC..........UUUCC
NW_017493685.1/11028574-11028499        GGAAAUGUUGUAGAAAA..........CCAAGGGAAGAGGAAUUUUCUACGA...AAGAAUUUUACAUCAGC.UUAGAAUUUAUC..........UUUCC
NC_036907.1/78277831-78277756           GGAAAUGUUGUAGAACA..........CCAAGGGAAGAGGAAUUUUCUAUGA...AAGAAUUUUACAUCAGC.UUAGAAUUUAUC..........UUUCC
NW_019937216.1/12474055-12473980        GGAAAUGUUGUAGAACA..........CCAAGGGAAGAGGAAUUUUCUAUGA...AAGAAUUUUACAUCAGC.UUAGAAUUUAUC..........UUUCC
NW_003501380.2/27719376-27719301        GGAAAUGUUGUGGAACA..........CCAAGGGAAGAGGAAUUUUCUACGA...AAGAAUUUUACAUCAGC.UUAGAAUUUAUC..........UUUCC
NC_019824.1/64968155-64968080           GGAAAUGUUGUGGAACA..........CCAAGGGAAGAGGAAUUUUCUACGA...AAGAAUUUUACAUCAGC.UUAGAAUUUAUC..........UUUCC
NW_020251508.1/61929579-61929654        GGAAAUGUUAUAGAACA..........CCAAGGGAAGAGGAAUUUUCUAUGA...AAGAAUUUUACAUCAGC.AUAGAAUUUAUC..........UUUCC
NC_037672.1/61929579-61929654           GGAAAUGUUAUAGAACA..........CCAAGGGAAGAGGAAUUUUCUAUGA...AAGAAUUUUACAUCAGC.AUAGAAUUUAUC..........UUUCC
NW_018490587.1/3893892-3893967          GGAAAUGAUGUAGAACA..........CCAAGGGAAGAGGAAUUUUCUACAA...AAGAAUUUUACGUCAGC.UUAGAAUUUAUC..........UUUCC
NW_012115156.1/2015278-2015203          GGAAAUGUUGUAGAACA..........CCAAGGGAAGAGGAAUUUUCUAUGA...AAGAAUUUUACAUCAGC.AUAGAAUUUAUC..........UUUCC
NW_014804880.1/848809-848884            GGAAAUGUUGUAGAACA..........CCAAGGGAAGAGGAAUUUUCUAUGA...AAGAAUUUUACAUCAGC.AUAGAAUUUAUC..........UUUCC
NW_005092968.1/50376454-50376529        GGAAAUGUUGUAGAACA..........CCAAGGGAAGAGGAAUUUUCUAUGA...AAGAAUUUUACAUCAGC.AUAGAAUUUAUC..........UUUCC
NW_018755196.1/255582-255657            GGAAAUGUUGUAGAACA..........CCAAGGGAAGAGGAAUUUUCUAUGA...AAGAAUUUUACAUCAGC.AUAGAAUUUAUC..........UUUCC
NW_012004825.1/13015371-13015446        GGAAAUGUUGUAGAACA..........CCAAGGGAAGAGGAAUUUUCUAUGA...AAGAAUUUUACAUCAGC.AUAGAAUUUAUC..........UUUCC
NW_012012411.1/746822-746747            GGAAAUGUUGUAGAACA..........CCAAGGGAAGAGGAAUUUUCUAUGA...AAGAAUUUUACAUCAGC.AUAGAAUUUAUC..........UUUCC
NC_027897.1/54388036-54388111           GGAAAUGUUGUAGAACA..........CCAAGGGAAGAGGAAUUUUCUAUGA...AAGAAUUUUACAUCAGC.AUAGAAUUUAUC..........UUUCC
NC_018156.2/61174605-61174680           GGAAAUGUUGUAGAACA..........CCAAGGGAAGAGGAAUUUUCUAUGA...AAGAAUUUUACAUCAGC.AUAGAAUUUAUC..........UUUCC
NC_022276.1/54434088-54434163           GGAAAUGUUGUAGAACA..........CCAAGGGAAGAGGAAUUUUCUAUGA...AAGAAUUUUACAUCAGC.AUAGAAUUUAUC..........UUUCC
NW_016107379.1/113028-112953            GGAAAUGAUGUAGAACA..........CCAAAGAAAGAGGAAUUUUCUACGA...AAGAAUUUUACGUCAGC.UUAGAAUUUAUC..........UUUCC
NW_006772469.1/3132197-3132272          GGAGAUGAGAUAGAACA..........UCAAAGGAAAAAGCACUUUCUACCA...AAAAAUUUUCCAUUGGC.CUAGAAUUCAUC..........UUUCU
NW_012108141.1/6319191-6319116          GGAAAUGUUGUAGAACA..........CCAAGGGAAGAGGAAUUUUCUAUGA...AAGAAUUUUAUAUCAGC.AUAGAAUUUAUC..........UUUCC
NW_010829553.1/1532286-1532361          GGAAAUGUUGUAGAACA..........CCAAGGGAAGAGAAAUUUUCUAUGA...AAGAAUUUUACAUCAGC.AUAGAAUUUAUC..........UUUCC
NC_023648.1/28496122-28496047           GGAAAUAUUGUAGAACA..........CCAAGGGAAGAGGAAUUUUCUAUGA...AAGAAUUUUACAUCAGC.AUAGAAUUUAUC..........UUUCC
NW_006735246.1/10585168-10585093        GGAAAUAUUGUAGAACA..........CCAAGGGAAGAGGAAUUUUCUAUGA...AAGAAUUUUACAUCAGC.AUAGAAUUUAUC..........UUUCC
NW_003943613.1/3700126-3700051          GGAAAUGAUGUAGAACA..........CCAAGGGAAGAGGAGUUUUCUACAA...AAGAAUUUUACGUCAGC.UUAGAAUUUAUC..........UUUCC
NW_019317461.1/5228786-5228861          GGAAAUGUUGUAGAACA..........CCAAGGGAAGAGGAAUUUUCUAUGA...AAGAAUUUUACAUCAGC.AUAGAAUUGAUC..........UUUCC
NW_019160918.1/8042930-8042855          GGAGAUGAGAUAGAACA..........UCAAAGGAAGAAGCACUUUCUACUA...AAAAAUUUUCCAUUGGC.CUAGAAUUCAUC..........UUUCU
NW_020174050.1/7691501-7691426          GGAGAUGAGAUAGAACA..........UCAAAGGAAGAAGCACUUUCUACUA...AAAAAUUUUCCAUUGGC.CUAGAAUUCAUC..........UUUCU
NC_013898.1/114468923-114468998         GGAAAUGAUGUAGAACA..........CCAAGGGAAGAGGAAUUUUCUACUA...AAGAAUUUUAUGUCAGC.UUAGAAUUUAUC..........UUUCC
NW_003183267.1/2726195-2726270          GGAAAUGAUGUAGAACA..........CCAAGGGAAGAGGAAUUUUCUACUA...AAGAAUUUUAUGUCAGC.UUAGAAUUUAUC..........UUUCC
NW_019873609.1/8339201-8339126          GGAGAUGAGAUAGGACA..........UCAAAGGAAGAAGCACUUUCUACCA...AAAAAUUUUCCAUUGGC.CUAGAAUUCAUC..........UUUCU
NW_017843021.1/7387021-7386946          GGAGAUGAGAUAGAACA..........UCAAAGGAAGAAGCACUUUCUACCA...AAAAAUUUUCCAUUGGC.CUACAAUUCAUC..........UUUCU
NW_006725343.1/4174987-4174912          GGAGAUGAGAUAGAACA..........UCAAAGGAAGAAGCACUUUCUACAA...AAGAGUUUUCCAUUGGC.CUAGAAUUCAUC..........UUUCU
NW_016806447.1/295549-295624            CGAAAUGUUGUAGAACA..........CCAAGGGAAGAGAAAUUUUCUAUGA...AAGAAUUUUACAUCAGC.AUAGAAUUUAUC..........UUUCC
NW_007739406.1/16708-16783              GGAGAUGAAAUAGAAUA..........CAAAAGGGAGGAGCAUUUUCUAUGA...AAGAAUUUUACAUCAGC.CUAGAAUUUAUC..........UUUCC
NW_004438535.1/1747670-1747745          GGAGAUGAGAUAGAACA..........UCAAAGGAAGAAGCGCUUUCUACCA...AAAAAUUUUCCAUUGGC.CUACAGUUCAUC..........UUUCU
NW_020356462.1/10403072-10403147        GGGGGUGAGAUAGAAUA..........CCAAGGGAAGAAGUAUUUUCUACAA...AAGGAUUUUACAUUGGC.CUAGAAUUCAUC..........UUUCU
NC_034574.1/91001811-91001730           GAGAA-GUAGGAGAAGA..........UCAAAGGAA--GGAACAUUUUUCUAggaAAGAGUCUUGCAGCAGCcUAGAGACUU-UCcag....uuaUUCUU
NW_018388417.1/91001811-91001730        GAGAA-GUAGGAGAAGA..........UCAAAGGAA--GGAACAUUUUUCUAggaAAGAGUCUUGCAGCAGCcUAGAGACUU-UCcag....uuaUUCUU
NC_034602.1/86485695-86485614           GAGAA-GUAGGAGAAGA..........UCAAAGGAA--GGAAUAUUUUUCUAggaAAGAGUCUUGCAGCAGCcUAGAGACUU-UCcag....uuaUUCUU
NW_018391587.1/86485695-86485614        GAGAA-GUAGGAGAAGA..........UCAAAGGAA--GGAAUAUUUUUCUAggaAAGAGUCUUGCAGCAGCcUAGAGACUU-UCcag....uuaUUCUU
NT_109320.5/21929019-21928938           GAGAA-GUAGGAGAAGG..........UCAAAGGAA--GGAACAUUUUUCUAggaAAGAGUCUUGCAGCAGCcUAGAGACU-GUCcag....uuaUUCUU
NC_000071.6/98027873-98027792           GAGAA-GUAGGAGAAGG..........UCAAAGGAA--GGAACAUUUUUCUAggaAAGAGUCUUGCAGCAGCcUAGAGACU-GUCcag....uuaUUCUU
NW_017619847.1/15955334-15955407        GGAGAUGAGGUAGAA--..........CCAAGGGAAGAAGUGUUUUCUACAA...AAGGAUUUUACAUUGGC.CUAGAAUUUAUC..........UUUCU
NW_006711583.1/15636894-15636967        GGAGAUGAGGUAGAA--..........CCAAGGGAAGAAGUGUUUUCUACAA...AAGGAUUUUACAUUGGC.CUAGAAUUUAUC..........UUUCU
NW_015131058.1/1127164-1127237          GGAGAUGAGGUAGAA--..........CCAAGGGAAGAAGUGUUUUCUACAA...AAGGAUUUUACAUUGGC.CUAGAAUUUAUC..........UUUCU
NC_018726.3/142373795-142373868         GGAGAUGAGGUAGAA--..........CCAAGGGAAGAAGUGUUUUCUACAA...AAGGAUUUUACAUUGGC.CUAGAAUUUAUC..........UUUCU
NW_019365218.1/102079316-102079389      GGAGAUGAGGUAGAA--..........CCAAGGGAAGAAGUGUUUUCUACAA...AAGGAUUUUACAUUGGC.CUAGAAUUUAUC..........UUUCU
NW_003726116.1/4299213-4299138          GGAGGUGAGAUAGAAUA..........CCAAGGGAAGAAGUAUUUUCUACAA...AAGGAUUUUACAUUGGC.CUACAAUUCAUC..........UUUCU
NC_006614.3/4299313-4299238             GGAGGUGAGAUAGAAUA..........CCAAGGGAAGAAGUAUUUUCUACAA...AAGGAUUUUACAUUGGC.CUACAAUUCAUC..........UUUCU
NW_020267356.1/22397467-22397542        GGAGGUGAGAUAGAAUA..........CCAAGGGAAGAAGUAUUUUCUACAA...AAGGAUUUUACAUUGGC.CUACAAUUCAUC..........UUUCU
NW_004454188.1/4380194-4380119          GGAGAUGAGGUGGAACA..........CCAAGGGAAGAGGCAUUCUCUAUAA...AAGAAUUUUACGUUGGC.CUAGAAUUUAUC..........UUUCU
NW_007256044.1/157928-157854            GGAGAUGAGGUAGAACA..........CCAAAGGAAGAAGCAUUUUCCAUGA...AAGAAUUUUG-AUCAGC.CUAGAAUUUAUC..........UUUCC
NW_020339223.1/66572324-66572253        GGAGAUG--GUAGAA--..........CCAAGGGAAGAAGUGUUUUCUACAA...AAGGAUUUUACAUUGGC.CUAGAAUUUAUC..........UUUCU
NC_010450.4/137731851-137731926         GGAGAUAAAGGAGAACG..........CCAAAGGAAGAAGCACCUUCUACAA...GAGAAUUUUACAUUGGC.CUGGAAUUCGUG..........UUGCU
NW_018084687.1/83076457-83076532        GGAGAUAAAGGAGAACG..........CCAAAGGAAGAAGCACCUUCUACAA...GAGAAUUUUACAUUGGC.CUGGAAUUCGUG..........UUGCU
NW_020312926.1/64309768-64309683        GGAGAUGAGGUAGAACGgggaaaauagGUAAAGGAAGAAGUAUUUUCUACGA...AAGGAUUUUACAUUAGC.CUAGAAUUCAUC..........UUUCU
NW_014637047.1/2750981-2750908          GGAGAUGAGGUAGACCA..........CCAAGGAA---GAAGUGUUUUCUA..uGAGGAUUUUACAUUGGU.CUAGAAUUUAUC..........UUUCU
```

f *ICE2-92051*

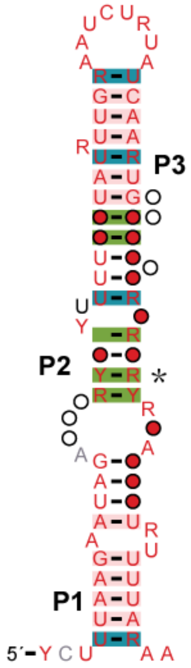

Sequence alignment for the motif *ICE2-92051*

```
# STOCKHOLM 1.0
NW_004438478.1/7139840-7139760             UUUUUAAGAAUAGGCAA.GCUUCuUUUUGU.UAUaUUGGAAUcuaUAUCAAGUG--AUAAUGUAAGUGCAu..aGACUAUUUUAGAA
NW_005355159.1/4858261-4858341             CCUUUAAGAAUAGACAG.GCUUC.UUUUGuUAUgUUGGAAUcugUAUCAAGUGCUCAUGUAAAUGCAUA....GACUGUUUUAGAA
NW_005871252.1/1780385-1780465             CCUUUAAGAAUAGACAA.GCUUC.UUUUGuUAUgUUGGAAUcugUAUCAAGUGCUCAUGUAAAUGCAUA....GACUGUUUUAGAA
NC_036894.1/38933522-38933446              UCUUUAAGAAUAGA---cAUGCU.UUUUGU.UAUgUUGAAAUcuaUAUCAAAUGAUAUA-AAUGCAUAGA....CUGUAUUUUAAAA
NC_027883.1/57752752-57752676              UCUUUAAGAAUAGA---cAUGCU.UUUUGU.UAUgUUGAAAUcuaUAUCAAAUGAUAUA-AAUGCAUAGA....CUGUAUUUUAAAA
NW_014014243.1/10012731-10012655           UCUUUAAGAAUAGA---cAUGCU.UUUUGU.UAUgUUGAAAUcuaUAUCAAAUGAUAUA-AAUGCAUAGA....CUGUAUUUUAAAA
NW_019932849.1/29966286-29966210           UCUUUAAGAAUAGA---cAUGCU.UUUUGU.UAUgUUGAAAUcuaUAUCAAAUGAUAUA-AAUGCAUAGA....CUGUAUUUUAAAA
NC_000015.10/60477594-60477518             UCUUUAAGAAUAGA---cAUGCU.UUUUGU.UAUaUUGAAAUcuaUAUCAAAUGAUAUA-AAUGCAUAGA....CUGUAUUUUAAAA
NT_010194.18/37200720-37200644             UCUUUAAGAAUAGA---cAUGCU.UUUUGU.UAUaUUGAAAUcuaUAUCAAAUGAUAUA-AAUGCAUAGA....CUGUAUUUUAAAA
NW_017493705.1/38904474-38904398           UCUUUAAGAAUAGA---cAUGCU.UUUUGU.UAUaUUGAAAUcuaUAUCAAAUGAUAUA-AAUGCAUAGA....CUGUAUUUUAAAA
NW_017493705.1/38913982-38914058           UCUUUAAGAAUAGA---cAUGCU.UUUUGU.UAUaUUGAAAUcuaUAUCAAAUGAUAUA-AAUGCAUAGA....CUGUAUUUUAAAA
NC_018439.2/38904474-38904398              UCUUUAAGAAUAGA---cAUGCU.UUUUGU.UAUaUUGAAAUcuaUAUCAAAUGAUAUA-AAUGCAUAGA....CUGUAUUUUAAAA
NC_018439.2/38913982-38914058              UCUUUAAGAAUAGA---cAUGCU.UUUUGU.UAUaUUGAAAUcuaUAUCAAAUGAUAUA-AAUGCAUAGA....CUGUAUUUUAAAA
NG_054881.1/6567-6643                      UCUUUAAGAAUAGA---cAUGCU.UUUUGU.UAUaUUGAAAUcuaUAUCAAAUGAUAUA-AAUGCAUAGA....CUGUAUUUUAAAA
NW_004087887.1/36962125-36962050           UCUUUAAGAAUAGA---cAUGCU.-UUUGU.UAUaUUGAAAUcuaUAUCAAAUGAUAUA-AAUGCAUAGA....CUGUAUUUUAAAA
NC_019821.1/79711168-79711093              UCUUUAAGAAUAGA---cAUGCU.-UUUGU.UAUaUUGAAAUcuaUAUCAAAUGAUAUA-AAUGCAUAGA....CUGUAUUUUAAAA
NW_014636793.1/3714000-3714082             UCUUUAAGAAUAGACAA.GUUUCcUUUUGU.UAUaUUGAAAUcugUAUCAAGUG--AUAAUGUAAAUGCAuagaUUAUAUUUUAGAA
NW_007673186.1/980658-980740               UCUUUAAGAAUAGACAA.GUUUCcUUUUGU.UAUaUUGAAAUcugUAUCAAGUG--AUAAUGUAAAUGCAuagaUUAUAUUUUAGAA
NW_019641712.1/132601286-132601368         UCUUUAAGAAUAGACAA.GUUUCcUUUUGU.UAUaUUGAAAUcugUAUCAAGUG--AUAAUGUAAAUGCAuagaUUAUAUUUUAGAA
NC_009144.3/132601286-132601368            UCUUUAAGAAUAGACAA.GUUUCcUUUUGU.UAUaUUGAAAUcugUAUCAAGUG--AUAAUGUAAAUGCAuagaUUAUAUUUUAGAA
#=GC SS_cons                               :::<<<<<-<<<<----.<<<<-.-<<<<<.<<<.<<<<___...__>>>>>>>-->>>->>->>>>---....>>>>-->>>>>::
#=GC RF                                    UgUcUAGGAAaggGgAg.gUgug.Uggucu.uau.UugaAaU...UAucaAaugaUagaAccAacAuGca....ucuUAuUCUAgaA
//
```

g *Motif-59232*

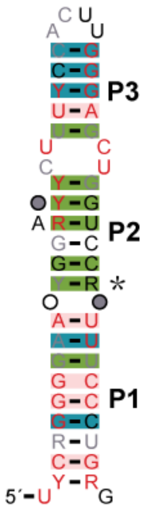

Sequence alignment for *Motif-59232*

```
# STOCKHOLM 1.0
NC_000017.11/49976559-49976613        UUCA.GG.GGA.A.aU.GGGAACCu.cUUUUCCAUUUGGGAGCUGGCCC.AgUUUCCCUGAG
NC_036896.1/43218793-43218847         UUCA.GG.GGA.A.aU.GGGAACCu.cUUUUCCAUUUGGGAGCUGGCCC.AgUUUCCCUGAG
NT_010783.16/23040579-23040633        UUCA.GG.GGA.A.aU.GGGAACCu.cUUUUCCAUUUGGGAGCUGGCCC.AgUUUCCCUGAG
NG_030592.1/12362-12416               UUCA.GG.GGA.A.aU.GGGAACCu.cUUUUCCAUUUGGGAGCUGGCCC.AgUUUCCCUGAG
NW_019932861.1/10125594-10125540      UUCA.GG.GGA.A.aU.GGGAACCu.cUUUUCCAUUUGGGAGCUGGCCC.AgUUUCCCUGAG
NC_018429.2/33491538-33491484         UUCA.GG.GGA.A.aU.GGGAGCCu.cUUUUCCAUUUGGGAGCUGGCCC.AgUUUCCCUGAG
NW_017493686.1/33491538-33491484      UUCA.GG.GGA.A.aU.GGGAGCCu.cUUUUCCAUUUGGGAGCUGGCCC.AgUUUCCCUGAG
NW_012118488.1/4032322-4032376        UUCA.GG.GGA.A.aU.GGGAAUCu.cUUUCCCAUUUGGGAGCUGGUCC.AgUUUCCCUGAG
NW_016815820.1/1329868-1329814        UUCA.GG.GGA.A.aU.GGAAAUCu.cUUUUCCAUUUGGGAGCUGGUCC.AgUUUCCCUGAG
NW_010827790.1/6725-6779              UUCA.GG.GGA.A.aU.GGAAAUCu.cUUUUCCAUUUGGGAGCUGGUCC.AgUUUCCCUGAG
NW_019317027.1/7511208-7511262        UUCA.GG.GGA.A.aU.GGAAAUCu.cUUUUCCAUUUGGGAGCUGGUCC.AgUUUCCCUGAG
NC_036920.1/43260035-43259981         UUCA.GG.GGA.A.aU.GGGAACCu.cUUUUCCAUUUGGGAGCUGGUCC.AgUUUCCCUGAG
NW_019937247.1/32653973-32654027      UUCA.GG.GGA.A.aU.GGGAACCu.cUUUUCCAUUUGGGAGCUGGUCC.AgUUUCCCUGAG
NW_003501553.1/2495742-2495796        UUCA.GG.GGA.A.aU.GGGAACCu.cUUUUCCAUUUGGGAGCUGGUCC.AgUUUCCCUGAG
NW_018508337.1/445075-445129          UUCA.GG.GGA.A.aU.GGGAAUCu.cUUUUCCAUUUGGGAGCUGGUCC.AgUUUCCCUGGG
NW_003943648.1/3640104-3640158        UUCA.GG.GGA.A.aU.GGGAAUCu.cUCUUCCAUUUGGGAGCUGGUCC.AgUUUCCCUGAG
NW_005360086.1/1093193-1093246        UCCA.GG.GGG.A.aG.GAGAGUC..uUUUCCUACUUGGGAGCUGGUUC.CgUUUCCCUGAG
NC_033675.1/25273000-25272946         UUCA.GGgGAA.A.cC.GGGAUCU..cUCUUCCACUUGGGAGCUGGUCC.GcUUUCCCUGGG
NW_017963770.1/25273000-25272946      UUCA.GGgGAA.A.cC.GGGAUCU..cUCUUCCACUUGGGAGCUGGUCC.GcUUUCCCUGGG
NW_016107438.1/3155259-3155313        UUCA.GG.GGA.A.aU.GGGAAUUu.cUUUUCCAUUUGGGAGCUGGUCC.AgUUUCCCUGAG
NC_013900.1/107556158-107556104       UUCA.GG.GGA.A.aUgGGAAUCU..cUUUUCCAUUUGGGAGCUAGUCC.AgUUUCCCUGAG
NW_003183523.1/187181-187127          UUCA.GG.GGA.A.aUgGGAAUCU..cUUUUCCAUUUGGGAGCUAGUCC.AgUUUCCCUGAG
NC_023657.1/43359328-43359274         UUCA.GG.GGA.A.aU.GGGAAUCu.cUUUUCCAUUCGGGAGCUGGUCC.AgUUUCCCUGAG
NW_006735264.1/22703311-22703257      UUCA.GG.GGA.A.aU.GGGAAUCu.cUUUUCCAUUCGGGAGCUGGUCC.AgUUUCCCUGAG
NW_005883079.1/395353-395406          UCCA.GG.GGA.A.aU.GGGAAUC..cUUUCCCACUUGGGAGCUGGUCC.AgUUUCCCAGGG
NW_012135969.1/7391666-7391720        UUCA.GG.GGA.A.aC.GGGGAUCu.cUCUUCCACUUGGGAGCUGGUCC.GcUUUCCCUGGG
NW_017739081.1/2386278-2386331        UCCA.GG.GGG.A.aU.GGGAGUC..cUUUUCCACUUGGGAGCUGGUCC.AgUUGCCCUGAG
NW_005872371.1/68741-68794            UCCA.GG.GGG.A.aG.GAGAGUC..uUUUCCUCCUUGGGAGCUGGUUC.CcUUUCCCUGAG
NW_014014319.1/9954288-9954234        UUCA.GG.GGA.A.aU.GGGAACCu.cUUUUCCAUUUCGGAGCUGGCCC.AgUUUCCCUGAG
NC_027885.1/48937046-48937100         UUCA.GG.GGA.A.aU.GGGAACCu.cUUUUCCAUUUCGGAGCUGGCCC.AgUUUCCCUGAG
NW_012006043.1/786592-786538          UUCA.GGaGAA.A..UgGGAA-UCu.cUUUUCCAUUUGGGAGCUGGUCC.AgUUUCCCUGAG
NW_018759295.1/157983-158037          UUCA.GGaGAA.A..UgGGAA-UCu.cUUUUCCAUUUGGGAGCUGGUCC.AgUUUCCCUGAG
NW_012014910.1/8123035-8123089        UUCA.GGaGAA.A..UgGGAA-UCu.cUUUUCCAUUUGGGAGCUGGUCC.AgUUUCCCUGAG
NC_018167.2/31615395-31615449         UUCA.GGaGAA.A..UgGGAA-UCu.cUUUUCCAUUUGGGAGCUGGUCC.AgUUUCCCUGAG
NC_037684.1/23751525-23751471         UUCA.GGaGAA.A..UgGGAA-UCu.cUUUUCCAUUUGGGAGCUGGUCC.AgUUUCCCUGAG
NW_020251520.1/23751525-23751471      UUCA.GGaGAA.A..UgGGAA-UCu.cUUUUCCAUUUGGGAGCUGGUCC.AgUUUCCCUGAG
NW_017871107.1/657879-657933          UCCAaGG.GAA.A..CaGGAAUCU..cUUUUCUACUUGGGAGCUGGUCCaG.UUUCCCUGGG
NC_009154.3/25813844-25813897         UCCG.GG.GGAgA..C.GGGAAUC..cUUUUCCACUUGGGAGCUGGCCCaG.UUUCCCUGGG
NW_007673577.1/136105-136052          UCCG.GG.GGAgA..C.GGGAAUC..cUUUUCCACUUGGGAGCUGGCCCaG.UUUCCCUGGG
NW_019641722.1/25813844-25813897      UCCG.GG.GGAgA..C.GGGAAUC..cUUUUCCACUUGGGAGCUGGCCCaG.UUUCCCUGGG
NW_014638702.1/6425186-6425133        UCCG.GG.GGAgA..C.GGGAAUC..cUUUUCCACUUGGGAGCUGGCCCaG.UUUCCCUGGG
NW_006210352.1/250857-250804          UCCA.GG.GGA.AaaG.GGAAUCC...UUUCCCACUUGGGAGCUGGUCC.AgUUUCCCAGGG
NW_011510164.1/315290-315237          UCCA.GG.GGA.AaaG.GGAAUCC...UUUCCCACUUGGGAGCUGGUCC.AgUUUCCCAGGG
NW_011592891.1/221110-221057          UCCA.GG.GGA.AaaG.GGAAUCC...UUUCCCACUUGGGAGCUGGUCC.AgUUUCCCAGGG
NW_006298628.1/1756976-1756923        UCCA.GG.GGA.A.aG.GAGAGUC..uUUUCCUCCUUGGGAGCUGGUCC.CgUUUCCCUGAG
NW_015494661.1/1055757-1055810        UCCA.GGgGGA.A..UgAGAA-UU..cUUUUCCACUUGGGAGCUAGUCUaG.UUCCCCUGAG
NW_006443570.1/5243056-5243109        UCCG.GG.GGG.A.aU.GAGAAUC..cUUUUCCACUUGGGAGCUGAUCC.AgUUUCCCUGAG
NW_012106363.1/3810412-3810358        UUCA.GGaGAA.A..UgGGAA-UCu.cUUUUCCAUUUGGGAGCUGGUCC.AgUUUCCCUGAU
NW_004936490.1/11643259-11643204      UUCCaGGgGAA.A..CaGGAAUCU..cUUUUCCACUUGGGAUCUGGUCCaG.UUUCCCGGGG
NW_015351252.1/11838915-11838859      UUCCaGGgGAA.A..CaGGAAUCUc.uUUUCCCACUUGGGAUCUGGUCCaG.UUUCCCGGGG
NW_004458100.1/548546-548491          UCCU.GG.GGA.A.aU.GGGAAUCcggUUUUCCGCUUGGGAACUGGUCC.AgUUUCCCUGGG
NW_007370743.1/2038215-2038161        UCCA.GGgGGG.A.aG.GGGAGCC..cUUUCCUACUGGGGAGCUGGUUC.AgUUUCCCUGAG
NW_017731390.1/1348265-1348212        UCCA.GG.GGG.A.aU.GCAAGUC..cUUUUCCACCUGGGAGCUGGUCC.AgUUGCCCUGAG
NW_011888885.1/5514835-5514781        UCCG.GGgGGG.A.aU.GAGAAUC..cUUUUUCACUUGGGAGCUGAUCC.AgUUUCCCUGAG
NW_004558727.1/23889139-23889083      UUCCuGG.GGA.A.aCaGGAAUUC..cUUUUUCACUUGGGAGCUGGUCCaGgUUUCCUGGGC
#=GC SS_cons                          :<<<.<<.<<<.<..<.<<<--<<...-<<<<<____>>>>>-->>>>>.>.>>>>>>>>>:
#=GC RF                               UcCa.GG.GgA.A..g.GgGAucc...UuuccCAcUUGggagCUggCcC.c.UUuCCCuGaG
//
```

h *DPH1-85951*

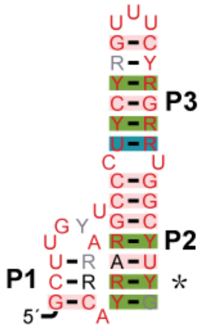

Sequence alignment for the motif *DPH1-85951*

```
# STOCKHOLM 1.0
NW_019160909.1/6848701-6848743       GCUUGUAUGCACAAGGUCCCUCCCAGUUUCUGGGAUGGCCU.UG
NW_004438434.1/15330817-15330775     GCUUGUAUGCACAAGGUCCCUCCCAGUUUCUGGGAUGGCCU.UG
NW_006780838.1/1598955-1598913       GCUUGUAUGCACAAGGUCCCUCCCAGUUUCUGGGAUGGCCU.UG
NW_006443570.1/22537327-22537369     GCUUGCAGGCAUGAGGUCCCUCCUGGUUUCUGGGAUGGCCU.CA
NW_005354039.1/829077-829035         GCUUGCAGGCAUGAGGUCCCUCCUGGUUUCUGGGAUGGCCU.CA
NW_007370694.1/11264969-11265011     GCUUGCAGGCAUGAGGUCCCUCCUGGUUUCUGGGAUGGCCU.CA
NW_006294069.1/3546906-3546948       GCUUGCAGGCAUGAGGUCCCUCCUGGUUUCUGGGAUGGCCU.CA
NW_004454199.1/15941204-15941162     GCUUGCAGGCAUGAGGUCCCUCCCAGUUUCUGGGAUGGCCU.CG
NW_018658080.1/225484-225526         GCUUGCAAGCACAAAGUCCCUUCUGGUUUCCAGAGUGGCUU.UG
NW_019873574.1/13537894-13537852     GCUUGUAUGCACAAGGUCCCUCCCCGUUUCUGGGAUGGCCU.UG
NC_000017.11/2036184-2036227         GCUUGGAGACACAAGGUCCCUCCUGGUUUCUGGGGUGGCCUcUG
NC_036896.1/1717570-1717613          GCUUGGAGACACAAGGUCCCUCCUGGUUUCUGGGGUGGCCUcUG
NW_014014291.1/1836725-1836768       GCUUGGAGACACAAGGUCCCUCCUGGUUUCUGGGGUGGCCUcUG
NT_010718.17/1547197-1547240         GCUUGGAGACACAAGGUCCCUCCUGGUUUCUGGGGUGGCCUcUG
NC_027885.1/1915687-1915730          GCUUGGAGACACAAGGUCCCUCCUGGUUUCUGGGGUGGCCUcUG
NG_051946.1/11073-11116              GCUUGGAGACACAAGGUCCCUCCUGGUUUCUGGGGUGGCCUcUG
NW_019932856.1/1717570-1717613       GCUUGGAGACACAAGGUCCCUCCUGGUUUCUGGGGUGGCCUcUG
NW_005871310.1/716067-716025         GCUUGCAGGCAUGCGGUCCCUCCUGGUUUCUGGGAUGGCCU.CA
NW_015504493.1/609034-609076         GCUUGCAGCCAUGAGGUCCCUCCUGGUUUCUGGGAUGGCCU.CA
#=GC SS_cons                         <<<____>>>-<<<<<-<<-<<<<<<___>>>>>>->>>>>.>>
#=GC RF                              GccUGaAggCACaaGGUCCCuCCcaGUUcCugGGaUGGCCu.uG
//
```

i *ARNTL-1638-long-stem*

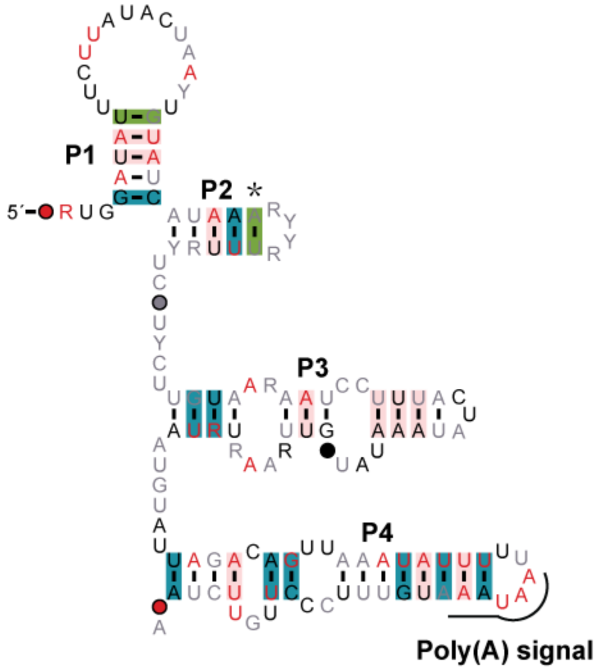

Sequence alignment for the motif *ARNTL-1638*

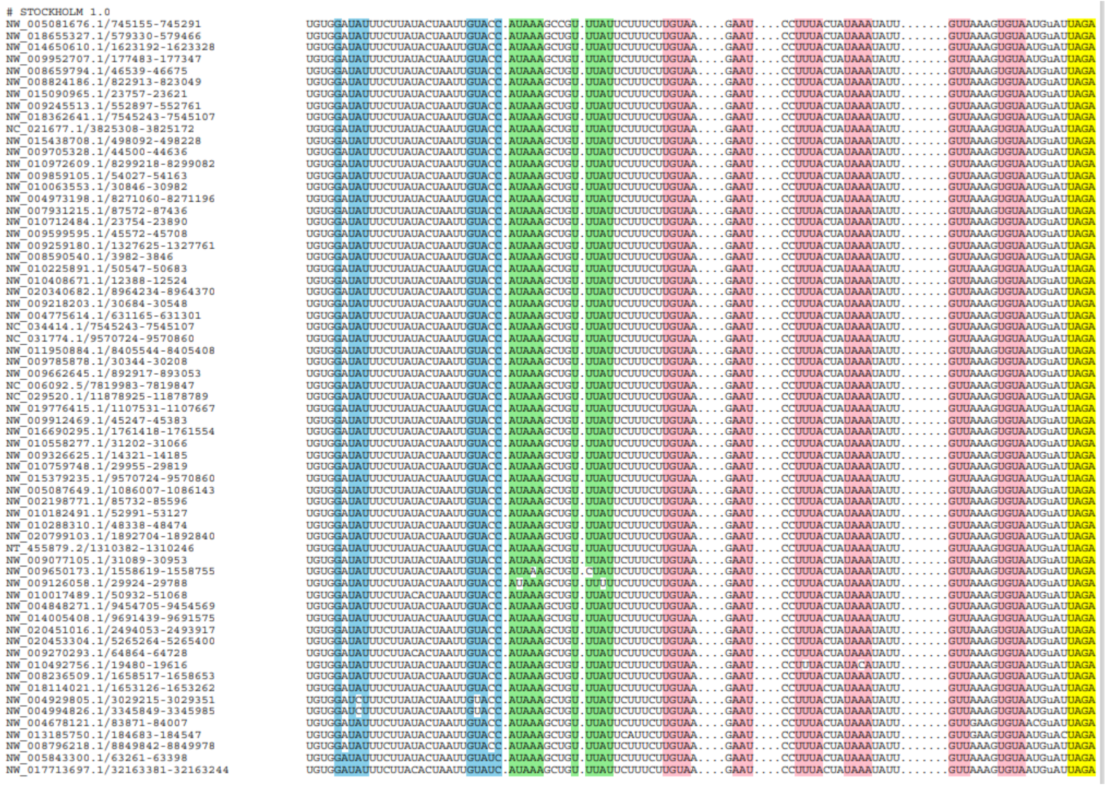

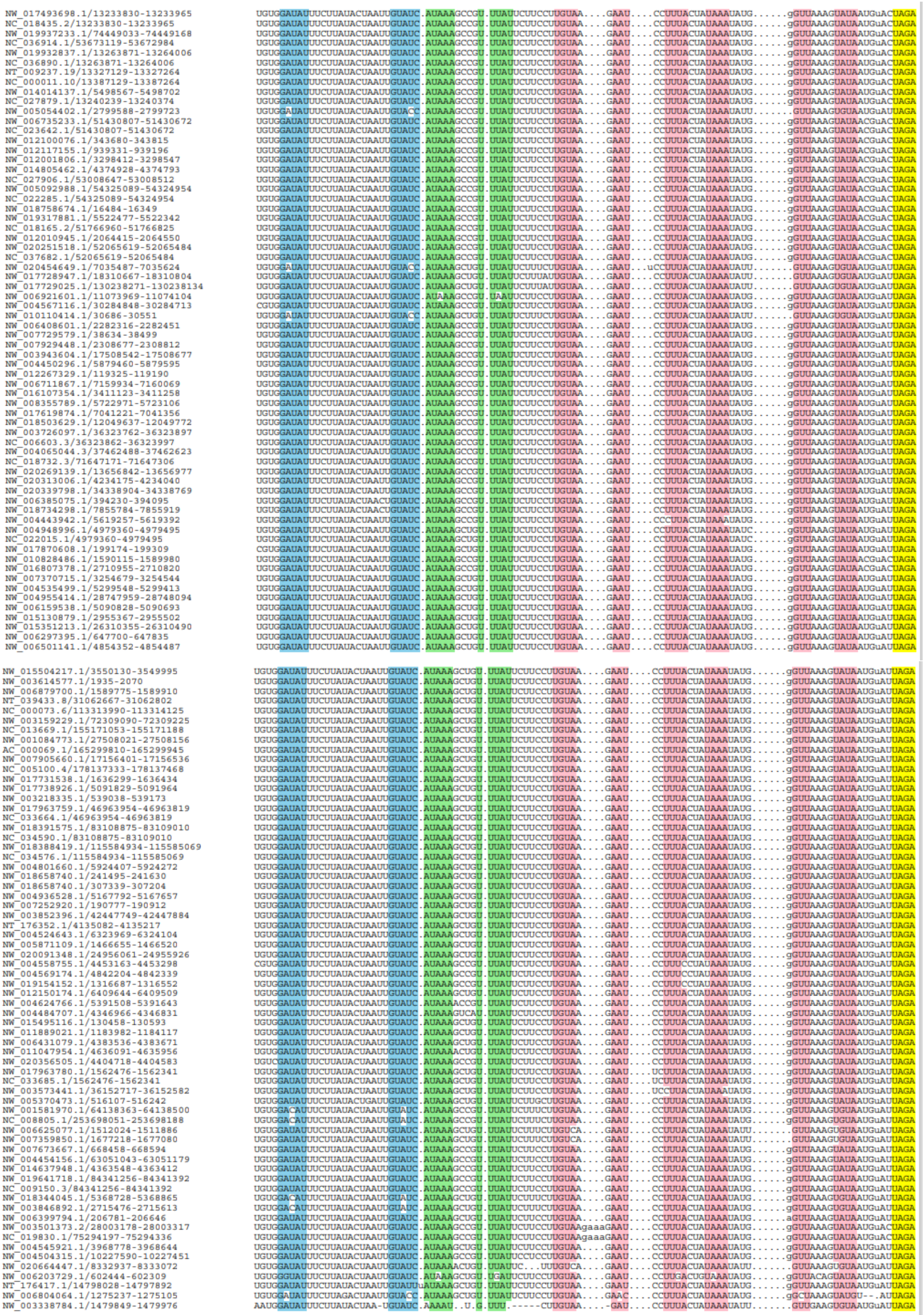

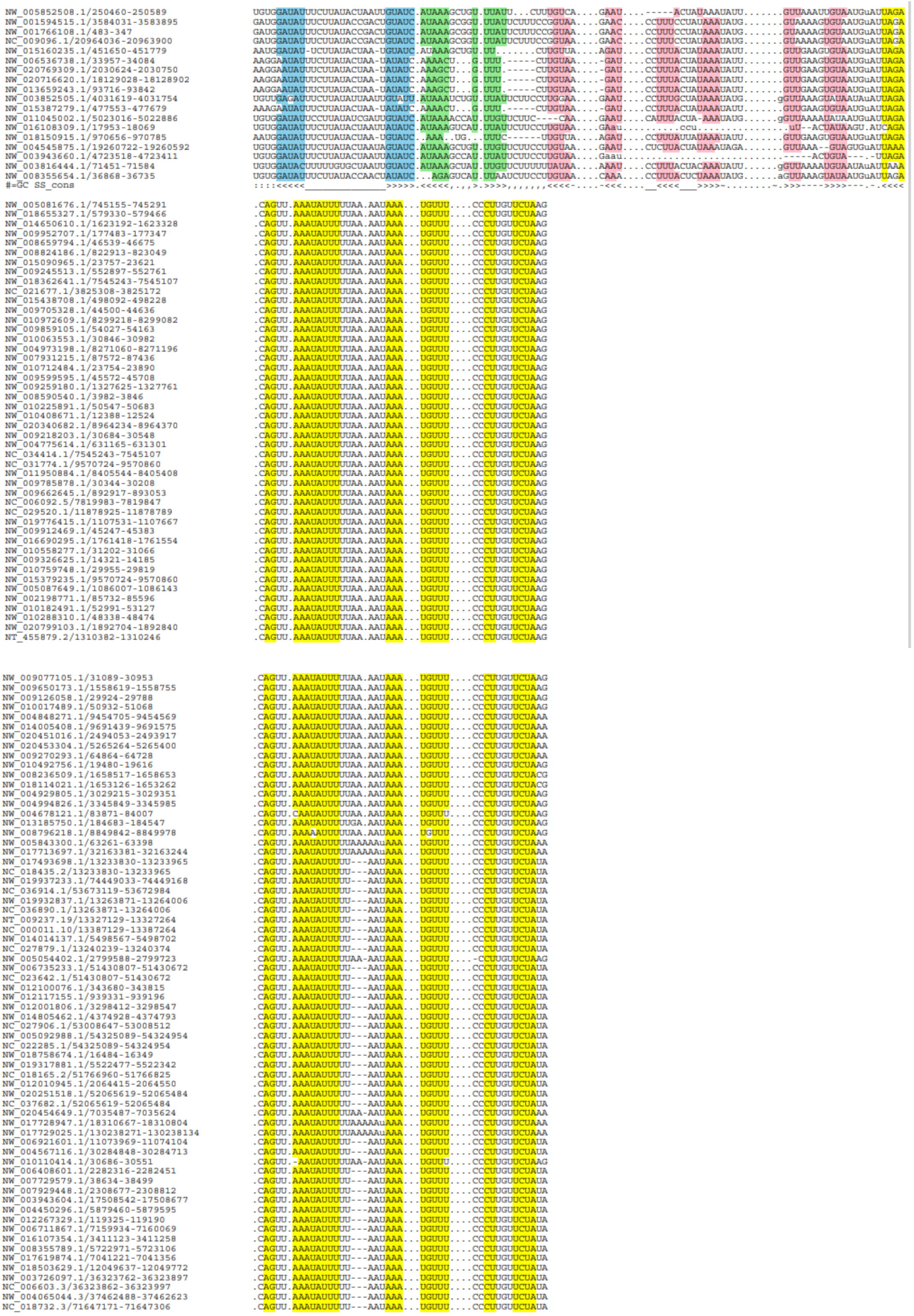

```
NW_020269139.1/13656842-13656977          .CAGUU.AAAUAUUUUU---AAUAAA...UGUUU....CCCUUGUUCUAUA
NW_020313006.1/4234175-4234040            .CAGUU.AAAUAUUUUU---AAUAAA...UGUUU....CCCUUGUUCUAUA
NW_020339798.1/34338904-34338769          .CAGUU.AAAUAUUUUU---AAUAAA...UGUUU....CCCUUGUUCUAUA
NW_006385075.1/394230-394095              .CAGUU.AAAUAUUUUU---AAUAAA...UGUUU....CCCUUGUUCUAUA
NW_018734298.1/7855784-7855919            .CAGUU.AAAUAUUUUU---AAUAAA...UGUUU....CCCUUGUUCUAUA
NW_004443942.1/5619257-5619392            .CAGUU.AAAUAUUUUU---AAUAAA...UGUUU....CCCUUGUUCUAUA
NW_004948996.1/4979360-4979495            .CAGUU.AAAUAUUUUU---AAUAAA...UGUUU....CCCUUGUUCUAUA
NC_022015.1/4979360-4979495               .CAGUU.AAAUAUUUUU---AAUAAA...UGUUU....CCCUUGUUCUAUA
NW_017870608.1/199174-199309              .CAGUU.AAAUAUUUUU---AAUAAA...UGUUU....CCCUUGUUCUAUA
NW_010828486.1/1590115-1589980            .CAGUU.AAAUAUUUUU---AAUAAA...UGUUU....UCCUUGUUCUAUA
NW_016807378.1/2710955-2710820            .CAGUU.AAAUAUUUUU---AAUAAA...UGUUU....UCCUUGUUCUAUA
NW_007370715.1/3254679-3254544            .CAGUU.AAAUAUUUUU---AAUAAA...UGUUU....CCCUUGUUCUAUA
NW_004535499.1/5299548-5299413            .CAGUU.AAAUAUUUUU---AAUAAA...UGUUU....CCCUUGUUCUAUA
NW_004955414.1/28747959-28748094          .CAGUU.AAAUAUUUUU---AAUAAA...UGUUU....CCCUUGUUCUAUA
NW_006159538.1/5090828-5090693            .CAGUU.AAAUAUUUUU---AAUAAA...UGUUU....CCCUUGUUCUAUA
NW_015130879.1/2955367-2955502            .CAGUU.AAAUAUUUUU---AAUAAA...UGUUU....CCCUUGUUCUAUA
NW_015351213.1/26310355-26310490          .CAGUU.AAAUAUUUUU---AAUAAA...UGUUU....CCCUUGUUCUAUA
NW_006297395.1/647700-647835              .CAGUU.AAAUAUUUUU---AAUAAA...UGUUU....CCCUUGUUCUAUA
NW_006501141.1/4854352-4854487            .CAGUU.AAAUAUUUUU---AAUAAA...UGUUU....CCCUUGUUCUAUA
NW_015504217.1/3550130-3549995            .CAGUU.AAAUAUUUUU---AAUAAA...UGUUU....CCCUUGUUCUAUA
NW_003614577.1/1935-2070                  .CAGUU.AAAUAUUUUU---AAUAAA...UGUUU....CCCUUGUUCUAUA
NW_006879700.1/1589775-1589910            .CAGUU.AAAUAUUUUU---AAUAAA...UGUUU....CCCUUGUUCUAUA
NT_039433.8/31062667-31062802             .CAGUU.AAAUAUUUUU---AAUAAA...UGUUU....CCCUUGUUCUAUA
NC_000073.6/113313990-113314125           .CAGUU.AAAUAUUUUU---AAUAAA...UGUUU....CCCUUGUUCUAUA
NW_003159229.1/72309090-72309225          .CAGUU.AAAUAUUUUU---AAUAAA...UGUUU....CCCUUGUUCUAUA
NC_013669.1/155171053-155171188           .CAGUU.AAAUAUUUUU---AAUAAA...UGUUU....CCCUUGUUCUAUA
NW_001084773.1/27508021-27508156          .CAGUU.AAAUAUUUUU---AAUAAA...UGUUU....CCCUUGUUCUAUA
AC_000069.1/165299810-165299945           .CAGUU.AAAUAUUUUU---AAUAAA...UGUUU....CCCUUGUUCUAUA
NW_007905660.1/17156401-17156536          .CAGUU.AAAUAUUUUU---AAUAAA...UGUUU....CCCUUGUUCUAUA
NC_005100.4/178137333-178137468           .CAGUU.AAAUAUUUUU---AAUAAA...UGUUU....CCCUUGUUCUAUA
NW_017731538.1/1636299-1636434            .CAGUU.AAAUAUUUUU---AAUAAA...UGUUU....CCCUUGUUCUAUA
NW_017738926.1/5091829-5091964            .CAGUU.AAAUAUUUUU---AAUAAA...UGUUU....CCCUUGUUCUAUA
NW_003218335.1/539038-539173              .CAGUU.AAAUAUUUUU---AAUAAA...UGUUU....CCCUUGUUCUAUA
NW_017963759.1/46963954-46963819          .CAGUU.AAAUAUUUUU---AAUAAA...UGUUU....CCCUUGUUCUAUA
NC_033664.1/46963954-46963819             .CAGUU.AAAUAUUUUU---AAUAAA...UGUUU....CCCUUGUUCUAUA
NW_018391575.1/83108875-83109010          .CAGUU.AAAUAUUUUU---AAUAAA...UGUUU....CCCUUGUUCUAUA
NC_034590.1/83108875-83109010             .CAGUU.AAAUAUUUUU---AAUAAA...UGUUU....CCCUUGUUCUAUA
NW_018388419.1/115584934-115585069        .CAGUU.AAAUAUUUUU---AAUAAA...UGUUU....CCCUUGUUCUAUA
NC_034576.1/115584934-115585069           .CAGUU.AAAUAUUUUU---AAUAAA...UGUUU....CCCUUGUUCUAUA
NW_004801660.1/5924407-5924272            .CAGUU.AAAUAUUUUU---AAUAAA...UGUUU....CCCUUGUUCUAUA
NW_018658740.1/241495-241630              .CAGUU.AAAUAUUUUU---AAUAAA...UGUUU....CCCUUGUUCUAUA
NW_018658740.1/307339-307204              .CAGUU.AAAUAUUUUU---AAUAAA...UGUUU....CCCUUGUUCUAUA
NW_004936528.1/5167792-5167657            .CAGUU.AAAUAUUUUU---AAUAAA...UGUUU....CCCUUGUUCUAUA
NW_007252920.1/190777-190912              .CAGUU.AAAUAUUUUU---AAUAAA...UGUUU....CCCUUGUUCUAUA
NW_003852396.1/42447749-42447884          .CAGUU.AAAUAUUUUU---AAUAAA...UGUUU....CCCUUGUUCUAUA
NT_176352.1/4135082-4135217               .CAGUU.AAAUAUUUUU---AAUAAA...UGUUU....CCCUUGUUCUAUA
NW_004524643.1/6323969-6324104            .CAGUU.AAAUAUUUUU---AAUAAA...UGUUU....CCCUUGUUCUAUA
NW_005871109.1/1466655-1466520            .CAGUU.AAAUAUUUUU---AAUAAA...UGUUU....CCCUUGUUCUAUA
NW_020091348.1/24956061-24955926          .CAGUU.AAAUAUUUUU---AAUAAA...UGUUU....CCCUUGUUCUAUA
NW_004558755.1/4453163-4453298            .CAGUU.AAAUAUUUUU---AAUAAA...UGUUU....CCCUUGUUCUAUA
NW_004569174.1/4842204-4842339            .CAGUU.AAAUAUUUUU---AAUAAA...UGUUU....CCCUUGUUCUAUA
NW_019154152.1/1316687-1316552            .CAGUU.AAAUAUUUUU---AAUAAA...UGUUU....CCCUUGUUCUAUA
NW_012150174.1/6409644-6409509            .CAGCU.AAAUAUUUUU---AAUAAA...UGUUU....CCCUUGUUCUAUA
NW_004624766.1/5391508-5391643            .CAGUU.AAAUAUUUUU---AAUAAA...UGUUU....CCCUUGUUCUAUA
NW_004484707.1/4346966-4346831            .CAGUU.AAAUAUUUUU---AAUAAA...UGUUU....CCCUUGUUCUAUA
NW_015495116.1/130458-130593              .CAGUU.AAAUAUUUUU---AAUAAA...UGUUU....CCCUUAUUCUAUA
NW_011889021.1/1183982-1184117            .CAGUU.AAAUAUUUUU---AAUAAA...UGUUU....CCCUUAUUCUAUA
NW_006431079.1/4383536-4383671            .CAGUU.AAAUAUUUUU---AAUAAA...UGUUU....CCCUUAUUCUAUA
NW_011047954.1/4636091-4635956            .CAGUU.AAAUAUUUUU---AAUAAA...UGUUU....CCCUUGUUCUAUA
NW_020356505.1/4404718-4404583            .CAGUU.AAAUAUUUUU---AAUAAA...UGUUU....CCCUUGUUCUAUA
NW_017963780.1/1562476-1562341            .CAGUU.AAAUAUUUUU---AAUAAA...UGUUU....CCCUUGUUCUAUA
NC_033685.1/1562476-1562341               .CAGUU.AAAUAUUUUU---AAUAAA...UGUUU....CCCUUGUUCUAUA
NW_003573441.1/36152717-36152582          .CAGUU.AAAUAUUUUU---AAUAAA...UGUUU....CCCUUGUUCUAUA
NW_005370473.1/516107-516242              .CAGUU.AAAUAUUUUU---AAUAAA...UGUUU....CCCUUGUUCUAUA
NW_001581970.1/64138363-64138500          .CAGUU.AAAUAUUUUUAA-AAUAAA...UGUUU....CCUUUGUUCUAUA
NC_008805.1/253698051-253698188           .CAGUU.AAAUAUUUUUAA-AAUAAA...UGUUU....CCUUUGUUCUAUA


NW_006625077.1/1512024-1511886            .CAGUU.AAAUAUUUUUAA-AAUaaA.UAUGUUU....CCCUUGUUCUAAA
NW_007359850.1/1677218-1677080            .CAGUU.AAAUAUUUUUAA-AAUaaA.UAUGUUU....CCCUUGUUCUAAA
NW_007673667.1/668458-668594              .CAGUUaAAAUAUUUUU---AAUAAA...UGUUU....CCCUUGUUCUAUA
NW_004454156.1/63051043-63051179          .CAGUUaAAAUAUUUUU---AAUAAA...UGUUU....CCCUUGUUCUAUA
NW_014637948.1/4363548-4363412            .CAGUUaAAAUAUUUUU---AAUAAA...UGUUU....CCCUUGUUCUAUA
NW_019641718.1/84341256-84341392          .CAGUUaAAAUAUUUUU---AAUAAA...UGUUU....CCCUUGUUCUAUA
NC_009150.3/84341256-84341392             .CAGUUaAAAUAUUUUU---AAUAAA...UGUUU....CCCUUGUUCUAUA
NW_018344045.1/5368728-5368865            .CAGUU.AAAUAUUUUUAA-AAUAAA...UGUUU....CCUUUGUUCUAUA
NW_003846892.1/2715476-2715613            .CAGUU.AAAUAUUUUUAA-AAUAAA...UGUUU....CCUUUGUUCUAUA
NW_006399794.1/206781-206646              .CAGUU.ACAUAUUUUU---AAUAAA...UGUUU....CCCUUGUUCUAUA
NW_003501373.2/28003178-28003317          .CAGUU.AAAUAUUUUU---AAUAAA...UGUUU....CCCUUGUUCUAUA
NC_019830.1/75294197-75294336             .CAGUU.AAAUAUUUUU---AAUAAA...UGUUU....CCCUUGUUCUAUA
NW_004545921.1/3968778-3968644            .CAGUU.AAAUAUUUUU---AAUAAA...UGUUU....-CCUUGUUCUAUA
NW_004504315.1/10227590-10227451          .CAGUU.AAAUAUUUUU---AAUAAA...UGUUUcuuuCCCUUGUUCUAUA
NW_020664447.1/8332937-8333072            .CAGUU.AAAUAUUUUUAA-AAUAAA.UAUGUUU....CCCUUGUUCUAAA
NW_006203729.1/602444-602309              .CAGUU.AAAUAUUUUU---AAAAAA...UGUUU....CCUUUGUUCUAUA
NT_176417.1/14798028-14797892             .CAGUU.AAAUAUUUUU---AAUAAA...UGUUU....CCCUUGUUCUAUA
NW_006804064.1/1275237-1275105            .CAGGU.AAAUAUUUUU---AAUAAA...UGUCU....CCCUUGUUCUAUA
NW_003338784.1/1479849-1479976            .CAGUG.AAAUAUUUUUAA-AAUAAA..AUGUUU...uCCCUUAUUCUGAA
NW_005852508.1/250460-250589              aCAGUU.AAAUAUUUUUAA-AAUAAA...UGUUU....CCCUUGUUCUAAA
NW_001594515.1/3584031-3583895            .CAGUU.GAAUAUUUUUAA-AAUAAA...CGUCG....CCCUUGUUGUAUA
NW_001766108.1/483-347                    .CAGUU.GAAUAUUUUUAA-AAUAAA...CGUCG....CCCUUGUUGUAUA
NC_009096.1/20964036-20963900             .CAGUU.GAAUAUUUUUAA-AAUAAA...CGUCG....CCCUUGUUGUAUA
NW_015160235.1/451650-451779              .CGGUU.GAAUAUUUUUAA-AAUAAA..AUAUUU...uCCCUUGUUCUAAA
NW_006536738.1/33957-34084                .CAGUU.AAAUAUUUUUAA-AAUAAA..AUGUUU...uCUCUUGUUCUAAA
NW_020769309.1/2030624-2030750            .CAGUU.AAAUAUUUUUAA-AAUAAA..AUGUUU....UCUUUGUUCUAAA
NW_020716620.1/18129028-18128902          .CAGUU.AAAUAUUUUUAA-AAUAAA..AUGUUU....UCUUUGUUCUAAA
NW_013659243.1/93716-93842                .CAGUU.AAAUAUUUUUAA-AAUAAA..AUGUUU....UCUUUGUUCUAAA
NW_003852505.1/4031619-4031754            .CAGGU.AGAUAUUUUU---AAUAAA...UAUUU....CCCUCGUUCUAUU
NW_015387279.1/477553-477679              .CAGUU.AAAUAUUUUUAA-AAUAAA..AUGUUU....UCUUUGUUCUAAA
NW_011045002.1/5023016-5022886            .CAGUU.AAAUAUUUUU---AAUAAA...UGUUU....CCCUUGUUCUAUA
NW_016108309.1/17953-18069                .CAGUU.AAAUAUUUUA---AAUAAA...UGUUU....CCCUUGUUCUAUA
NW_018150915.1/970656-970785              .CAGUU.AAAUAUUUUUAA-AAUAAAAUAUGUUUu..uCCCUUGUUCUACA
NW_004545875.1/19260722-19260592          .CAGUU.AAAUAUUUUU---AAUAAG...UGCCU....C-CU-AUUUUAUA
NW_003943660.1/4723518-4723411            .CAGUU.AAAUAUUUUA---AAUAAA...UGUUU....CCCUUAUUCUGUA
NW_003816444.1/71451-71584                .CAGUU.--AUAUUUUC---AAUAAA...UGUUU....CCCUUGUUCCACU
NW_008355654.1/36868-36735                .GAGUU.AAAUAUUUGU---AAUGAG...UGCUU....UCCUUGUUAAAAA
#=GC SS_cons                              .-<<--.<<<<<<<<_______->>>...>>>>>....-->>--->>>>.:
//
```

j *CD247-2015*

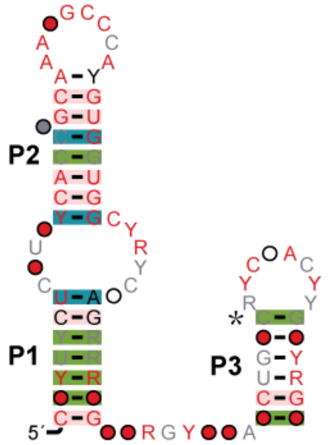

Sequence alignment for the motif *CD247-2015*

```
# STOCKHOLM 1.0
NT_004487.20/24249351-24249275          CACUUCUCAUC...CCAGCU-GCAAAUGCCcAUGUGGUGGCCAUC..AGA--GGUGAAGGCGAACCUGGCugUC-ACCCAGCCGGG
NG_007384.1/89672-89748                 CACUUCUCAUC...CCAGCU-GCAAAUGCCcAUGUGGUGGCCAUC..AGA--GGUGAAGGCGAACCUGGCugUC-ACCCAGCCGGG
NC_000001.11/167433938-167433862        CACUUCUCAUC...CCAGCU-GCAAAUGCCcAUGUGGUGGCCAUC..AGA--GGUGAAGGCGAACCUGGCugUC-ACCCAGCCGGG
NC_027868.1/146652032-146651956         CACUUCUCAUC...CCAGCU-GCAAACGCCcAUGUGGUGGCCAUC..AGA--GGUGAAGGCGAACCUGGCugUC-ACCCAGCCGGG
NW_014013792.1/5773354-5773278          CACUUCUCAUC...CCAGCU-GCAAACGCCcAUGUGGUGGCCAUC..AGA--GGUGAAGGCGAACCUGGCugUC-ACCCAGCCGGG
NC_036879.1/142472353-142472277         CACUUCUCAUC...CCAGCU-GCAAACGCCcAUGUGGUGGCCAUC..AGA--GGUGAAGGCGAACCUGGCugUC-ACCCAGCCGGG
NC_018424.2/146421475-146421399         CACUUCUCAUC...CCAGCU-GCAAACGCCcAUGUGGUGGCCAUC..AGA--GGUGAAGGCGAACCUGGCugUC-ACCCAGCCGGG
NW_019932792.1/61058881-61058957        CACUUCUCAUC...CCAGCU-GCAAACGCCcAUGUGGUGGCCAUC..AGA--GGUGAAGGCGAACCUGGCugUC-ACCCAGCCGGG
NW_017493678.1/22130877-22130801        CACUUCUCAUC...CCAGCU-GCAAACGCCcAUGUGGUGGCCAUC..AGA--GGUGAAGGCGAACCUGGCugUC-ACCCAGCCGGG
NW_012113808.1/7691416-7691340          CACUUCUCAUC...CCAGCU-GCAAACGCCcAUGUGGUGGCCAUC..AGA--GGUGAAGGCGAACCUGGCagUC-ACCCAGUCGGG
NW_019317079.1/4327655-4327579          CACUUCUCAUC...CCAGCU-GCAAACGCCcAUGUGGUGGCCAUC..AGA--GGUGAAGGCGAACCUGGCagUC-ACCCAGUCGGG
NW_006785816.1/1297020-1296941          CCUUCCUCCUA...UCA-CCUGCAAAAGCCcAUGUGGUGGCUGCCagAGGCAAAGGUUAAUCCAGCUGUC.aUCCAUUU-GAUAGU
NC_036903.1/82952969-82953045           CACUUCUCAUC...CCA-GCUGCAAACGCCcAUGUGUUGGCCAUC..AGA--GGUGAAGGCGAACCUGACugUC-ACCCAGUCGGG
NW_019937203.1/19709154-19709078        CACUUCUCAUC...CCA-GCUGCAAACGCCcAUGUGUUGGCCAUC..AGA--GGUGAAGGCGAACCUGACugUC-ACCCAGUCGGG
NW_019873626.1/1415407-1415328          CCUUCCUCCUA...UCA-CCUGCAAAAGCCcAUGUGGUGGCUGCCagAGGCAAAGGUUAAUCCAUCUGUC.aUCCAUUU-GAUAGU
NW_005370460.1/3423383-3423310          CAC--CUAUUCguaCCA-CCUGCAAAAGCC.AUGUGGUGGCCACA.gAG----GUGAAGGCAACCCAGCU..UCUACCC-AUCGGG
NW_014804585.1/2858556-2858632          CACUUCUCAUC...CCAGCG-GCAAACGCCcAUGUGGUGGCCAUC..AGA--GGUGAAGGCGAACCUGGCagUC-ACCCAGUCGGG
NW_005092959.1/25399408-25399332        CACUUCUCAUC...CCAGCG-GCAAACGCCcAUGUGGUGGCCAUC..AGA--GGUGAAGGCGAACCUGGCagUC-ACCCAGUCGGG
NW_018753644.1/1089176-1089252          CACUUCUCAUC...CCAGCG-GCAAACGCCcAUGUGGUGGCCAUC..AGA--GGUGAAGGCGAACCUGGCagUC-ACCCAGUCGGG
NC_027893.1/200245346-200245422         CACUUCUCAUC...CCAGCG-GCAAACGCCcAUGUGGUGGCCAUC..AGA--GGUGAAGGCGAACCUGGCagUC-ACCCAGUCGGG
NW_020251504.1/198078050-198078126      CACUUCUCAUC...CCAGCG-GCAAACGCCcAUGUGGUGGCCAUC..AGA--GGUGAAGGCGAACCUGGCagUC-ACCCAGUCGGG
NC_018152.2/193198324-193198400         CACUUCUCAUC...CCAGCG-GCAAACGCCcAUGUGGUGGCCAUC..AGA--GGUGAAGGCGAACCUGGCagUC-ACCCAGUCGGG
NC_022272.1/25399408-25399332           CACUUCUCAUC...CCAGCG-GCAAACGCCcAUGUGGUGGCCAUC..AGA--GGUGAAGGCGAACCUGGCagUC-ACCCAGUCGGG
NW_012011511.1/4474036-4473960          CACUUCUCAUC...CCAGCG-GCAAACGCCcAUGUGGUGGCCAUC..AGA--GGUGAAGGCGAACCUGGCagUC-ACCCAGUCGGG
NC_037668.1/198078050-198078126         CACUUCUCAUC...CCAGCG-GCAAACGCCcAUGUGGUGGCCAUC..AGA--GGUGAAGGCGAACCUGGCagUC-ACCCAGUCGGG
NW_020173022.1/398230-398151            CCUUCCUCCUA...UCA-CCUGCAAAAGCCcACGUGGUGGCCGCCagAGGCAAAGGUUAGUCCAGCUGUC.aUCCAUUU-GAUAGU
NW_012002568.1/3377501-3377577          CACUUCUCAUC...CCA-GCAGCAAACGCCcAUGUGGUGGCCAUC..AGA--GGUGAAGGCGAACCUGGCagUC-ACCCAGUUGGG
NW_004438446.1/1315844-1315765          CCUUCCUCCUA...UCA-CCUGCAAAAGCCcACGUGGUGGCUGCCagAGGCAAAGGUUAGUCCAGCUAUC.aUCCAUUU-GAUAGU
NW_005871327.1/199610-199537            CAC--CUAUUCguaCCA-CCUGCAAAAGCC.AUGUGGUGGCCGCA.gAG----GUGAAGGCAACCCAGCU..CCUACCC-AUCGGG
NW_006291020.1/2503137-2503210          CAC--CUAUUCguaCCA-CCUGCAAAAGCC.AUGUGGUGGCUGCA.gAG----GUGAAGGCAACCCAGCU..CCUACCC-AUCGGG
NW_004087897.1/35211031-35211107        CACUCCUCAUC...CCA-GCUGCAAACGCCcAUGUGCUGGCCAUC..AGA--GGUGAAGGUGAACCUGGCugUC-ACCCAGUUGGG
NC_019827.1/35212031-35212107           CACUCCUCAUC...CCA-GCUGCAAACGCCcAUGUGCUGGCCAUC..AGA--GGUGAAGGUGAACCUGGCugUC-ACCCAGUUGGG
NW_012102110.1/1664335-1664259          CACUUCUCAUC...CCAGCG-GCAAACGCCcAUGUGGUGGCCAUC..AGA--GGUGAAGGUGAACCUGGCagUC-ACCCAGUUGGG
NW_019160964.1/1039948-1039869          CCUUCCUCCUA...UCA-CCUGCAAAAGCCcACGUGGUGGCUGCCagGGGCAAAGGUUAGUCCAGCUGUC.aUCCAUUU-GAUAGU
NW_006725799.1/1376528-1376449          CCUUCCUCCCA...UCA-CCUGCAAAAGCCcACGUGGUGGCUGCCagAGGCAAAGGUUAAUCCAGCUGUC.aUCCAUUU-GAUAGU
NW_003943631.1/1895282-1895206          CACUUCUCAUC...CCAGCU-GCAAAUGCCcAAGUGGUGGCCAUC..AGA--GGUGAAGGCGAACCUGGCugUC-ACCCAGUCAGA
NW_016107399.1/3818981-3818905          CACUUCUCAUC...CCAGCU-GCAAAUGCCcAAGUGGUGGCCAUC..AGA--GGUGAAGGCGAACCUGGCugUC-ACCCAGUCAGA
NW_006735275.1/60593220-60593296        CACUUCUCAUC...CCAGCG-GCAAACGCCcAUGUGGUGGCCAUC..AGA--GGUGAAGGCGAACCUGGCagUC-ACCCAGUAGGG
NW_007370735.1/3314954-3314881          CACC--UAUUCauaCCA-CCUGCAAAAGCC.ACGUGGUGGCCGCA.gA----GGUGAAGGUAACCCAGCU..CCUACCC-AUCGGG
NW_011591336.1/4050669-4050590          CCUUCCUCUGA...UCA-CCUGCAAAAGCCcAUGUGGUGGCUGGCagAGGCAGAGGUUAGUCCAGCUCUC.aCCCACUG-GAUAGU
NW_006212689.1/857921-857842            CCUUCCUCUGA...UCA-CCUGCAAAAGCCcAUGUGGUGGCUGGCagAGGCAGAGGUUAGUCCAGCUCUC.gCCCACUG-GAUAGU
NW_011513397.1/4141137-4141058          CCUUCCUCUGA...UCA-CCUGCAAAAGCCcAUGUGGUGGCUGGCagAGGCAGAGGUUAGUCCAGCUCUC.gCCCACUG-GAUAGU
#=GC SS_cons                            <<<<<<<----...<<<-<<-<<<_______._>>>>>>>>-----..>>>--->>>>-------<<<<<<.._________>>>>>>
#=GC RF                                 cccccuCuUc...CcAuCCugcAAAuGCC.AUguGGUgGCcAcc..aggagggggAagGCaAAcCuGgC..UCCAuCcAGcCaGg
//
```

k *CAPN6-33096*

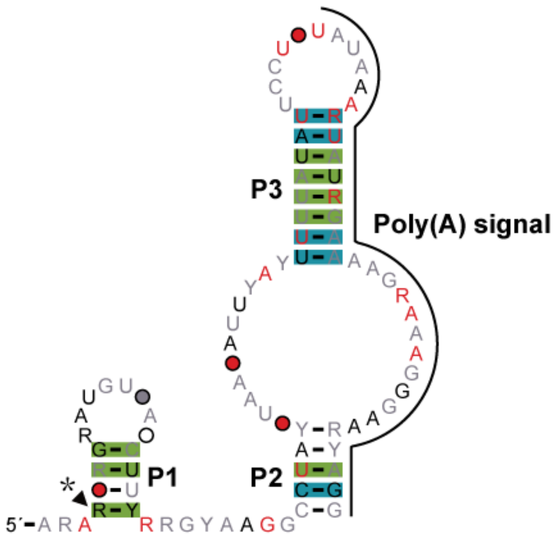

Sequence alignment for the motif *CAPN6-33096*

```
# STOCKHOLM 1.0
NT_011651.18/48785195-48785105       GCAGAGGAugggAUGUCAA..................CU.U..C...AAGCAAG.GCCUACAUUAGAUUCAUUUUCAUAU...UCCUAUAUAA.AAUAUGGAAAAGGAAAGGG
NC_000023.11/111247737-111247647     GCAGAGGAugggAUGUCAA..................CU.U..C...AAGCAAG.GCCUACAUUAGAUUCAUUUUCAUAU...UCCUAUAUAA.AAUAUGGAAAAGGAAAGGG
NG_015962.1/27810-27900              GCAGAGGAugggAUGUCAA..................CU.U..C...AAGCAAG.GCCUACAUUAGAUUCAUUUUCAUAU...UCCUAUAUAA.AAUAUGGAAAAGGAAAGGG
NC_018447.2/111309409-111309319      GCAGAGGAugggAUGUCAA..................CU.U..C...AAGCAAG.GCCUACAUUAGAUUCAUUUUCAUAU...UCCUAUAUAA.AAUAUGGAAAAGGAAAGGG
NW_017493719.1/49256128-49256038     GCAGAGGAugggAUGUCAA..................CU.U..C...AGGCAAG.GCCUACAUUAGAUUCAUUUUCAUAU...UCCUAUAUAA.AAUAUGGAAAAGGAAAGGG
NC_036926.1/105846736-105846646      GCAGAGGAugggAUGUCAA..................CU.U..C...AGGCAAG.GCCUACAUUAGAUUCAUUUUCAUAU...UCCUAUAUAA.AAUAUGGAAAAAGAAAGGG
NW_019937265.1/28034642-28034552     GCAGAGGAugggAUGUCAA..................CU.U..C...AGGCAAG.GCCUACAUUAGAUUCAUUUUCAUAU...UCCUAUAUAA.AAUAUGGAAAAAGAAAGGG
NW_014014456.1/2481849-2481759       GCAGAGGAugggAUGUCAA..................CU.U..C...AGGCAAA.GCCCACAUUAGAUUCAUUUUCAUAU...UCCUAUAUAA.AAUAUGGAAAAGGAAAGGG
NC_027891.1/110825184-110825094      GCAGAGGAugggAUGUCAA..................CU.U..C...AGGCAAA.GCCCACAUUAGAUUCAUUUUCAUAU...UCCUAUAUAA.AAUAUGGAAAAGGAAAGGG
NC_036902.1/106707811-106707721      GCAGAGGAugggAUGUCAA..................CU.U..C...AGGCAAA.GCCCACAUUAGAUUCAUUUUCAUAU...UCCUAUAUAA.AAUAUGGAAAAGGAAAGGG
NW_019932880.1/4461087-4461177       GCAGAGGAugggAUGUCAA..................CU.U..C...AGGCAAA.GCCCACAUUAGAUUCAUUUUCAUAU...UCCUAUAUAA.AAUAUGGAAAAGGAAAGGG
NC_019841.1/98545513-98545423        GCAGAGGGugggAUGUCAA..................CU.U..C...AGGCAAG.GCCUACAUUAGAUUCAUUUUCAUAU...UCCUAUAUAA.AAUAUGGAAAAGGAAAGGG
NW_004087912.1/63573773-63573683     GCAGAGGGugggAUGUCAA..................CU.U..C...AGGCAAG.GCCUACAUUAGAUUCAUUUUCAUAU...UCCUAUAUAA.AAUAUGGAAAAGGAAAGGG
NW_012102798.1/3235993-3236083       GCAGAGGAugggAUGUCAA..................CU.U..C...AGGCAAG.GCCUACAAUAGAUUCAUUUUCAUAU...UCCUAUACAA.AAUAUGGAAAAGGAAAGGG
NW_018760875.1/43032-42942           GCAGAGGAugggAUGUCAA..................CU.U..C...AGGCAAG.GCCUACAAUAGAUUCAUUUUCAUAU...UCCUAUACAA.AAUAUGGAAAAGGAAAGGG
NW_010826423.1/5133-5043             GCAGAGGAugggAUGUCAA..................CU.U..C...AGGCAAG.GCCUACAAUAGAUUCAUUUUCAUAU...UCCUAUACAA.AAUAUGGAAAAGGAAAGGG
NW_019363966.1/7326381-7326291       GCAGAGGAugggAUGUCAA..................CU.U..C...AGGCAAG.GCCUACAAUAGAUUCAUUUUCAUAU...UCCUAUACAA.AAUAUGGAAAAGGAAAGGG
NW_014806008.1/2765266-2765176       GCAGAGGAugggAUGUCAA..................CU.U..C...AGGCAAG.GCCUACAAUAGAUUCAUUUUCAUAU...UCCUAUACAA.AAUAUGGAAAAGGAAAGGG
NC_037689.1/109178154-109178064      GCAGAGGAugggAUGUCAA..................CU.U..C...AGGCAAG.GCCUACAAUAGAUUCAUUUUCAUAU...UCCUAUACAA.AAUAUGGAAAAGGAAAGGG
NW_012116533.1/2901746-2901656       GCAGAGGAugggAUGUCAA..................CU.U..C...AGGCAAG.GCCUACAAUAGAUUCAUUUUCAUAU...UCCUAUACAA.AAUAUGGAAAAGGAAAGGG
NC_018172.2/100737374-100737284      GCAGAGGAugggAUGUCAA..................CU.U..C...AGGCAAG.GCCUACAAUAGAUUCAUUUUCAUAU...UCCUAUACAA.AAUAUGGAAAAGGAAAGGG
NW_020251525.1/109178154-109178064   GCAGAGGAugggAUGUCAA..................CU.U..C...AGGCAAG.GCCUACAAUAGAUUCAUUUUCAUAU...UCCUAUACAA.AAUAUGGAAAAGGAAAGGG
NW_012011256.1/4519979-4519889       GCAGAGGAugggAUGUCAA..................CU.U..C...AGGCAAG.GCCUACAAUAGAUUCAUUUUCAUAU...UCCUAUACAA.AAUAUGGAAAAGGAAAGGG
NW_005093003.1/49238767-49238677     GCAGAGGAugggAUGUCAA..................CU.U..C...AGGCAAG.GCCUACAAUAGAUUCAUUUUCAUAU...UCCUAUACAA.AAUAUGGAAAAGGAAAGGG
NW_016825472.1/93626-93716           GCAGAGGAugggAUGUCAA..................CU.U..C...AGGCAAG.GCCUACAAUAGAUUCAUUUUCAUAU...UCCUAUACAA.AAUAUGGAAAAGGAAAGGG
NC_027913.1/105276455-105276365      GCAGAGGAugggAUGUCAA..................CU.U..C...AGGCAAG.GCCUACAAUAGAUUCAUUUUCAUAU...UCCUAUACAA.AAUAUGGAAAAGGAAAGGG
NC_022292.1/108342903-108342813      GCAGAGGAugggAUGUCAA..................CU.U..C...AGGCAAG.GCCUACAAUAGAUUCAUUUUCAUAU...UCCUAUACAA.AAUAUGGAAAAGGAAAGGG
NW_018505261.1/4154303-4154393       GCAGAGGAugugAUGUCAA..................CU.U..C...ACGCAAG.GCCUACGUUAAAUUCAUUUUCAUAU...UCCUAUGUAA.AAUGUGGAAAAGGAAAGGG
NW_006735280.1/99054735-99054645     GCAGAGGAugggAUGUCAA..................CU.U..C...AGGCAAG.ACCUAUAAUAGAUUCAUUUUCAUAU...UCCUAUACAA.AAUAUGGAAAAGGAAAGGG
NW_005357414.1/322465-322548         AAAAUGGA....AUGUAAA..................CU.A..U...GGGAAAG.GCCUAGAUAAUAUUUA-UUUUAUAU...UCCUUUACAA.AAUGUAUGG--AAAAAAGG
NW_006291724.1/214588-214671         AAAAUGGA....AUGUAAA..................CU.A..U...GGGAAAG.GCCUAGAUAAUAUUUA-UUUUAUAU...UCCUUUAUAA.AGUGUAUGG--AAAAAAGG
NW_012002290.1/10367114-10367204     GCAGAGGAugggAUGUCAA..................CU.U..C...AGGCAAG.ACCUACGAUAGAUUCACUUUCAUAU...UCCUAUACAA.AAUAUGGAAAAGGAAAGGG
NW_003943682.1/4020849-4020939       GCAGAAGAugggAUGUCAA..................CU.U..C...ACAUAAG.GCCUACAUUAGAUUCAUUUUCAUAU...UUCUAUGUAA.AAUAUGGAAAAGUAAAGGG
NW_004454246.1/4424337-4424248       AGAACGGG....AUGUAAA...............cuuCU.U..U...GGGCAAG.GCCUACAUAAUAUUCAUUUUUAUAU...UCCUAUAUAA.AGUAUAGAAAAGAAAAGGG
NW_005871050.1/24601115-24601198     AAAAUGGA....AUGUAAA..................CU.A..U...GGGAAAG.GCCUAGAUAAUAUUUA-UUUUAUAU...UCUUUUAUAA.AAUGUAUGG--AAAAAAGG
NW_006793272.1/1144805-1144727       AAAGUCU-....-UU----..................GG.G..C...AA--AGG.GCCUAUCUAAUACUCAUUUCUGUAU...CCCUAUAUAA.AGUAUAGAAAAGAAAAGGG
NW_019160952.1/3657996-3658073       AAAGUCU-....-UG----..................GG.G..C...AA---GG.GCCUAUCUAAUAUUCAUUUCUAUAU...CCCUAUAUAA.AGUAUAGAAAAGAAAAGGG
NW_004438560.1/4108637-4108560       AAAGUCU-....-UG----..................GG.G..C...AA---GG.GCCUAUCUAAUAUUCAUUUCUAUAU...CCCUAUAUAA.AGUAUAGAAAAGAAAAGGG
NW_017844263.1/13277652-13277729     AAAGUCU-....-UG----..................GG.G..C...AA---GG.GCCUAUCUAAUAUUCAUUUCUAUAU...CCCUAUAUAA.AGUAUAGAAAAGAAAAGGG
NW_019873575.1/4119299-4119202       AAAAGAGA....AA-----augggauauaaaagu...CU.C..U...GGGCAAGgGCCUAUCUAAUAUUCAUUUCUAUAU...CCCUAUAUAA.AGUGUAGAAAAGAAAAGGG
NW_016107607.1/2966731-2966821       GCAGAGGAugggAUGUCAA..................CU.U..C...ACACAAG.GCCUACAGUAGAUUCAUUUACAUAU...UUCUAUGUAA.AAUAUGGAAAAGGAAAUGG
NW_020090627.1/8228891-8228802       AAAAUUGA....AUGUAAA..................CU.U..UuuuGGGCAAG.GCCUACAUAAUAUUCAUUUUUACAU...UCCUUUAUAA.AAUGUAGAAAAGAAUAGGG
NC_013918.1/99751247-99751157        GCAAAGGAugggAUGUCAA..................CU.U..C...ACACAAG.GCCUACAGUAGAUUCAUUUUCAUAU...UCCUAUGUAA.AAUACGGAAAAGGAAAGGG
NW_003185010.1/6969721-6969631       GCAAAGGAugggAUGUCAA..................CU.U..C...ACACAAG.GCCUACAGUAGAUUCAUUUUCAUAU...UCCUAUGUAA.AAUACGGAAAAGGAAAGGG
NC_009175.3/92038195-92038106        AGAAUGGG....AUGUAAA...............cuuCU.U..U...GGGCAAG.GGCUACGUAAUAUUCACUUUUGUAU...UCCUAUAUAA.AGUAUAGAAAAGAAAAGGG
NW_007676750.1/135769-135680         AGAAUGGG....AUGUAAA...............cuuCU.U..U...GGGCAAG.GGCUACGUAAUAUUCACUUUUGUAU...UCCUAUAUAA.AGUAUAGAAAAGAAAAGGG
NW_019641743.1/92038195-92038106     AGAAUGGG....AUGUAAA...............cuuCU.U..U...GGGCAAG.GGCUACGUAAUAUUCACUUUUGUAU...UCCUAUAUAA.AGUAUAGAAAAGAAAAGGG
NW_014636815.1/4697079-4696990       AGAAUGGG....AUGUAAA...............cucCU.U..U...GGGCAAG.GGCUACGUAAUAUUCACUUUUGUAU...UCCUAUAUAA.AGUAUAGAAAAGAAAAGGG
NW_015503913.1/1976930-1977017       AAAAUGGA....AUGUAAA..................CU.U..U...GUGCAAG.GCCUACAUAAUAUUUAUUUUUAUAUuauUCCUUUAUAA.AGUGUAGGG--GAAAAGGG
NW_011889025.1/2147904-2147815       AGAAUGGG....AUGUAAA...............cuuCU.U..U...GGGCAAG.GCCUACAUAAUAUUUAUUUUUAUAU...UUUUUUAUAA.AGUAUAAAAAAGAAAAGGG
NW_012134303.1/7901798-7901709       AAAAUGGG....AUGUCAA...............cuuCU.U..U...GAGCCAG.GCCUACAUAAGAUUAAUUUUUAUGU...UCCUAUAUAA.AAUAUGGAAAAGGACAAGG
NW_006727420.1/597287-597364         AAAGUCU-....-UU----..................GG.G..C...AA---GG.GACUAUCUAACAUUCAUUUCUAUAU...CCCUAUAUAA.AGUAUAGAAGAGAAAAGGG
NW_011046271.1/3433272-3433183       AAAAUGGG....AUGUGA-uc...........uu...CU.U..U...GGGCAAG.GUCUAUGUAAUACUUAUUUUUAUAU...UCCUAUAUGA.AAUAUAGAAAAGGAAAGGG
NW_006440619.1/1329216-1329128       AGAAUGGG....AUGUAAA...............cuuCU.U..U...GGGCAAG.GCCUACAUAAUAUUUAUUUUUAUAU...UUUUUUAUAA.AGUAUAAAAA-GAAAAGGG
NC_033692.1/39682841-39682752        AGAAUGGG....AUGUCAA...............cuuCU.U..U...GGGCCAG.GCCUACAUAAGAUUAAUUUUUAUAU...UCUUAUAUAA.AAUAUGGAAACAGACAAGG
NW_017963787.1/39682841-39682752     AGAAUGGG....AUGUCAA...............cuuCU.U..U...GGGCCAG.GCCUACAUAAGAUUAAUUUUUAUAU...UCUUAUAUAA.AAUAUGGAAACAGACAAGG
NW_007727736.1/196458-196368         AGACUGGG....AUGUAA-ac..........uuc...UU.U..G...GGGCAAG.GCUUACAUAAUGCUCACUUUUGUAU...UCCUGUAUAA.AAUACAGGAAAGGACAGGG
NW_018335532.1/1107103-1107014       AAAAUGGG....AUAUAA-gu...........gu...CU.U..U...GGGCAAG.GACUAUCUAGUAUUCAUUUUUAUAU...CCUUCUAUAA.AAUAUAGAAAAGAAAAGGA
NW_004492586.1/278119-278205         AGAAUGGG....AUGUAAU..................CU.U..U...AUGUAAG.ACCUCCAUAAUAUUCAUCUUCAUAU...UUUUAUAUAC.AAUAUGGAAAAAGAAAGAG
NW_006213166.1/246792-246881         AAAAUGGG....AUGUAA-gc...........uu...CU.U..U...GCGCAAG.GCCUAGCUAGUAUUUAUUUUUCUAU...UCCUAUAUAA.AGUAUAGAGAAGAAAAGGG
NW_011515754.1/479976-480065         AAAAUGGG....AUGUAA-gc...........uu...CU.U..U...GCGCAAG.GCCUAGCUAGUAUUUAUUUUUCUAU...UCCUAUAUAA.AGUAUAGAGAAGAAAAGGG
NW_011592095.1/653314-653225         AAAAUGGG....AUGUAA-gc...........uu...CU.U..U...GCGCAAG.GCCUAGCUAGUAUUUAUUUUUCUAU...UCCUAUAUAA.AGUAUAGAGAAGAAAAGGG
NC_037569.1/82671117-82671206        AAAAUGGG....AUAUAA-gu...........gu...CU.U..U...GGGGGAG.GCCUAUCUAGUAUUCAUUUUUAUAU...CCUUCUAUAA.AAUAUAGAAAAGAAAAGGA
NW_020228707.1/82671117-82671206     AAAAUGGG....AUAUAA-gu...........gu...CU.U..U...GGGGGAG.GCCUAUCUAGUAUUCAUUUUUAUAU...CCUUCUAUAA.AAUAUAGAAAAGAAAAGGA
NW_005394717.1/231602-231513         AAAAUGGG....AUAUAA-ga...........gu...CU.U..U...GGGGGAG.GCCUAUCUAGUAUUCAUUUUUAUAU...CCUUCUAUAA.AAUAUAGAAAAGAAAAGGA
```

```
NW_017844738.1/37479660-37479571      AAAAUGGG....AUAUAA-ga...........gu...CU.U..U...GGGGGAG.GCCUAUCUAGUAUUCAUUUUUAUAU...CCUUCUAUAA.AAUAUAGAAAAGAAAAGGA
NC_037357.1/59416333-59416244         AAAAUGGG....AUAUAA-ga...........gu...CU.U..U...GGGGGAG.GCCUAUCUAGUAUUCAUUUUUAUAU...CCUUCUAUAA.AAUAUAGAAAAGAAAAGGA
NW_020190114.1/59416333-59416244      AAAAUGGG....AUAUAA-ga...........gu...CU.U..U...GGGGGAG.GCCUAUCUAGUAUUCAUUUUUAUAU...CCUUCUAUAA.AAUAUAGAAAAGAAAAGGA
NW_011493689.1/18334-18245            AAAAUGGG....AUAUAA-ga...........gu...CU.U..U...GGGGGAG.GCCUAUCUAGUAUUCAUUUUUAUAU...CCUUCUAUAA.AAUAUAGAAAAGAAAAGGA
NC_032679.1/37479660-37479571         AAAAUGGG....AUAUAA-ga...........gu...CU.U..U...GGGGGAG.GCCUAUCUAGUAUUCAUUUUUAUAU...CCUUCUAUAA.AAUAUAGAAAAGAAAAGGA
NW_005882909.1/620419-620508          AAAAUGGG....AUAUAA-gc...........uu...CU.U..U...GCGCAAG.GCCUAGCUAGUAUUUAUUUUUCUAU...UCCUAUAUAA.AGUAUAGAGAAGAAAAGGG
NW_004955490.1/4520334-4520423        AAAAUGGG....AUGUAAA...............cuuCU.U..U...GGGCAAG.AUCUACGUAAUACAUAUUUUUAUAU...UCAUAUAUGA.AAUAUGAAAAAGGAAAGGG
NW_007370845.1/419872-419793          AAAAUGGA....AUGUAAA..................CU.C..U...GGGAAAG.GUCUAGAUAAUAUUUA-UUUUAUAU...UCCU----AA.AGUGUAGGG--AAUAAAGG
NW_011943567.1/197295-197384          AAAAUGGG....AUAUAA-gu...........gu...CU.U..U...GGGGGAG.GCCUGUCUAGUAUUCAUUUUUAUAU...CCUUCUAUAA.AAUAUAGAAAAGAAAAGGA
NC_019484.2/117260829-117260918       AAAAUGGG....AUAUAA-gu...........gu...CU.U..U...GGGGGAG.GCCUGUCUAGUAUUCAUUUUUAUAU...CCUUCUAUAA.AAUAUAGAAAAGAAAAGGA
NW_014639036.1/117260829-117260918    AAAAUGGG....AUAUAA-gu...........gu...CU.U..U...GGGGGAG.GCCUGUCUAGUAUUCAUUUUUAUAU...CCUUCUAUAA.AAUAUAGAAAAGAAAAGGA
NW_003852662.1/414373-414463          AGAAUGGG....ACGUCAA..................CUuAuuU...GGGCCAG.GCCUACAUGAGAUUAACUUUUAUAU...UUCUCUAUAAaAAUAUGGAAAAGGAAAAGG
NT_176373.1/9406132-9406225           AAAGAAGAugggAUGUAAA..................CU.U..CuuuGGGCAAG.AUCUAUACAAUACUUAGUUUUAUAU...UCCUAUACGA.AAUAUGGAAAAGGAAAGGA
NW_017189517.1/32819094-32819183      AAAAUGGG....AUAUAA-gu...........gu...CU.U..U...GGGGGAG.GCCUGUCUAGUAUUCAUUUUUAUAU...CCUUCUAUAA.AAUAUAGAAGAGAAAAGGA
#=GC SS_cons                          :::<<<<_....._______~~~~~~~~~~~~~~~...>>.>..>...-------.-<<<<<-----------<<<<<<<<...__________._>>>>>>>>----------
#=GC RF                               aaAGucgA....AUgUaAA~~~~~~~~~~~~~~~...cg.a..C...aaGaAAG.GCCuacaUaAuAuUcAUuucuauAu...uCCUaUAuAA.AaUauagaaAAgaAAAgGG

NT_011651.18/48785195-48785105        AAGUGGG
NC_000023.11/111247737-111247647      AAGUGGG
NG_015962.1/27810-27900               AAGUGGG
NC_018447.2/111309409-111309319       AAGUGGG
NW_017493719.1/49256128-49256038      AAGUGGG
NC_036926.1/105846736-105846646       AAGUGGG
NW_019937265.1/28034642-28034552      AAGUGGG
NW_014014456.1/2481849-2481759        AAGUGGG
NC_027891.1/110825184-110825094       AAGUGGG
NC_036902.1/106707811-106707721       AAGUGGG
NW_019932880.1/4461087-4461177        AAGUGGG
NC_019841.1/98545513-98545423         AAGUGGG
NW_004087912.1/63573773-63573683      AAGUGGG
NW_012102798.1/3235993-3236083        AAGUGGG
NW_018760875.1/43032-42942            AAGUGGG
NW_010826423.1/5133-5043              AAGUGGG
NW_019363966.1/7326381-7326291        AAGUGGG
NW_014806008.1/2765266-2765176        AAGUGGG
NC_037689.1/109178154-109178064       AAGUGGG
NW_012116533.1/2901746-2901656        AAGUGGG
NC_018172.2/100737374-100737284       AAGUGGG
NW_020251525.1/109178154-109178064    AAGUGGG
NW_012011256.1/4519979-4519889        AAGUGGG
NW_005093003.1/49238767-49238677      AAGUGGG
NW_016825472.1/93626-93716            AAGUGGG
NC_027913.1/105276455-105276365       AAGUGGG
NC_022292.1/108342903-108342813       AAGUGGG
NW_018505261.1/4154303-4154393        AAGUGGG
NW_006735280.1/99054735-99054645      AAGUGGG
NW_005357414.1/322465-322548          AACCAGG
NW_006291724.1/214588-214671          AACCAGG
NW_012002290.1/10367114-10367204      AAGUGGG
NW_003943682.1/4020849-4020939        AAGUGGG
NW_004454246.1/4424337-4424248        AAGCAGG
NW_005871050.1/24601115-24601198      AACCAGG
NW_006793272.1/1144805-1144727        AAAUAGG
NW_019160952.1/3657996-3658073        AAAUAGG
NW_004438560.1/4108637-4108560        AAAUAGG
NW_017844263.1/13277652-13277729      AAAUAGG
NW_019873575.1/4119299-4119202        AAAUAGG
NW_016107607.1/2966731-2966821        AAGUGGG
NW_020090627.1/8228891-8228802        AAUCAGG
NC_013918.1/99751247-99751157         AAGUGGG
NW_003185010.1/6969721-6969631        AAGUGGG
NC_009175.3/92038195-92038106         AAGCAGG
NW_007676750.1/135769-135680          AAGCAGG
NW_019641743.1/92038195-92038106      AAGCAGG
NW_014636815.1/4697079-4696990        AAGCAGG
NW_015503913.1/1976930-1977017        AACCAGG

NW_011889025.1/2147904-2147815        AAGCAAG
NW_012134303.1/7901798-7901709        UAGUAGG
NW_006727420.1/597287-597364          AAAUAGG
NW_011046271.1/3433272-3433183        ACGAAGA
NW_006440619.1/1329216-1329128        AAGCAAG
NC_033692.1/39682841-39682752         UAGUAGG
NW_017963787.1/39682841-39682752      UAGUAGG
NW_007727736.1/196458-196368          AAGUAGG
NW_018335532.1/1107103-1107014        AAGUAGA
NW_004492586.1/278119-278205          AAAUAGG
NW_006213166.1/246792-246881          AAGUAAG
NW_011515754.1/479976-480065          AAGUAAG
NW_011592095.1/653314-653225          AAGUAAG
NC_037569.1/82671117-82671206         AAGUAGA
NW_020228707.1/82671117-82671206      AAGUAGA
NW_005394717.1/231602-231513          AAGUAGA
NW_017844738.1/37479660-37479571      AAGUAGA
NC_037357.1/59416333-59416244         AAGUAGA
NW_020190114.1/59416333-59416244      AAGUAGA
NW_011493689.1/18334-18245            AAGUAGA
NC_032679.1/37479660-37479571         AAGUAGA
NW_005882909.1/620419-620508          AAGUAAG
NW_004955490.1/4520334-4520423        AAGAAGA
NW_007370845.1/419872-419793          AGCCAGG
NW_011943567.1/197295-197384          AAGUAGA
NC_019484.2/117260829-117260918       AAGUAGA
NW_014639036.1/117260829-117260918    AAGUAGA
NW_003852662.1/414373-414463          AAGUAGG
NT_176373.1/9406132-9406225           AAGAAGA
NW_017189517.1/32819094-32819183      AAGUAGA
#=GC SS_cons                          -->>>>>
#=GC RF                               AAguaGG
//
```
