# Supplementary material for: Identification of 11 candidate structured noncoding RNA motifs in humans by comparative genomics
Source: BMC Genomics. 2021 Mar 9;22:164. doi: 10.1186/s12864-021-07474-9 (PMC7941889; doi:10.1186/s12864-021-07474-9)
Supplement: Supplementary file 24 — Additional file 24. Bioinformatics steps and configurations. [file 12864_2021_7474_MOESM24_ESM.pdf]

Step1: Use Perl program to extract the sequence except CDS region.
Step2: Create a masked BLAST database and Blast

Use WindowMasker to identifiy and mask out highly repetitive DNA sequences and DNA sequences with low complexity in a genome:

2.1 windowmasker -in hs_chr.fa -infmt fasta -mk_counts -parse_seqids -out hs_chr_mask.counts -sformat obinary

2.2 windowmasker -in hs_chr.fa -infmt fasta -ustat hs_chr.counts -outfmt maskinfo_asn1_bin -parse_seqids -out hs_chr_mask.asnb

2.3 makeblastdb -in hs_chr.fa -infmt fasta -dbtype nucl -parse_seqids -mask_data hs_chr_mask.asnb -out hs_chrdb -title genome

2.4 blastn -query ncRNA.fasta -db genome.db -task blastn -evalue 1e-6 -perc_identity 80 -db_soft_mask 30 -out human-db.out -outfmt 6

Step3:
blastn -query extncRNA.fasta -db Rfam_SINE_db -task blastn -evalue 1e-20 -max_target_seqs 1 -out extncRNA-known.out -outfmt 6
perl mksys-homo-rnacode0.01.pl (to make a system file to run RNAcode for all fasta files.)
perl sys-RNAcode-P0.01.pl (The perl contains two basic steps for each fasta file. Step 1: clustalw2 -infile=$name.fa -type=dna -output=clustal -outfile=$name.aln -align; step 2: RNAcode --outfile $name.gtf --gtf --best-only --cutoff 0.01 $name.aln)
Step4:
Perl createcmfiles-metazoa-part6.pl (to make a system file to run Cmfinder)
cmfinder04.pl ~/cm-file/part6cm76583.fasta -combine (run CMfinder)
Step5:
perl ~R2R/src/Undos.pl cm6.fa.motif.h1_1 (sometimes it needs to format the stockholm files before running the R2R. R2R is a program to draw the consensus structures automatically)
~/R2R/src/r2r --GSC-weighted-consensus cm6.fa.motif.h1_1.cons.sto 3 0.97 0.9 0.75 4 0.97 0.9 0.75 0.5 0.1
~/R2R/src/r2r cm6.fa.motif.h1_1.cons.sto f cm6.fa.motif.h1_1.pdf
Step6: to run Infernal
cmbuild cm6.fa.motif.h1_1.cm cm6.fa.motif.h1_1.processed.sto
cmcalibrate cm6.fa.motif.h1_1.cm
cmsearch --tblout results.tab cm6.fa.motif.h1_1.cm other.fna

Function analysis
RBPmap:
perl RBPmap.pl -input humam_motif.fa -db 'hg38' -db_motifs 'all_human' -stringency 'high' -conservation 'on' -job_name RBPmap-all
RegRNA2.0:
http://regrna2.mbc.nctu.edu.tw/
String:
https://string-db.org/
TCGA splicing seq
https://bioinformatics.mdanderson.org/TCGASpliceSeq/singlegene.jsp
