# Supplementary material for: Identification of 11 candidate structured noncoding RNA motifs in humans by comparative genomics
Source: BMC Genomics. 2021 Mar 9;22:164. doi: 10.1186/s12864-021-07474-9 (PMC7941889; doi:10.1186/s12864-021-07474-9)
Supplement: Supplementary file 25 — Additional file 25. Fig. S6. The full-length original agarose gel for Fig. S5b. [file 12864_2021_7474_MOESM25_ESM.pdf]

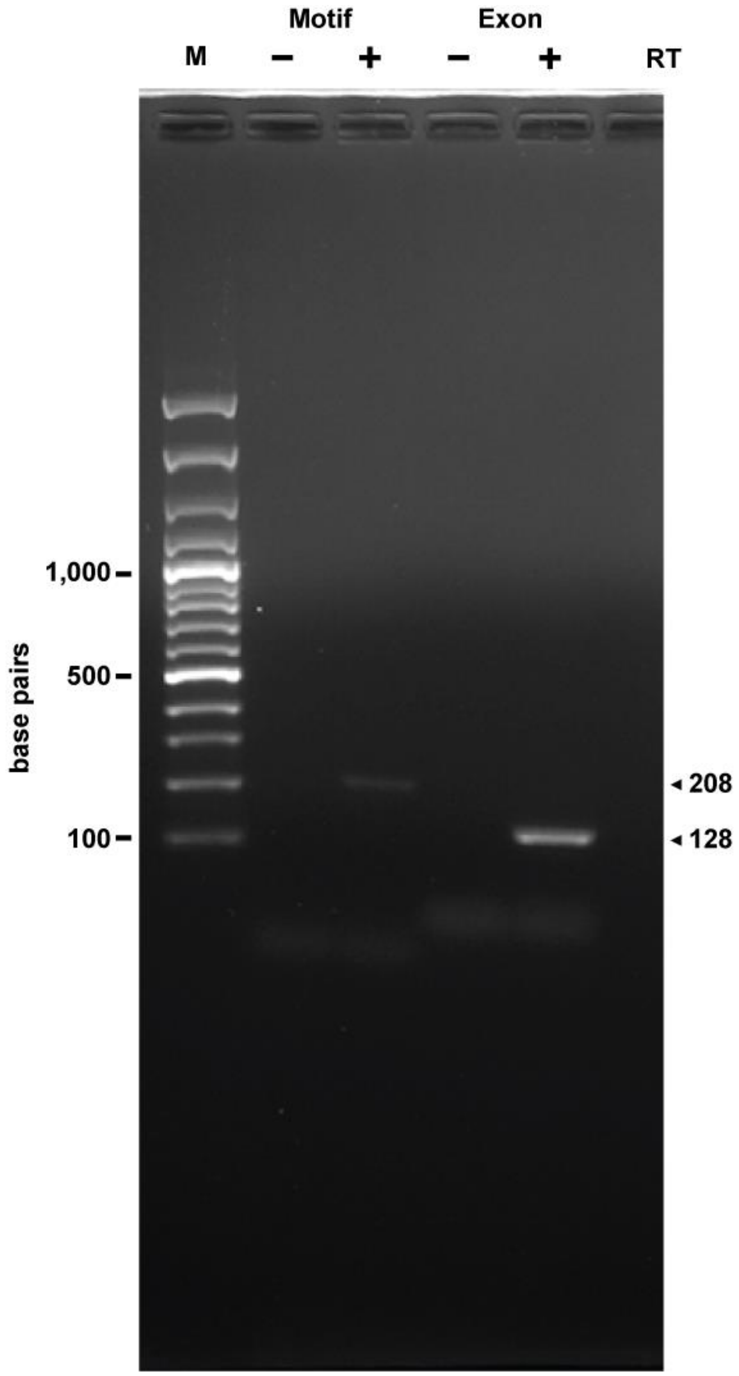

Fig. S6 The full-length original agarose gels for Fig. S5b. Agarose gel separation of RT-PCR products of *NPTN-6924* motif, generated by using primers for the RNA motif itself (Motif) and mRNA coding regions (Exon). M stands for DNA markers. Lanes containing PCR products are marked with (+) or (-), indicating the presence or absence of reverse transcriptase (RT). The two arrows next to the gel indicate bands corresponding to DNA products of the expected size. This gel picture is the full length

gel picture for the Fig. S5b.
